# Gauss’s law for networks directly reveals community boundaries

**DOI:** 10.1038/s41598-018-30401-0

**Published:** 2018-08-09

**Authors:** Ayan Sinha, David F. Gleich, Karthik Ramani

**Affiliations:** 1 School of Mechanical Engineering, Purdue University, West Lafayette, IN 47907 USA; 2 Department of Computer Science, Purdue University, West Lafayette, IN 47907 USA

## Abstract

The study of network topology provides insight into the function and behavior of physical, social, and biological systems. A natural step towards discovering the organizing principles of these complex topologies is to identify a reduced network representation using cohesive subgroups or communities. This procedure often uncovers the underlying mechanisms governing the functional assembly of complex networks. A community is usually defined as a subgraph or a set of nodes that has more edges than would be expected from a simple, null distribution of edges over the graph. This view drives objective such as modularity. Another perspective, corresponding to objectives like conductance or density, is that communities are groups of nodes that have extremal properties with respect to the number of internal edges and cut edges. Here we show that identifying community boundaries rather than communities results in a more accurate decomposition of the network into informative components. We derive a network analog of Gauss’s law that relates a measure of flux through a subgraph’s boundary to the connectivity among the subgraph’s nodes. Our Gauss’s law for networks naturally characterizes a community as a subgraph with high flux through its boundary. Aggregating flux over these boundaries gives rise to a Laplacian and forms the basis of our “Laplacian modularity” quality function for community detection that is applicable to general network types. This technique allows us to determine communities that are both overlapping and hierarchically organized.

## Introduction

We illustrate the efficacy of our method using an undirected, unweighted network^1,2^. Nevertheless, our framework for community detection is valid for directed, weighted, signed, and even multislice networks^3^. Hierarchical and overlapping organization of communities in most real-world networks suggests that there is no *best* partition as derived by quality functions. Instead there are several *good* partitions with varying degrees of overlap at different scales corresponding to various organizational levels of the network^4,5^. We demonstrate how the multiscale heat kernel^6^ extends our quality function to reveal these *good* partitions and provides a consolidated understanding of communities, their overlap, and relevant hierarchies.

**Figure 1:**
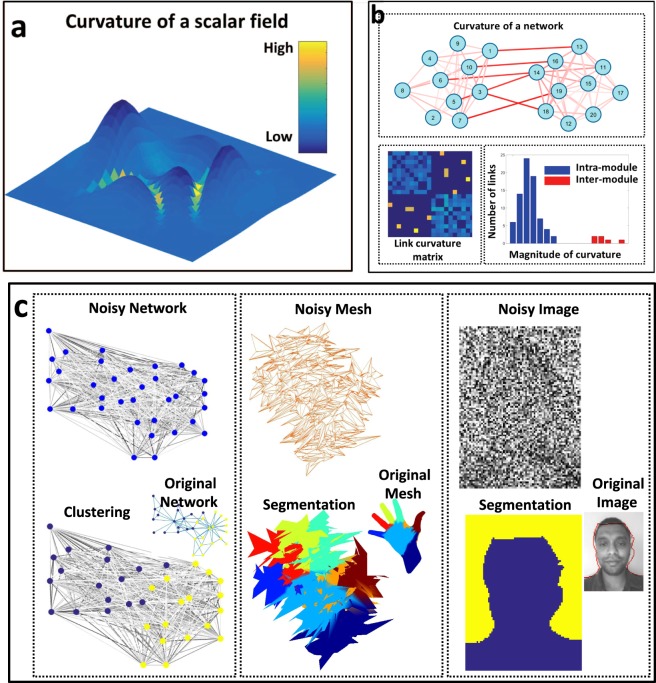
Identifying module boundaries improves detection. (**a**) The curvature (color) identifies the boundaries (high curvature) between the peaks that represent the values of the scalar field (height). (**b**) (Top) A curvature-like metric of a network shows the same effect wherein the curvature of inter-community links is higher (darker shades of red) relative to intra-community links. (Bottom Left) The curvature of links in the network are displayed in the link curvature matrix. The curvature increases as color changes from blue to yellow. (Bottom Right) The histogram of curvature values shows a gap between the curvature of intra-module and inter-module links allowing easy detection. (**c**) The same methodology is applicable to general graphs such as social networks, meshes and images and identifies module boundaries even in the presence of large noise. The identified modules are mapped to the corresponding noiseless graphs in the top-right corner for visual verification.

Although there is no consensus, a common abstraction is that communities are subgraphs with strong intra-subgraph cohesion and weak inter-subgraph cohesion, where cohesive strength is measured either in terms of direct connections or more sophisticated connectivity measures^7–9^. In this manuscript, we seek to use information about the boundary between subgraphs to identify the communities themselves. In the case of networks, the boundary information arises by generalizing the concept of a scalar field’s curvature to the links of a network (Fig. 1a,b, Eq. 3). This information reliably indicates the module structure even in the presence of substantial noise (Fig. 1c).

**Figure 2:**
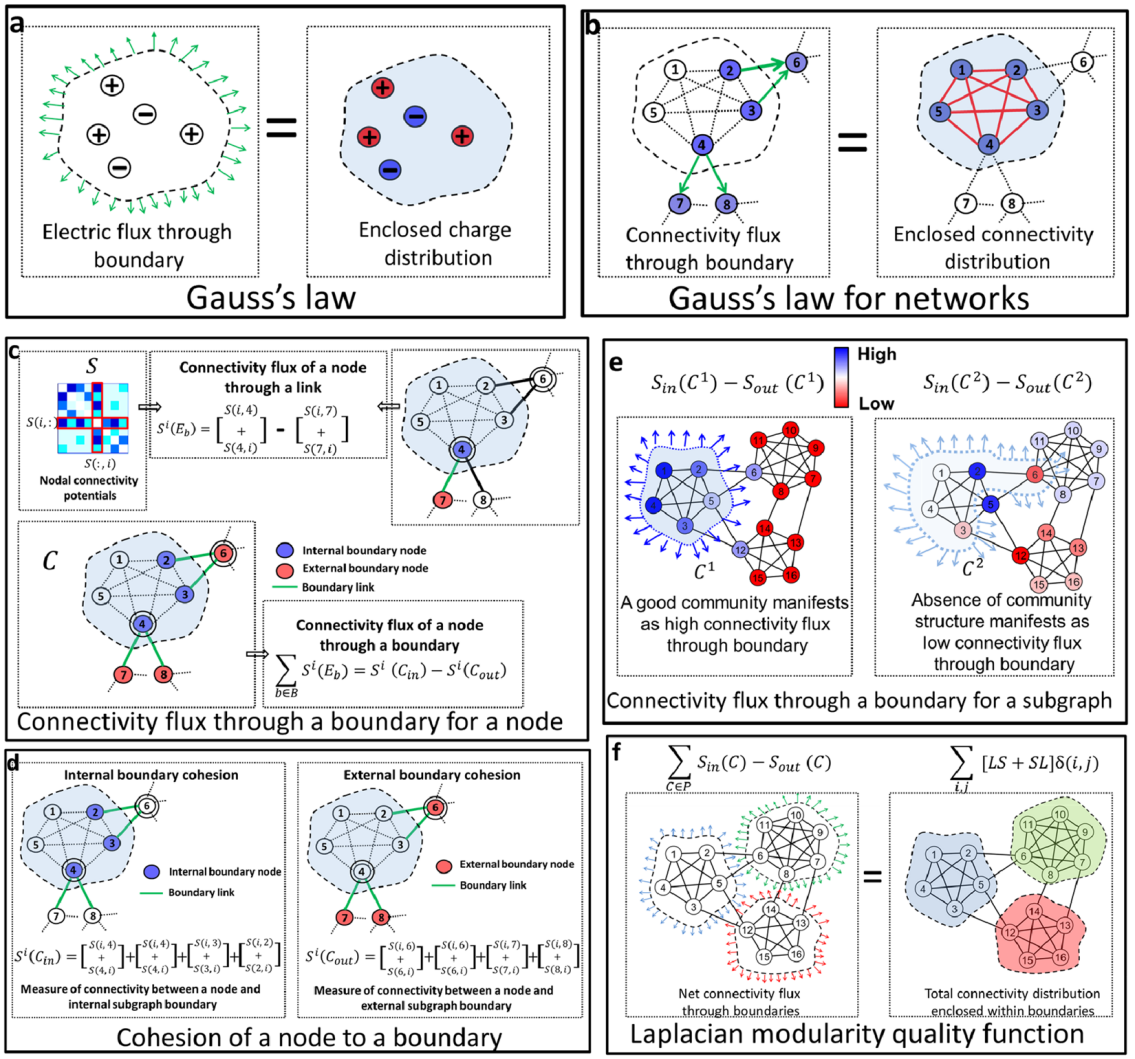
Community detection using Gauss’s Law. (**a**) Classical Gauss’s law. (**b**) Gauss’s law for networks implicitly measures the enclosed connectivity distribution as the connectivity flux through the boundary. (**c**) Evaluating connectivity flux through a boundary for a node in the network. (**d**) Connectivity flux quantified as measures of cohesion. (**e**) Connectivity flux through a boundary for a subgraph: High and low values of this connectivity flux indicate good communities or absence of community structure, respectively. The darker region of blue indicate higher connectivity flux. The color of nodes (blue to red) highlight the magnitude of connectivity flux (positive to negative) through the boundary. (**f**) Laplacian modularity quality function derived from the cumulative connectivity flux for a network partition.

Relationships between boundaries and their contents are common in physics such as how the well-known Gauss’s law relates the electric flux through a surface boundary to the charge enclosed within the surface (Fig. 2a). Our approach to detect communities boundaries uses a network analog of Gauss’s law to relate a flux through a subgraph’s boundary to the cohesion among the nodes of a subgraph (Fig. 2b). The method works with respect to a general measure of connectivity (or similarity) between all pairs of nodes encoded in matrix *S*. (Unlike original Gauss’s law, there is flexibility in the choice of *S*. All experiments in this manuscript set *S* to be the heat kernel, and the supplement discusses results for alternative choices of *S*). The element *S*(*i*,*j*) either gives the adjacency *A*(*i*,*j*) of nodes *i* and *j* in the network or an extended similarity of node *i* to *j* evaluated using a reference property such as common neighbors, path connectivity, etc.^10^; we call *S*(*i*,*j*) the connectivity potential of *i*,*j*. Given *S*, the flux through a subgraph boundary for a node, termed connectivity flux, mirrors the electric flux through a surface for an electrostatic potential field, albeit in a discrete setting (See Fig. 2c and Methods). A subgraph’s boundary comprises links that separate the subgraph from the rest of the network. Each boundary link has an internal and external node with respect to the subgraph. Thus, the set of boundary links induces two disjoint multisets (i.e., sets with repeated elements) of internal and external boundary nodes (see Methods). Consequently, the connectivity flux for a node *i* with respect to subgraph *C*’s boundary is equal to the difference of two terms, one measuring the connectivity of the node *i* to internal boundary nodes and one to external boundary nodes. We call them the internal *S*^*i*^(*C*_*in*_) and external *S*^*i*^(*C*_*out*_) boundary cohesion, respectively (see Methods). Note that because of the multisets, each cohesion expression has exactly the same number of terms (See Fig. 2d). The net connectivity flux *q*(*C*) through subgraph *C*’s boundary for the set of nodes constituting the subgraph, is then equal to $q(C)={\sum }_{i\in C}({S}^{i}({C}_{in})-{S}^{i}({C}_{out}))$, which we write as *q*(*C*) = *S*_*in*_(*C*) − *S*_*out*_(*C*). The decomposition of connectivity flux into internal and external components allows us to characterize communities in both a strong and weak sense^11^ (see Supplementary Material, section 2.1). High connectivity flux through the subgraph boundary, *q*(*C*) = *S*_*in*_(*C*) − *S*_*out*_(*C*) suggests a difference in the strength of internal and external cohesion of subgraph *C*, and consequently, a close-knit community (Fig. 2e).

The relationship between flux and communities motivates an additive quality function *Q*, to evaluate a partition ${\mathscr{P}}$ of nodes in a network:

1 $$Q=\sum _{C\in {\mathscr{P}}}q(C)=\sum _{C\in {\mathscr{P}}}({S}_{in}(C)-{S}_{out}(C\mathrm{)).}$$

**Figure 3:**
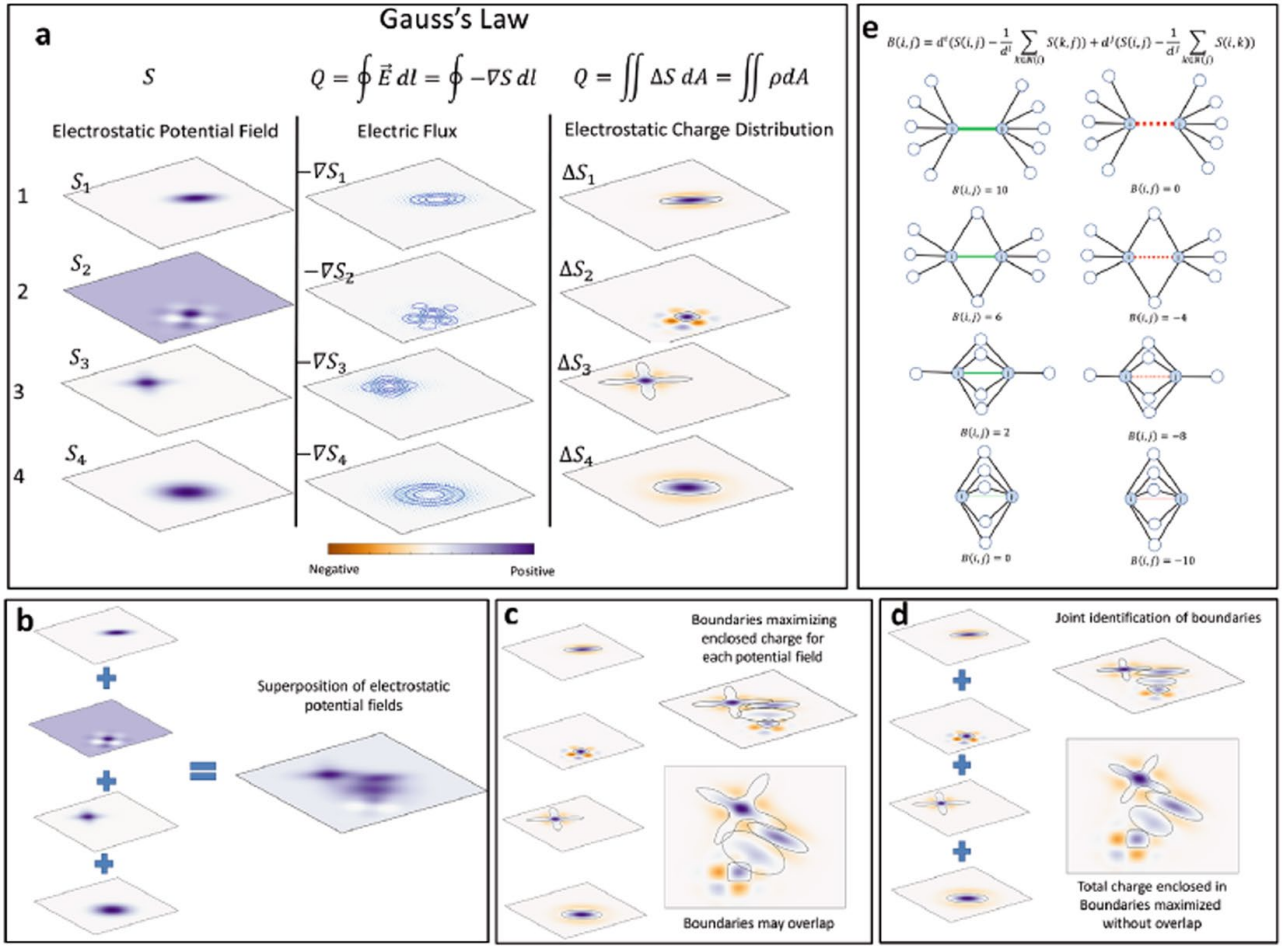
Partitioning a 2D domain using Gauss’s law. (**a**) Gauss’s law on a 2D domain. Left: Different electrostatic potential fields, *S*_*k*_, at various points on a 2D domain. Higher potentials appear as darker shades of purple. Center: The electric flux calculated as the gradient of the potential field, $-\nabla {S}_{k}$. Right: The isocontours of the electrostatic charge distribution, given as ${\rm{\Delta }}{S}_{k}$ due to Gauss’s law. Higher potentials appear as darker shades of purple, and the charge distribution varies from negative to positive as the color changes from orange to purple. The continuous 2D boundary maximizing the enclosed charge distribution is shown as a blue curve. (**b**) Superposition of the potential fields on the 2D domain. (**c**) Independently maximizing the enclosed positive charges gives rise to a soft partitioning of the 2D domain. (**d**) Collectively maximizing the charge distributions outputs a consistent partition without overlap. (**e**) Variation of curvature of a network. $S(i,j)$ is based on the adjacency information. The curvature of the red solid links or red dotted non-edges, $B(i,j)$ between nodes *i* and *j* depends on the number of common neighbors.

We clarify how our method relates to Gauss’s law by first illustrating the classical Gauss’s law in a manner that will serve our purpose of drawing analogies between Gauss’s law for electrostatics and our method for community detection. Assume there are independent electrostatic potential fields on a 2D domain (Fig. 3a, left). These potential fields capture a point’s influence on its 2D neighborhood. The gradient of the electrostatic potential defines the electric field as per classical electrostatics. The electric flux is then a measure of electric field flowing through a 2D boundary (Fig. 3a, center). Gauss’s law states that the electric flux flowing through a boundary is proportional to the total charge enclosed by the boundary. This then becomes the area integral of the charge distribution over the enclosed domain. The charge distribution is calculated as the divergence of the gradient of the potential field due to the Gauss’s theorem (Fig. 3a, right). As electrostatic potential is a scalar quantity, the net electrostatic potential over the 2D is a superposition of all potential fields on the 2D domain (Fig. 3b). The boundary maximizing the total electric potential is simply the boundary of the 2D domain as all potential values are positive over the domain. We can independently partition the 2D domain into 2D regions maximizing enclosed positive charge distribution for each potential field using Gauss’s law (Fig. 3c). However, this partition may not be a hard partition as some 2D regions may overlap each other. Hence, we jointly identify the boundaries or collectively maximize the individual charge distributions due to potential fields to obtain a consistent partition of the 2D domain (Fig. 3d).

Our main result, which is our network analog of Gauss’s law, is a reformulation of *Q* that relates its definition in terms of the flux through the boundary to the internal connectivity (Fig. 2f):

2 $$Q=\sum _{i,j}[LS+SL]\delta (i,j),$$

where *δ*(*i*, *j*) is 1 if *i* and *j* belong to the same community and 0 otherwise, *L* is a matrix known as the Laplacian, the discrete analogue of the Laplace operator (In the case of a weighted network, we interpret Eq. 2 with the weighted Laplacian to ensure the computation is continuous with respect to small edge weights), and *Q* is interpreted as the net charge within modules of the partition. This new formulation shows that *Q* possesses the key properties of the modularity quality function^12^ (see Supplementary Material, section 2.2 for derivation of Eq. 2). Hence, we call our quality function the *Laplacian modularity* and the matrix *B* = *LS* + *SL* the Laplacian modularity matrix. The Laplacian modularity, like modularity, values the coarse one-way partition as zero for any *S* (see Supplementary Material, section 2.2). However, unlike modularity, it does not suffer from the traditional resolution limit^13^ if *S* is a local potential (see Supplementary Material, section 2.6).

An alternate interpretation of the Laplacian modularity matrix is that the *ij*^*th*^ term is the curvature of a link:

3 $$B(i,j)={d}^{i}(S(i,j)-\frac{1}{{d}^{i}}\sum _{k\in N(i)}S(k,j))+{d}^{j}(S(i,j)-\frac{1}{{d}^{j}}\sum _{k\in N(j)}S(i,k\mathrm{)).}$$

Here *d*^*i*^ is the degree of node *i* and *N*(*i*) indicates the immediate neighbors of node *i*. The terms in the parenthesis are the similarity of node *i* and *j* minus the average similarity over the local neighborhoods of nodes *i* and *j*, respectively (Note in the term $\frac{1}{{d}^{i}}{\sum }_{k\in N(i)}S(k,j)$, the sum is over the similarity between neighbors of node *i* and node *j*. This term equates to 1 for *S* = *A* only if all neighbors of node *i* are also connected to *j*. See Supplementary Material, section 2.3). That is, it measures the rate at which the connectivity of a link deviates from the average connectivity value over a local neighborhood. We show Fig. 3e to illustrate how the curvature expression varies based on the local configuration. The left column shows that the value of curvature for a link between two nodes *i* and *j*, each with degree 6, decreases as the number of common neighbors increase. A high value of curvature between two nodes implies a higher likelihood of the link to be a boundary link. Similarly, the right column shows the variation of curvature between two nodes *i* and *j* for the non-edges. Thus, each term in *B* encodes a curvature-like metric and collectively, a subgraph is a community for the Laplacian modularity if the internal connectivity is higher than the average connectivity calculated over the local neighborhood of nodes in the subgraph.

**Figure 4:**
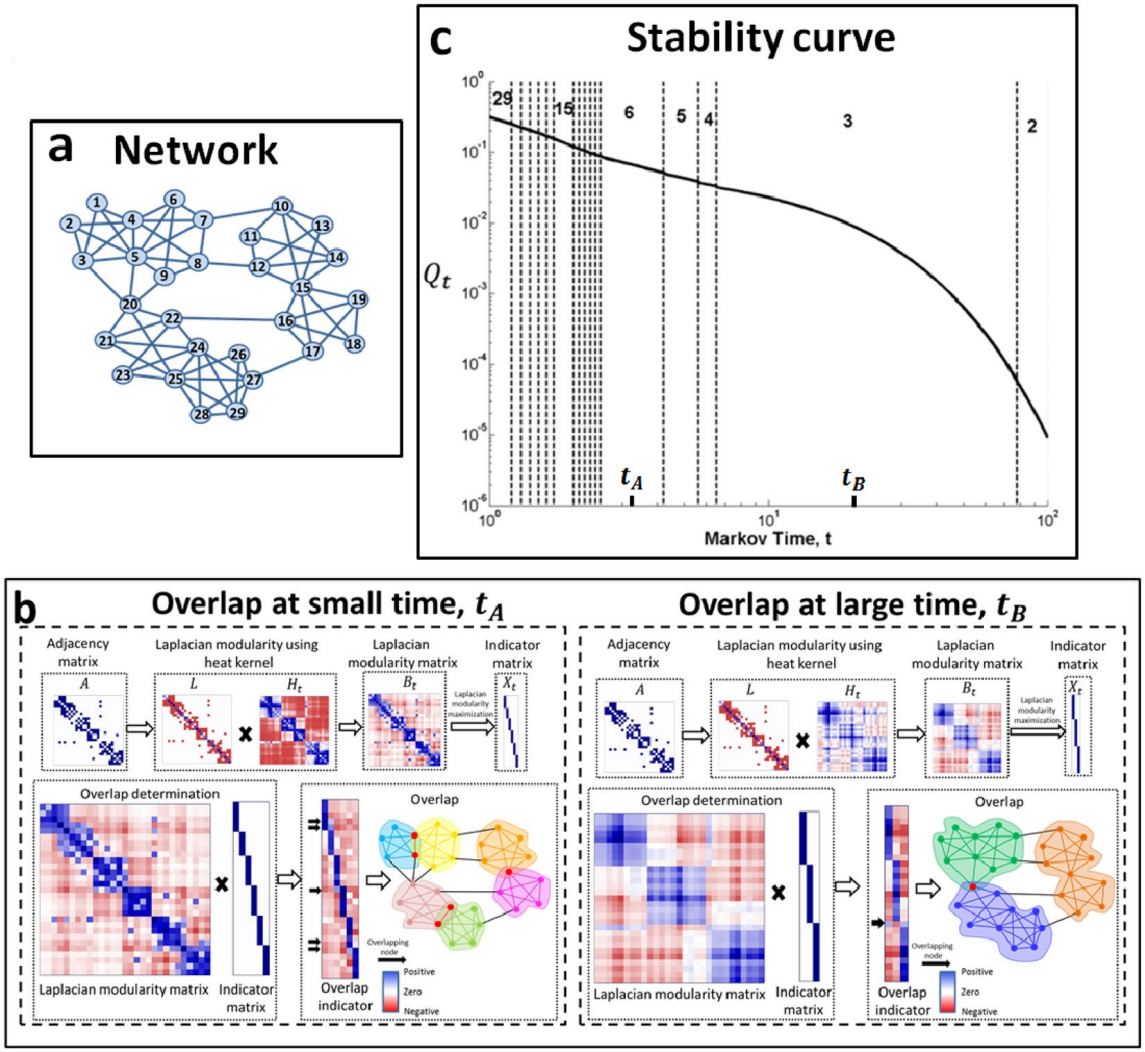
Overlapping and hierarchal communities using Laplacian modularity and the heat kernel. The heat kernel depends on a parameter *t* that controls the resolution of the communities (small *t* is fine, and large *t* is coarse). (**a**) Network of 29 nodes. (**b**) (Left) Six identified communities at a small time, *t*_*A*_ with overlapping nodes highlighted in red in the network representation. The rows and columns in the matrix representation follow a linear ordering indexed by node number. Blue matrix cells indicate positive values and red cells indicate negative values. (Right) Increasing the time shows three identified communities at large time, *t*_*B*_ with overlapping nodes in red. Observe the overlapping node at large time *t*_*B*_ is not overlapping at the small time *t*_*A*_, implying it has a weak connection with the individual yellow and blue communities identified at time *t*_*A*_, but a strong connection with them merged into one (green community at time *t*_*B*_), that is, overlap emerges with time *t*. (**c**) The stability curve derived using the heat kernel. The stability curve for the network shows two large persistent time-spans enclosed within dotted lines corresponding to the 3-way partition and 6-way partition.

Two prominent features of communities in real world networks are overlap, characterized as the possibility of nodes having multiple memberships in communities^14^, and hierarchy, representing the organization of communities over multiple scales^5^. Although there are abundant methods for finding overlapping^15,16^ and hierarchical^17,18^ community structure in network science individually, identifying relevant hierarchical communities in the presence of overlap remains an problem^9^. We resolve this problem by using the heat kernel of a network, *H*_*t*_, as the connectivity potential matrix *S* in order to holistically characterize overlapping and hierarchical communities. The heat kernel, *H*_*t*_, has a parametric dependence on the time scale, *t*, of a Markovian process on the network. Hence, it is intrinsically multiscale with a natural interpretation in terms of the size and resolution of the network communities under study (see Supplementary Material, section 3.1). We write the quality function where *S* is *H*_*t*_ as *Q*_*t*_. Although *Q*_*t*_ measures non-overlapping partitions, overlapping nodes can be identified by evaluating the nodal flux associated with each community boundary (see Fig. 4a,b and Methods). The parametric quantity *Q*_*t*_ also gives us a way to pick *t*. By viewing this quantity as a stability curve (see Fig. 4c and Methods)^18^, we can hierarchically organize network partitions, from fine to coarse as *t* increases. *Good* organizational levels of the network emerge as large regions in *t* with the same optimal community assignment (see Supplementary Material, section 3.4). Using this property, we develop a parameter free algorithm for Laplacian modularity optimization using the heat kernel to identify the most relevant partition of a network (see Methods).

**Figure 5:**
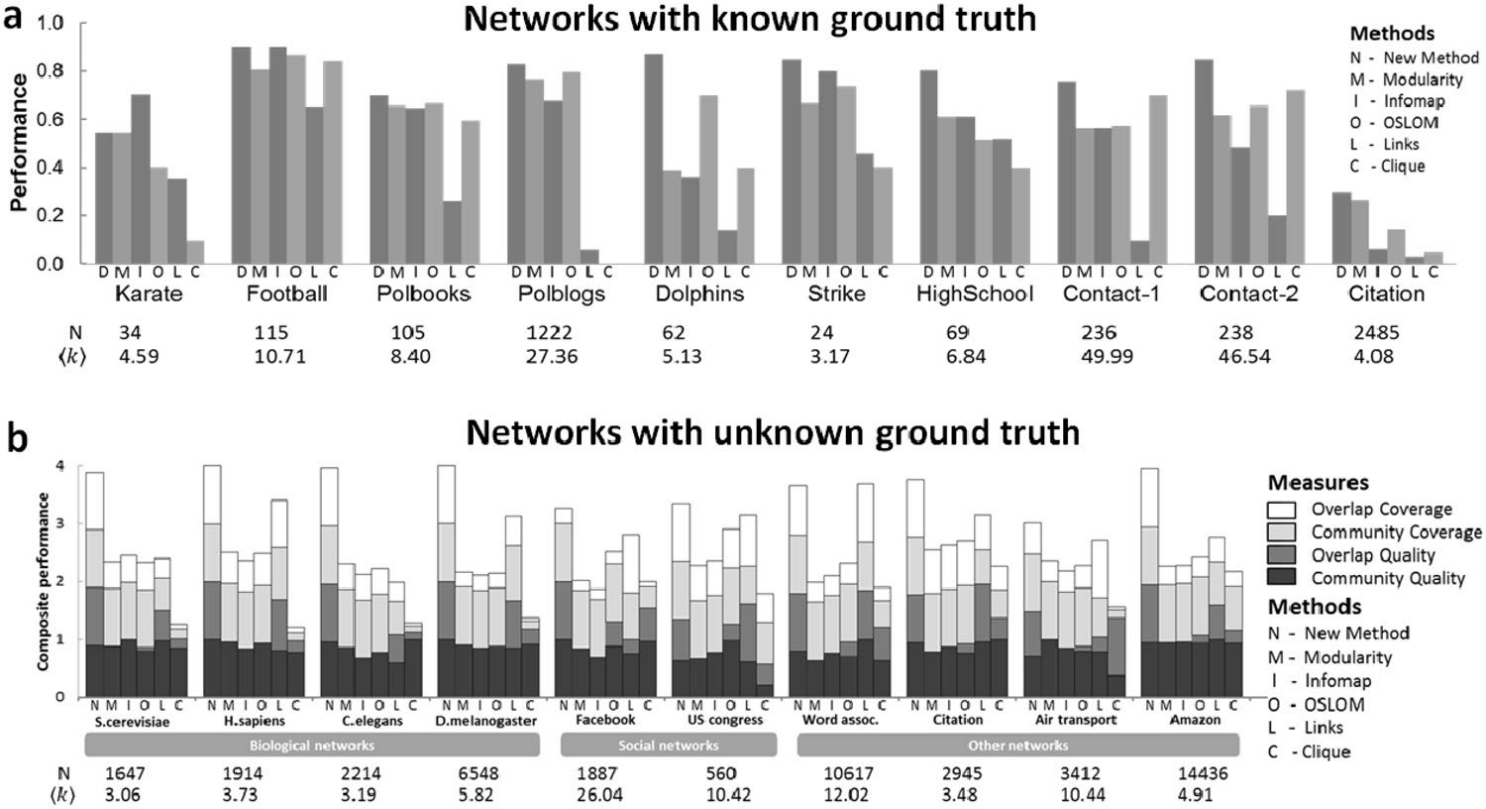
Performance of community detection methods on real-world networks. (**a**) Performance on ten networks with known community assignment measured with the omega index of identified communities against ground truth. (**b**) Composite performance based on metadata on ten networks with unknown community assignment. The tables below list the number of nodes, *N*, and the average node degree, $\langle k\rangle$ for each network.

We validate our approach using twenty networks of varying size and topology spanning diverse scientific domains. Of these, ten have known ground truth community assignments (see Supplementary Material, section 5 for details). On these networks, our Laplacian modularity optimization produces the most accurate communities (Fig. 5a) as measured in terms of the widely used omega index^19^ for 9 of the 10 networks when compared against five prominent community detection methods: modularity optimization^12,20^, Infomap^21^, OSLOM^22^, link communities^23^, and clique percolation^14^. For the networks with unknown community assignment, we follow a previously used approach^23^ to derive a composite performance metric for each method from the metadata. The composite performance metric is a normalized sum of four measures: community quality, overlap quality, community coverage, and overlap coverage. In Fig. 5b, we see that Laplacian modularity framework is the overall leader for all networks. Its performance is especially striking for biological networks that are expected to be incomplete. (See Supplementary Material, section 6 for additional details on these comparisons).

**Figure 6:**
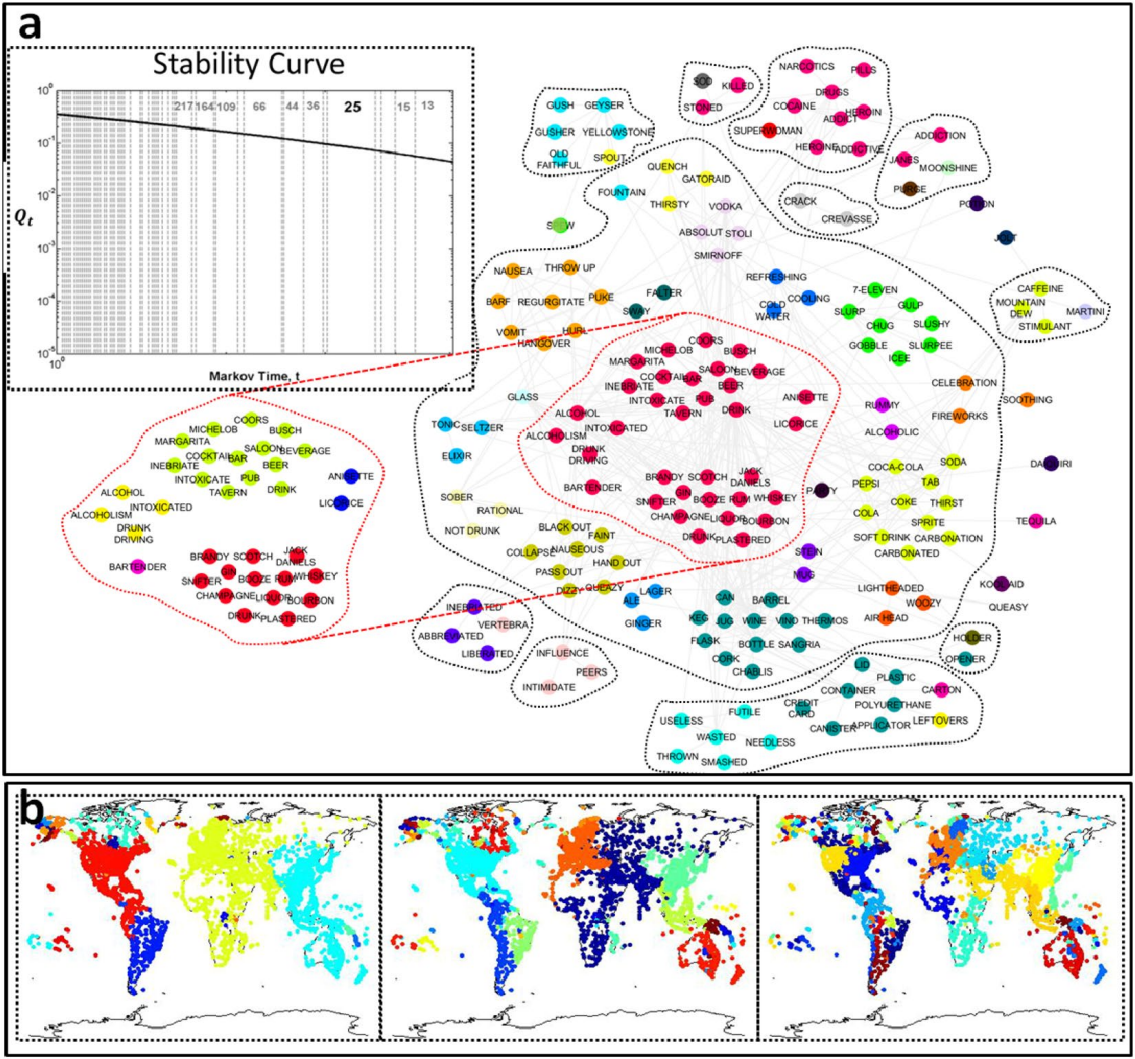
Hierarchical communities using Laplacian modularity. (**a**) Hierarchy in word association network. The stability curve (left) indicates that the network should be hierarchically organized into 25 and 66 communities. We also show the 164 and 217 communities information for deeper hierarchy. The subgraph shown is a community of the 25-way partition (first level). The dotted enclosed regions correspond to stable subcommunities in the 66-way partition (second level). The colors correspond to stable subcommunities in the 164-way partition (third level). Further subdivision of the red subcommunity in the 217-way partition is shown separately to the left (fourth level). (**b**) Hierarchical structure of air transportation network, divided into 28, 76 and 133 communities from left to right as identified by the stability curve identifies geopolitically significant regions at various size-scales.

In addition, our stability curve offers a natural way to interpret relevant hierarchical communities in the network. English words have associations at many different levels, and our method identifies four levels (Fig. 6a). For example, words associated with alcoholism and soft drinks appear as separate communities in the third level, and the alcoholism subcommunity is semantically subdivided in the fourth level. The stability curve of the air transportation network identifies partitions that correspond well to our natural concept of a community, i.e., the first level roughly identifies the large geopolitical regions of North America, South America, the Europe-Africa-Middle East region, and the Asia-Pacific region. The second level identifies finer regions including Europe (alone) and Australia, and the third level subdivides continents into small country-sized regions (Fig. 6b) while maintaining spatial proximity of overlapping communities (see Supplementary Material, section 6.4).

Finally, we do extensive comparisons on synthetic benchmarks including the stochastic block model and the LFR benchmark^24^, including further methodological comparisons against heat-kernel based analogues of Infomap and modularity. (See Supplementary Material, section 7.) These show our technique achieves better results in the LFR benchmark and slightly better results than the Hashimoto matrix^25^ in identifying the block-model components.

Our idea of identifying the community boundaries has its roots in the original Girvan-Newman algorithm that iteratively removes boundary links based on edge betweenness^7^. In comparison, optimizing the Laplacian modularity quality function reveals the boundary links all at once. The power of the Laplacian modularity framework also lies in the flexibility of discovering community structure given any metric of similarity, local or global, consistent with many ideas of cohesion (see Supplementary Material, section 8.2). These ideas have a strong connection to classical physics through the network analog of Gauss’s law. We believe that our approach using Gauss’s law to identify community boundaries, in concert with system-specific connectivity potentials, holds great promise in improving our understanding of the modular nature of complex networks.

More generally, our research establishes a new and complementary direction in the space of community detection algorithms based on looking for boundaries using curvature and network analogs of Gauss’s law. In comparison with modularity-based methods, it provides substantial additional flexibility and seeks partitions based on how the boundaries of the communities appear in the data given connectivity potentials; and we get to re-use algorithmic innovations in the space of modularity clustering because our final matrix shares the same type of optimization objective (see Supplementary Material, section 2.5). More broadly, in comparison with a number of techniques based on diffusions or flows^26^, our work can be interpreted as a midpoint between modularity-style statistical deviance techniques and diffusion-style conductance techniques in that Laplacian modularity combines continuous diffusion, when setting the connectivity potential via the heat-kernel, with a boundary detection scheme. In the future this approach could in principle be combined with connectivity information in *S* based on network flows among various regions of the graph to produce further hybrid methods^27^ or with spectral algorithms based on eigenvectors of the Laplacian modularity matrix that might give robust interpretations to various heuristic decisions that many practitioners make in large scale graph experiments^28^.

We have illustrated the Laplacian modularity under the paradigm of heat diffusion in order to systematically uncover overlapping communities organized at relevant hierarchical scales of a network. Our framework extends to other notions of similarity that generate the connectivity potentials. We also investigate multi-resolution Laplacians, cosine similarities, and adjacency matrices (see Supplementary Material, section 8). Our method scales to networks with millions of nodes when the connectivity potential is sparse. The Laplacian modularity quality function is further extensible to networks with directed, weighted, and signed links (see Supplementary Material, sections 4.1, 4.2, 4.3), applicable to time-dependent multiplex or multislice networks (see Supplementary Material, section 4.4), and hence, capable of identifying community structure in a broad class of networks. Indeed, because of the focus on boundaries, our method is able to seamlessly generalize to using boundaries in terms of network motifs rather than edges^29^.

## Methods

### Evaluating connectivity flux through a boundary link

According to classical electrodynamics, the electric field at a point in space is the gradient of the electrostatic potential at the corresponding point. Given the connectivity potentials between all pairs of nodes, the connectivity flux for a node *i* through a link *E*_*b*_ is then the symmetric difference of node *i*’s connectivity potentials with respect to the two nodes constituting the link, *E*_*b*_, say *m* and *n* (Fig. 6c, Top) (The connectivity field is conservative and has curl zero along any path on the network. See Supplementary Material, section 2.3):

4 $${S}^{i}({E}_{b})=(S(i,m)+S(m,i))-(S(i,n)+S(n,i\mathrm{)).}$$

Here, *m* refers to an internal node and *n* to an external node. As the flux through a surface is the area integral of the electric field, the net connectivity flux of node *i* through a boundary is the sum of the connectivity flux through all links constituting the subgraph boundary, *E*_*B*_, i.e., ${\sum }_{b\in B}{S}^{i}({E}_{b})$ (Fig. 2c, Bottom).

### Internal and external boundary cohesion

Consider the subgraph induced by a subset of nodes *C* and let *V*, *E* be the set of all nodes and links in the network, respectively. Let *E*_*B*_ be the boundary links of this subgraph and *C*_*B*_ be the multiset of nodes induced by the boundary links:

5 $${C}_{B}=\mathop{\cup }\limits_{\begin{array}{c}(m,n)\in E\\ m\in C\\ n\notin C\end{array}}\{\{m\}\}\cup \{\{n\mathrm{\}\}.}$$

Then the multiset of internal nodes is represented as ${C}_{in}=\{\{m|m\in C\,{\rm{and}}\,m\in {C}_{B}\}\}$, and the multiset of external nodes is represented as ${C}_{out}=\{\{n|n\notin C\,{\rm{and}}\,n\in {C}_{B}\}\}$. The cohesion of node *i* with respect to a general multiset ${\mathscr{T}}$ is:

6 $${S}^{i}({\mathscr{T}}\,)=\sum _{j\in {\mathscr{T}}}(S(j,i)+S(i,j)),$$

where the summation includes all the repetitions in the multiset. The internal boundary cohesion of node *i* with respect to *C*_*B*_${C}_{B}$ is then ${S}^{i}({C}_{in})$ and the external boundary cohesion is ${S}^{i}({C}_{out})$. This decomposition enables us to write the connectivity flux of node *i* through the boundary as ${\sum }_{b\in B}{S}^{i}({E}_{b})={S}^{i}({C}_{in})-{S}^{i}({C}_{out})$ (Fig. 2d).

### Identifying overlap

Overlapping nodes are simply nodes with net positive connectivity flux to multiple communities for the given connectivity potentials *S*. Mathematically, the positive terms in the matrix ${X}^{T}L(S+{S}^{T})$ identify overlapping nodes, where *X* is a 0–1 indicator matrix for the hard partition of dimensions |*V*| × *K* where |*V*| is the number of nodes in the network and *K* the number of communities (see Supplementary Material, section 2.4 and Fig. 4b). This definition can be formally interpreted using the strong and weak definition of communities given in the Supplementary Material, section 2.4.

### Determining hierarchy

Hierarchy results from varying *t* in the heat kernel, *H*_*t*_ used as the connectivity potential, *S*. Note that the communities at two different instants of time *t* don’t always comprise each other; and thus using the heat kernel as a metric of similarity identifies a *soft* hierarchy. The element *H*_*t*_ (*i*, *j*) in the heat kernel represents the probability of a random walker to move from node *i* to node *j* after a walk of length $\ell$ where $\ell$ is determined by a Poisson process with mean *t*. It suffices to only consider the term $L{H}_{t}$ for $S={H}_{t}$ in Eq. 2 because $L{H}_{t}$ is symmetric, unbiased and has an intuitive diffusion interpretation (see Supplementary Material, section 3.2). We represent $L{H}_{t}$ as ${B}_{t}$ in this discussion. Substituting ${H}_{t}={e}^{-t{\mathbb{L}}}$ in ${B}_{t}$ results in $D{\mathbb{L}}{e}^{-t{\mathbb{L}}}$, where ${\mathbb{L}}$ is the random walk Laplacian which is physically interpreted as the negative rate of diffusion $(\,\,-\,\frac{d{H}_{t}}{dt})$ scaled by *D*. It follows from principles of diffusion that the partition which maximizes ${Q}_{t}$ (the trace of the clustered matrix, ${X}^{T}{B}_{t}X$) minimizes the net diffusive tendency within subgraphs. The heat diffusion may reach a temporary local equilibrium at time $t$ before it proceeds to the global equilibrium ($t=\infty$). Intuitively, a partition optimal over a long time span corresponds to a stable temporary equilibrium. Using this intuition in conjunction with the monotonicity of ${Q}_{t}$, we define a stability curve *r*(*t*) to be the maximum value of the Laplacian modularity over the space of all partitions *X*, i.e,

7 $$r(t)={{\rm{\max }}}_{X}{\rm{trace}}(\frac{1}{\mathrm{2|}E|}{X}^{T}{B}_{t}X),$$

where the trivial constant 2|*E*| ensures that the measure lies in [0, 1] and |*E*| is number of links. The hierarchical community organization of the network can be explored using this stability curve.

### Laplacian Modularity Optimization

We use the heat kernel *H*_*t*_ as *S* in all our experiments and use the Louvain method^20^ to optimize Laplacian modularity *Q*_*t*_ at each time *t* (see Supplementary Material, section 2.5 for pseudo code) (Thus, our method is not a spectral method based on relaxing to real-valued assignments or eigenvectors.) However, only the partition with maximal persistence over a logarithmic time range is picked for a fair comparison to the other methods (see Supplementary Material, section 3.5). In order to discern overlap in this optimal partition, we first calculate the fuzzy overlap membership of each node to all subgraphs at discrete time values in the persistent basin. This corresponds to entries of the matrix ${X}_{o}^{T}{B}_{t}$, where ${X}_{o}$ is the overlap indicator matrix (see Supplementary Material, section 3.5). We then introduce a threshold parameter *ε* that converts fuzzy overlap membership over the time range into a hard overlap. The choice of *ε* is guided by the intuition that *ε* = 1 outputs a hard partition (no overlap), and *ε* = 0 identifies small structural overlaps. The latter is especially relevant for networks with incomplete information or missing links. We set *ε* = 1 for networks with known communities because the ground-truth is a hard partition and *ε* = 0 for networks with unknown community assignment where we expect pervasive overlap.

## Electronic supplementary material

Supplementary Material
