# Supplementary material for: Gauss’s law for networks directly reveals community boundaries
Source: Sci Rep. 2018 Aug 9;8:11909. doi: 10.1038/s41598-018-30401-0 (PMC6085300; doi:10.1038/s41598-018-30401-0)
Supplement: Supplementary file 1 — Supplementary Material [file 41598_2018_30401_MOESM1_ESM.pdf]

Gauss's law for networks directly reveals community boundaries-Supplementary Material

Ayan Sinha[1*,3], David F. Gleich[2], Karthik Ramani[1]

[1]School of Mechanical Engineering, Purdue University,IN 47907, USA
[2]Department of Computer Science, Purdue University, IN 47907, USA
[3]Currently at Magic Leap, CA 94089, USA

*Correspondence should be addressed to: sinhayan@gmail.com

Contents

1 Introduction **4**

2 The formulation, properties and optimization of Laplacian modularity **4**
2.1 Definitions of a community according to Laplacian modularity 4
2.2 Laplacian modularity quality function 5
2.3 Connecting Gauss's law and our Gauss's law for networks 14
2.4 Identifying overlapping nodes 18
2.5 Optimization of Laplacian modularity 20
2.6 Analysing the resolution limit of Laplacian modularity 21
2.7 Multi-resolution extension of Laplacian modularity 25
2.8 Resistor network interpretation of Laplacian modularity 26

3 Laplacian modularity using heat kernel **28**
3.1 Markov chains and the heat kernel 28
3.2 Diffusion based Laplacian modularity (DLM) 31
3.3 Identifying overlapping nodes using DLM 35
3.4 Identifying hierarchy using DLM 36
3.5 Automatic community identification 40
3.6 Analysing the resolution limit for DLM 44

4 Extending Laplacian modularity to other network types **47**
4.1 Weighted Networks 47
4.2 Directed Networks 48
4.3 Signed networks 50
4.4 Multislice Networks 51

5 Network datasets with known community assignment **53**
5.1 Other community detection methods 54
5.2 Results on networks with known ground truth 56

6 Network datasets with unknown community assignment **61**
6.1 Measures 61
6.2 Biological networks 64
6.3 Social networks 65
6.4 Other networks 68

7 Results on benchmarks **74**
7.1 Comparison with multiscale modularity and multiscale Infomap 74
7.2 Comparison with spectral methods 76
7.3 Comparison with community detection methods 79

8 Laplacian modularity applied to large networks and for alternate measures of connectivity potentials **82**
8.1 Laplacian modularity applied to large networks 82
8.2 Laplacian modularity for alternate definitions of connectivity potentials 84

List of Figures

1 Cohesion to subgraph boundary 8
2 Gauss's law on a 2D domain 13
3 Partitioning a 2D domain using Gauss's law 15
4 Connectivity potentials for Gauss's law for networks 16
5 Connectivity distribution for Gauss's law for networks. 17
6 Laplacian modularity matrix 19
7 Resolution limit 21
8 Resistor network interpretation of Laplacian modularity 27
9 Comparing stability curves 37
10 Comparing stability curves of networks with communities and random networks 40
11 Community detection using diffusion based Laplacian modularity 43
12 Comparing diffusion dynamics and rate of diffusion dynamics 45
13 Laplacian modularity for a directed network 48

14 Laplacian modularity for a multislice network 52
15 Results of other community detection methods on synthetic graph 53
16 Diffusion based Laplacian modularity applied to karate network 54
17 Diffusion based Laplacian modularity applied to football network 55
18 Diffusion based Laplacian modularity applied to political books network 57
19 Diffusion based Laplacian modularity applied to dolphin network 58
20 Diffusion based Laplacian modularity applied to strike network 58
21 Diffusion based Laplacian modularity applied to highschool network 59
22 Diffusion based Laplacian modularity applied to contact-1 network 60
23 Diffusion based Laplacian modularity applied to contact-2 network 61
24 Comparison with Neo K-means on real-world networks. 62
25 Diffusion based Laplacian modularity applied to S.cerevisiae network 63
26 Diffusion based Laplacian modularity applied to H.sapiens network 63
27 Diffusion based Laplacian modularity applied to C.elegans network 64
28 Diffusion based Laplacian modularity applied to D.melanogaster network . . . 64
29 Split-betweenness evaluation 66
30 Diffusion based Laplacian modularity applied to Facebook network 67
31 Diffusion based Laplacian modularity applied to cosponsorship network 68
32 Diffusion based Laplacian modularity applied to word association network . . . 69
33 Diffusion based Laplacian modularity applied to citation network 70
34 Diffusion based Laplacian modularity applied to air transport network 71
35 Diffusion based Laplacian modularity applied to Amazon co-purchasing network 72
36 Overlapping landscape strength over time 73
37 Performance of multiscale community detection methods on real-world networks with known ground truth. 75
38 Performance of multiscale community detection methods on undirected and unweighted LFR benchmark graphs without overlapping communities. 75
39 Performance of multiscale community detection methods on hierarchical LFR benchmark graphs (undirected, unweighted, without overlapping communities). 76
40 Eigenvector corresponding to largest eigenvalue for the operator $LS + SL$. . . 77
41 Comparison with spectral community detection using Hashimoto operator on real-world networks. 78
42 Performance of multiscale community detection methods on undirected and unweighted LFR benchmark graphs without overlapping communities. 79
43 Performance of extremely sparse graphs created by the stochastic block model. 80
44 Performance of community detection methods on undirected and unweighted LFR benchmark graphs without overlapping communities. 81
45 Performance of community detection methods on hierarchical LFR benchmark graphs (undirected, unweighted, without overlapping communities). 81
46 Performance on undirected and unweighted LFR benchmark with overlapping communities. 82

47 Diffusion based Laplacian modularity applied to large networks 83
48 Laplacian modularity for alternate definitions of similarity 83
49 Stability curves for multi-resolution Laplacian modularity 87

1 Introduction

In this document we discuss details of our approach which we alluded to in the main manuscript. In section 2 we discuss features of the Laplacian modularity framework. In section 3 we discuss details of the Laplacian modularity using the heat kernel. In section 4, we extend our method to general network types. We discuss our results on networks with known ground truth in section 5, and provide details of networks with unknown community assignment in section 6. In section 7 we compare our method to alternate community detection methods on benchmarks, and finally we implement the Laplacian modularity on some large networks in section 8.

2 The formulation, properties and optimization of Laplacian modularity

In this section we discuss details of the Laplacian modularity quality function. Specifically, we provide a formal definition of communities from the purview of community boundaries, prove key properties of the Laplacian modularity, discuss techniques to optimize the quality function and reveal overlap using the Laplacian modularity matrix, examine the resolution limit of our approach, and provide a multi-resolution extension. We also provide an interpretation of the Laplacian modularity using resistor networks.

2.1 Definitions of a community according to Laplacian modularity

Although there is no consensus, a common abstraction is that communities in networks are groups of tightly connected nodes (dense intra-connections) and weakly connected to each other (sparse inter-connections). One intuitive formalization of a community in terms of direct connections is the LS-set, wherein each node has more internal connections than with the rest of the network (*LS69*). For a network with adjacency matrix, A where $A(i,j) = 1$ if there is a direct connection between nodes i and j and 0 otherwise, a subgraph C is a LS-set if $d^i(C) > d^i(\bar{C}), \forall i \in C$. $d^i(C) = \sum_{j \in C} A(i,j)$ is the internal degree of a node i, and $d^i(\bar{C}) = \sum_{j \in \bar{C}} A(i,j)$ is its external degree to the rest of the network, $\bar{C}$ (*RCC$^+$04*). An alternate approach is to construct a hierarchical clustering tree by agglomerating a node-to-node connectivity matrix, S, and subsequently cut branches off the dendrogram to output a network partition (*HTF01*). However, there exist several partitions of a network into LS-sets or cutoff values in hierarchical clustering. Community detection algorithms aim to automatically determine the most relevant partition or equivalently the number of communities in a network,

a characteristic which separates community detection from traditional graph partitioning algorithms (*For10*). Hence, the algorithmic detection of an optimal partition is most popularly premised on comparing the internal degree of a node to its expectation under a null model, $d^i(C) > \langle d^i(C) \rangle$, as in the modularity quality function (*New06b*). Different from all other approaches, we define communities in terms of the two components of the connectivity flux guided by the rationale that a community member is more cohesive to fellow members inside the community boundary than those outside.

Strong Community: Let the set of all nodes be V. The subgraph $C \in V$ is a community in a strong sense if:

$$S^i(C_{in}) > S^i(C_{out}), \forall i \in C. \tag{1}$$

Here $S^i(C_{in})$ and $S^i(C_{out})$ are the internal and external boundary cohesion, respectively, described in the main manuscript. In a strong community, the internal boundary cohesion of each node is greater than its external value for subgraph, C, similar to the internal degree of each node is greater than its external degree in an LS-set.

Weak Community: The subgraph $C \in V$ is a community in a weak sense if:

$$S_{in}(C) > S_{out}(C). \tag{2}$$

$S_{in}(C), S_{out}(C)$ represent sum of $S^i(C_{in}), S^i(C_{out})$ respectively over all nodes in C. In a weak community, the sum of internal boundary cohesion over all nodes in C is greater than the sum of external boundary cohesion, for subgraph, C, in analogy with the sum of internal degrees exceeds the sum of external degrees of nodes in a weak community (*RCC$^+$04*). It is apparent that a community in the strong sense is also a community in a weak sense, whereas the converse is not true. As in a LS-set, these definitions of a community account for intra-subgraph and inter-subgraph cohesion. However, unlike LS-sets, they circumvent bias to subgraph size because for every internal boundary node there is an external counterpart. Also, our definition of communities in the strong and weak sense is analogous to strong and weak definitions of a community proposed in (*RCC$^+$04*), formulated from the purview of nodes on community boundaries instead of all internal and external nodes. Next, we discuss properties of the Laplacian modularity quality function.

2.2 Laplacian modularity quality function

In this subsection we establish our Gauss's law for networks by relating the connectivity flux flowing through the boundary links to the connectivity density within modules. We derive the Laplacian modularity quality function starting from the two disjoint components of the connectivity flux, i.e., the internal and external boundary cohesion.

2.2.1 Notations and terminology

We first introduce the notations and terminology that will be used for deriving the Laplacian modularity quality function. Let V be the set of all nodes in the network and the set of boundary

links for subgraph C be E_B such that the node pair $[m, n]$ associated with each link satisfy $m \in C, n \notin C$. Note m is an internal node ($m \in C_{in}$) and n is an external node ($n \in C_{out}$). As described in the main manuscript, the mutual connectivity of a node i, $S^i(\mathcal{T})$, to a multiset of nodes, $\mathcal{T}$, based on connectivity potential matrix, S is assessed as:

$$S^i(\mathcal{T}) = \sum_{j \in \mathcal{T}} \big(S(j,i) + S(i,j)\big). \tag{3}$$

In matrix form we have the representation:

$$S(\mathcal{T}) = S^T \mathcal{V} + S\mathcal{V}. \tag{4}$$

$\mathcal{V}$ is the membership vector where the element $\mathcal{V}^i$ details the cardinality of node i's occurrence in multiset $\mathcal{T}$. $S^T\mathcal{V}$ models the connectivity of nodes in the network to multiset $\mathcal{T}$, whereas $S\mathcal{V}$ models the reciprocal connectivity of nodes in multiset $\mathcal{T}$ to nodes in the network. A node participating in x boundary links (internal or external) is seeded x times, so as to maintain the total cardinality of the two membership vectors. Such a node inherently mediates greater control over the measure of cohesion relative to a node participating in one or no boundary link, akin to the personalization vector in PageRank (*JW03*). The two components of the connectivity flux are then represented as:

$$S^i(C_{in}) = S^T \mathcal{V}_{in} + S\mathcal{V}_{in} = \sum_{E_B} \big(S(m,i) + S(i,m)\big) \tag{5a}$$

$$S^i(C_{out}) = S^T \mathcal{V}_{out} + S\mathcal{V}_{out} = \sum_{E_B} \big(S(n,i) + S(i,n)\big). \tag{5b}$$

$\mathcal{V}_{in}, \mathcal{V}_{out}$ are the membership vectors where the ith element details the cardinality of node i's occurrence in multisets C_{in} and C_{out}, respectively. The similarity of node i to the multiset C_{in} or C_{out} is equal to the sum of connectivity potentials of node i to induced internal boundary nodes and external boundary nodes, respectively. Note that we use the term similarity and connectivity potential interchangeably in this entire document. The authors in (*FCF*) motivate a sophisticated measure of cohesion based on triangle density in order to determine communities. In contrast to their definition, our measure of cohesion is generally applicable to any similarity matrix, S encoding connectivity potentials.

We realize that some researchers in network science adopt different notations. Hence, we define some key measures in this manuscript using alternate notations for comprehensibility to a wider network science audience:

- The mutual connectivity of a node i to a multiset of nodes, $\mathcal{T}$ is represented as:

  $$S^i(\mathcal{T}) = e_i^T (S + S^T)\mathcal{V}. \tag{5c}$$

  Here e_i is an indicator vector for node i and $\mathcal{V}$ is the multiset indicator vector or the membership vector.

- The mutual connectivity of nodes in set C to a multiset of nodes, $\mathcal{T}$ written as $S_{\mathcal{T}}(C)$, can be equivalently represented as:

$$S_{\mathcal{T}}(C) = e_C^T(S + S^T)\mathcal{V}. \tag{5d}$$

Here e_C is an indicator vector for nodes in C.

- The connectivity flux through a boundary link E_b for node i, $S^i(E_b)$ is represented as:

$$S^i(E_b) = \sigma_b e_i^T(S + S^T)\mathcal{N}e_b. \tag{5e}$$

Here $\mathcal{N}$ is the node-link incidence matrix, e_b is a link indicator vector and σ_b defines the sign of outward orientation for the boundary link.

- The Laplacian modularity quality function for a partition with indicator matrix X is represented as:

$$Q(X) = tr\big(X^T(LS + SL)X\big). \tag{5f}$$

Here $tr(Z)$ indicates the trace of the matrix Z.

Figure 1 A highlights the internal and external boundary nodes of subgraph C^1 in a synthetic network of 16 nodes comprising of 3 cliques. The boundary links (green edges) induce the multiset ([6,6,8,7]) and set ([2,5,14,13]) to be the internal (blue) and external (red) boundary nodes, respectively. Figure 1 B,C graphically illustrates the calculation of internal and external boundary cohesion for the subgraph C^1, by setting $S = A + I$. I is an identity matrix which assigns self-similarity to each node. The sum of all elements in vectors $\mathcal{V}_{in}$ as well as $\mathcal{V}_{out}$ is equal to the total number of boundary links to the subgraph, i.e., four for C^1, and the multiplicity of node 6 is two in C^1_{in} as it connects to two boundary links.

If a subgraph, C is a community in the weak sense, we have $q(C) = S_{in}(C) - S_{out}(C) > 0$, An additive quality function over subgraphs in partition $\mathcal{P}$ derived from the weak definition of a community is then given by:

$$\begin{aligned} Q &= \sum_{C\in\mathcal{P}} q(C) \\ &= \sum_{C\in\mathcal{P}} (S_{in}(C) - S_{out}(C)) \\ &= \sum_{C\in\mathcal{P}}\sum_{i\in C} (S^i(C_{in}) - S^i(C_{out})). \end{aligned} \tag{6}$$

Here C is the subgraph, $S^i(C_{in})$ and $S^i(C_{out})$ are the internal and external boundary cohesion, respectively. Our main result is that optimizing quality function Q in equation 6 is equivalent to optimizing the function:

$$Q = \sum_{i,j}[LS + SL]\delta(i,j) = \sum_{i,j}[(D - A)S + S(D - A)]\delta(i,j). \tag{7}$$

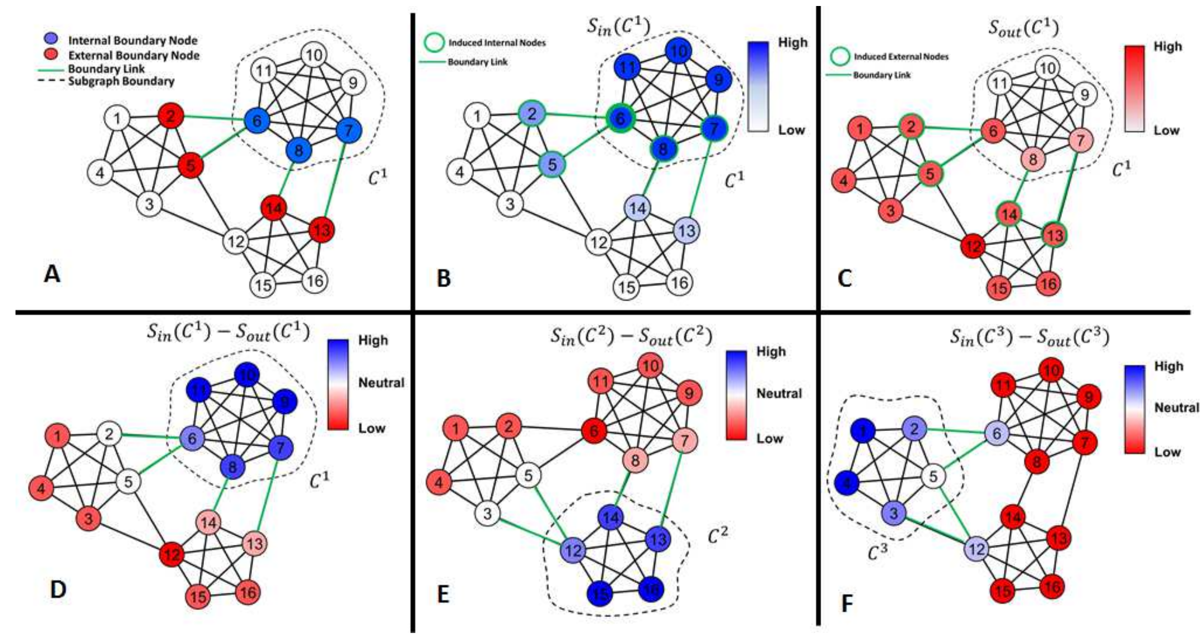

Figure 1: Cohesion to subgraph boundary. *A:Internal (blue) and external (red) boundary nodes induced by boundary links (green) by dotted subgraph boundary of C^1. B:Cohesion of nodes in network to internal boundary nodes of C^1. Higher values of internal boundary cohesion are displayed as darker shades of blue. C: Cohesion of nodes in network to external boundary nodes of C^1. Higher values of external boundary cohesion are displayed as darker shades of red. D: Difference of internal and external boundary cohesion due to C^1. E:Difference of internal and external boundary cohesion due to C^2. F:Difference of internal and external boundary cohesion due to C^3. Color legends for $S_{in}(C) - S_{out}(C)$ values are displayed to the right in subplots D,E,F.*

Here Q is the Laplacian modularity quality function, S is the node-to-node connectivity matrix, L is the Laplacian formulated as $L = D - A$, D is a diagonal matrix of node degrees, A is the adjacency matrix, and $\delta(i, j)$ is a delta function wherein $\delta(i, j) = 1$ if both i and j belong the same community and 0 otherwise. We first prove our Gauss's law for networks, i.e.:

$$\sum_{C \in \mathcal{P}} \sum_{i \in C} (S^i(C_{in}) - S^i(C_{out})) = \sum_{i,j} [(D - A)S + S(D - A)]\delta(i, j). \tag{8}$$

Our second result in this subsection is that our quality function is an unbiased measure at all resolutions, or that the coarse one-way partition evaluates to zero. Formally, we prove that:

$$Q^{ass} + Q^{diss} = 0. \tag{9}$$

Here Q_{ass} and Q_{diss} are the associative and dissociative quality functions, respectively. In the main manuscript we formulate an additive quality function, Q. The expectation is that the best

assignment maximizes the difference of $S^C(C_{in})$ and $S^C(C_{out})$ over all subgraphs, $C \in \mathcal{P}$. The derived quality function is the difference of internal and external cohesion of nodes with respect to its own subgraph, and hence, represents association within subgraphs and we also denote Q as Q_{ass}:

$$Q^{ass} = \sum_{C \in \mathcal{P}} (S_{in}(C) - S_{out}(C)). \tag{10}$$

An alternate strategy to find the best assignment is to minimize the difference of $\sum_{C^* \in \mathcal{P}} S_{in}(C^*)$ and $\sum_{C^* \in \mathcal{P}} S_{out}(C^*)$ for the set of all subgraphs C^*. Here, the sum over C^* represents the sum over all subgraphs in $\mathcal{P}$ but C. This is the difference of internal and external boundary cohesion of nodes with respect to all but its own subgraph, and hence, represents the net disassociation between C and set of C^*. The overall dissociation between all sets of subgraphs, i.e., between all C^k and C^l such that $k \neq l$ is then given by:

$$Q^{diss} = \sum_{C \in \mathcal{P}} \sum_{C^*} (S_{in}(C^*) - S_{out}(C^*)). \tag{11}$$

As this quality function represents the disassociation between subgraphs and we denote it as Q^{diss}.

2.2.2 Establishing Gauss's law for networks and unbiasedness of Laplacian modularity

Let us denote the matrix $[(D-A)S]$ as B_1 and the matrix $[S(D-A)]$ as B_2. Before proceeding to the proof of equation 8, which is our Gauss's law for networks, and equation 9, which states that the Laplacian modularity quality function is an unbiased measure, we first state few properties of the matrices B_1 and B_2.

Property 1. *For any node i in subgraph C:*

$$(S^i(C_{in}) - S^i(C_{out})) = \sum_{j \in C} (B_1(j,i) + B_2(i,j)). \tag{12}$$

Here $B_1(i,j)$ indicates the $(i,j)^{th}$ element of the matrix $[(D-A)S]$ and $B_2(i,j)$ indicates the $(i,j)^{th}$ element of the matrix $[S(D-A)]$.

Proof. $\sum_{j \in C} B_1(j,i)$ sums over the elements of the ith column of the matrix product $[(D-A)S]$ for all j belonging to C. Similarly, $\sum_{j \in C} B_2(i,j)$ sums over the elements of the ith row of the matrix product $[S(D-A)]$ for all j belonging to C. Without loss of generality, assume that the subgraph C is a set of k nodes. Using property of matrix multiplication, the $(j,i)^{th}$ element of B_1 corresponds to the vector dot product of jth row of $(D-A)$ and i^{th} column of S. Also, $(i,j)^{th}$ element of B_2 corresponds to the vector dot product of ith row of S and j^{th} column of $(D-A)$. It is easy to verify that $\sum_{j \in C} B_1(j,i)$ is of the form $\sum_m c^1_m S(m,i), m \in V$ where $[c^1_1, c^1_2, \cdots c^1_m \cdots c^1_{|V|}]$ is a $|V| \times 1$ vector of coefficients corresponding to B_1. Note that

it only contains terms corresponding to the ith column of S. Similarly, $\sum_{j \in C} B_2(i,j)$ is of the form $\sum_m c_m^2 S(i,m), m \in V$ where $[c_1^2, c_2^2, \cdots c_m^2 \cdots c_{|V|}^2]$ is a $|V| \times 1$ vector of coefficients corresponding to B_2. It only contains terms corresponding to the ith row of S. Furthermore, the coefficients c_m^1 are given by the column sum of the $k \times |V|$ matrix $(D-A)_C^r$ where $(D-A)_C^r$ represents the k rows corresponding to nodes in C and the coefficients c_m^2 are given by the row sum of the $|V| \times k$ matrix $(D-A)_C^c$ where $(D-A)_C^c$ represents the k columns corresponding to nodes in C. The column and row sums of entire matrix $(D-A)$ is 0 by construction. The following hold for column sums of the matrix $(D-A)_C^r$:

- The mth column sum is positive if and only if $m \in C$ and node m has an edge connecting to at least one node $n \notin C$. The coefficient c_m^1 is then equal to number of links connecting $m \in C$ to $n \notin C$. This follows from observing that the only positive entries in $(D-A)_C^r$ correspond to the diagonal entries in matrix $(D-A)$ and the column sum of entire matrix $(D-A)$ is 0.

- The mth column sum is 0 if $m \in C$ and node m has no edges connecting to nodes $n \notin C$ (the positive entry exactly cancels out all negative entries), or $m \notin C$ and has no edges connecting to nodes $n \in C$ (all entries are 0).

- The nth column sum is negative if and only if $n \notin C$ and node n has an edge connecting to at least one node $m \in C$. The coefficient c_n^1 is then equal to number of links connecting $n \notin C$ to $m \in C$. This is true because the positive diagonal entries do not appear in the columns for $n \notin C$ in matrix $(D-A)_C^r$, and hence, all entries are negative and correspond to edges connecting it to nodes $m \in C$.

Analogously, the following hold for row sums of the matrix $(D-A)_C^c$:

- The mth row sum is positive if and only if $m \in C$ and node m has an edge connecting to at least one node $n \notin C$. The coefficient c_m^2 is then equal to number of links connecting $m \in C$ to $n \notin C$. This is true as the only positive entries in $(D-A)_C^c$ correspond to the diagonal entries in matrix $(D-A)$ and the row sum of entire matrix $(D-A)$ is 0.

- The mth row sum is 0 if $m \in C$ and node m has no edges connecting to nodes $n \notin C$ (the positive entry exactly cancels out all negative entries), or $m \notin C$ and has no edges connecting to nodes $n \in C$ (all entries are 0).

- The nth row sum is negative if and only if $n \notin C$ and node n has an edge connecting to at least one node $m \in C$. The coefficient c_n^1 is then equal to number of links connecting $n \notin C$ to $m \in C$. This is true because the positive diagonal entries do not appear in the columns for $n \notin C$ in matrix $(D-A)_C^c$, and hence, all entries are negative and correspond to edges connecting it to nodes $m \in C$.

Now consider the term $(S^i(C_{in}) - S^i(C_{out}))$ on LHS in equation 12. By definition:

$$S^i(C_{in}) = \sum_{E_B} (S(m,i) + S(i,m)). \tag{13a}$$

$$S^i(C_{out}) = \sum_{E_B} (S(n,i) + S(i,n)). \tag{13b}$$

Here E_B is the set of boundary or interfacing links for subgraph C such that the node pair $[m,n]$ associated with each link satisfy $m \in C, n \notin C$. $(S^i(C_{in}) - S^i(C_{out}))$ can be rewritten as $\sum_{m \in V}(\tilde{c}^1_m S(m,i) + \tilde{c}^2_m S(i,m)), m \in V$ where $\tilde{c}^1_m, \tilde{c}^2_m$ are positive or negative depending on $m \in C$ or $m \notin C$ respectively, and the magnitude is given by the number of boundary links in E_B which contain m in its node pair. Hence, it is clear that $c^1_m = \tilde{c}^1_m, c^2_m = \tilde{c}^2_m \forall m$ and $(S^i(C_{in}) - S^i(C_{out}))$ represents $\sum_{j \in C} B_1(j,i) + \sum_{j \in C} B_2(i,j)$. □

If we were to put all nodes into the same community, verify that all coefficients c^1_m (given by the column sums of the $|V| \times |V|$ matrix $(D - A)$ and c^2_m (given by the row sums of the $|V| \times |V|$ matrix $(D - A)$) are trivially 0. As a corollary, it immediately follows from that a $\sum_j B_1(j,i) = 0, \forall i$, i.e., all column sums of the matrix B_1 are equal to zero. Hence, the sum of all elements in B_1 equals zero. Similarly, $\sum_j B_2(i,j) = 0, \forall i$, i.e., all row sums of the matrix B_2 are equal to zero. Hence, the sum of all elements in B_1 equals zero.

$$\begin{aligned} \sum_{i,j} B_1(i,j) &= \sum_{i,j} [(D-A)S](i,j) = 0 \\ \sum_{i,j} B_2(i,j) &= \sum_{i,j} [S(D-A)](i,j) = 0. \end{aligned} \tag{14}$$

Also, it is easy to verify that equation 12 is independent of the subgraph under consideration and is generally true for any subgraph C^* . Therefore,

$$(S^i(C^*_{in}) - S^i(C^*_{out})) = \sum_{j \in C^*} (B_1(j,i) + B_2(i,j)). \tag{15}$$

Here j belongs to C^*, i.e., j belongs to a subgraph in the set of all subgraphs but C.

Our main theorem in the manuscript is reformulation of aggregate flux flowing through community boundaries into the Laplacian modularity quality function, which we now prove.

Theorem 1. *The net flux through the community boundaries can be evaluated as:*

$$\sum_{C \in \mathcal{P}} \sum_{i \in C} (S^i(C_{in}) - S^i(C_{out})) = \sum_{i,j} [(D-A)S + S(D-A)]\delta(i,j). \tag{16}$$

Proof. Using Property 1 and summing over all nodes $i \in C$, we get:

$$\sum_{i \in C} (S^i(C_{in}) - S^i(C_{out})) = \sum_{i \in C, j \in C} (B_1(j,i) + B_2(i,j)). \tag{17}$$

As $\delta(i,j)$ is 1 only if i and j belong to the same subgraph and 0 otherwise, the above equation can be rewritten as:

$$\sum_{i \in C}(S^i(C_{in}) - S^i(C_{out})) = \sum_{i \in C, j}[(D-A)S + S(D-A)]\delta(i,j). \tag{18}$$

Note, this holds for any subgraph $C \in \mathcal{P}$. As the quality function is additive over all (hard-partitioned) subgraphs, we get:

$$\sum_{C \in \mathcal{P}}\sum_{i \in C}(S^i(C_{in}) - S^i(C_{out})) = \sum_{i,j}[(D-A)S + S(D-A)]\delta(i,j). \tag{19}$$

This proves the theorem. □

Next we prove that the Laplacian modularity quality function is an unbiased measure. Consequently, the Laplacian modularity values the coarse one-way partition as zero for any S.

Theorem 2. *The relationship between the associative and dissociative quality function is:*

$$Q^{ass} + Q^{diss} = 0. \tag{20}$$

Proof. Recall the formulation of Q^{ass}, and Q^{diss}:

$$Q^{ass} = \sum_{C \in \mathcal{P}}\sum_{i \in C}(S^i(C_{in}) - S^i(C_{out})). \tag{21}$$

$$Q^{diss} = \sum_{C \in \mathcal{P}}\sum_{C^*}\sum_{i \in C}(S^i(C^*_{in}) - S^i(C^*_{out})). \tag{22}$$

Using equation 15 and summing over all nodes $i \in C$, we get:

$$\sum_{i \in C}(S^i(C^*_{in}) - S^i(C^*_{out})) = \sum_{i \in C, j \in C^*}(B_1(j,i) + B_2(i,j)). \tag{23}$$

The value $\sum_{i \in C, j \in C^*}(B_1(j,i) + B_2(i,j))$ represents the disassociation between subgraph C and one of the remaining subgraphs C^*. Adding over all subgraphs C^*, we get:

$$\sum_{C^*}\sum_{i \in C}(S^i(C^*_{in}) - S^i(V^{k*}_{out})) = \sum_{i \in C, j \notin C}(B_1(j,i) + B_2(i,j)). \tag{24}$$

Here the RHS follows from $j \in C^* \forall C^* \in \mathcal{P} \equiv j \notin C$, i.e., a node $j \in C^*$ over the set of all C^* implies node $j \notin C$. Let us define an operator $\varrho(i,j)$ which is 0 if node i and j belong to the same subgraph and 1 otherwise. Then the above equation can be rewritten as:

$$\sum_{C^*}\sum_{i \in C}(S^i(C^*_{in}) - S^i(C^*_{out})) = \sum_{i \in C, j}[(D-A)S + S(D-A)]\varrho(i,j). \tag{25}$$

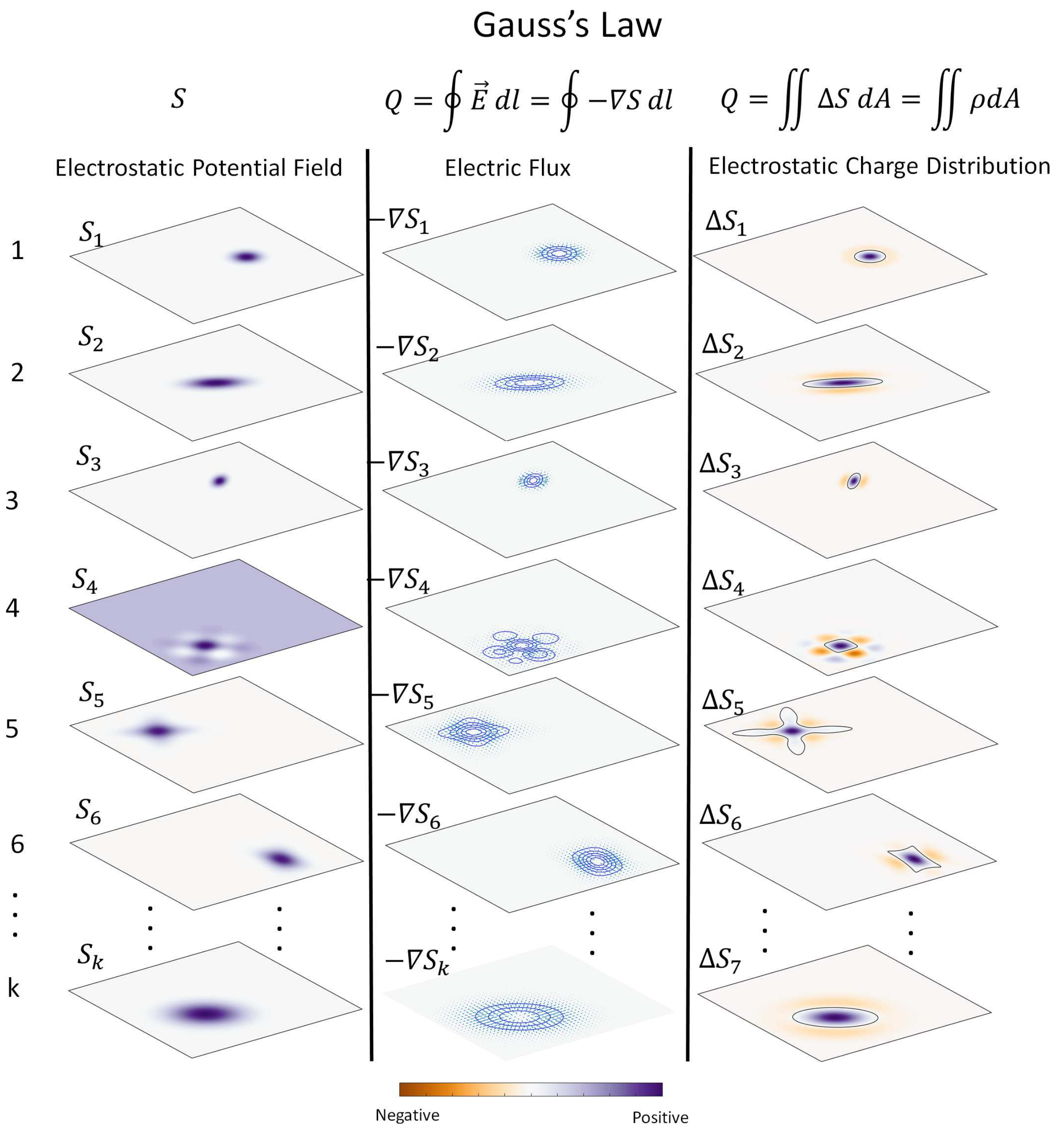

Figure 2: Gauss's law on a 2D domain *Left: Different electrostatic potential fields,* S_k*, at various points on a 2D domain. Higher potentials appear as darker shades of purple. Center: The electric flux calculated as the gradient of the potential field,* $-\nabla S_k$*. The isocontours for the electrostatic charge distribution, given as* ΔS_k *due to Gauss's law. Higher potentials appear as darker shades of purple, and the charge distribution varies from negative to positive as the color changes from orange to purple. The continuous 2D boundary maximizing the enclosed charge distribution is shown as a blue curve.*

Further, adding over all subgraphs $C \in \mathcal{P}$ we get:

$$\sum_{C\in\mathcal{P}}\sum_{C^*}\sum_{i\in C}(S^i(C^*_{in}) - S^i(C^*_{out})) = \sum_{i,j}[(D-A)S + S(D-A)]\varrho(i,j). \quad (26)$$

The term on LHS is precisely Q^{diss}. Using theorem 1 and definition of Q^{ass} we have:

$$Q^{ass} = \sum_{i,j}[(D-A)S + S(D-A)]\delta(i,j). \quad (27)$$

Adding equations 26 and 27 we get:

$$Q^{ass}+Q^{diss} = \sum_{i,j}[(D-A)S+S(D-A)](\delta(i,j)+\varrho(i,j)) = \sum_{i,j}[(D-A)S+S(D-A)]. \quad (28)$$

$\delta(i,j) + \varrho(i,j) = 1$ because nodes i and j can either belong to the same community or different communities and the sets are mutually exclusive. Using equation 14 we have:

$$Q^{ass} + Q^{diss} = 0. \quad (29)$$

This proves the theorem. Note the result holds independent of metric of similarity S, or in other words Q is an unbiased quality measure. Also, if A and S are both symmetrical, using elementary matrix algebra we get that $(D-A)S + S(D-A)$ is also symmetrical. As a result, it suffices to calculate $B = (D-A)S$ and obtain $(D-A)S + S(D-A)$ as $B + B^T$. □

We have now proved that the sum of all elements of the Laplacian modularity matrix, $(D-A)S + S(D-A)$ is zero independent of S. Hence, the Laplacian modularity matrix shares the key feature of unbiasedness with the modularity matrix, and values the coarse one-way partition to be zero. Also, it is possible to use existing modularity optimization techniques for Laplacian modularity optimization which is the topic of Section 2.5.

2.3 Connecting Gauss's law and our Gauss's law for networks

We tie our Gauss's law for networks to original Gauss's law. The original Gauss's law can be written in the following equivalent forms:

$$\mathcal{Q} = \oiint_S \vec{E}.ds = \iiint_V \Delta S.dv = \iiint_V \rho dv \quad (30)$$

Here $\mathcal{Q}$ is the electric charge, $\vec{E}$ is the electric field, Δ is the Laplace operator, S is the electric potential field, and ρ is the charge density. Our Gauss's law for networks is written in the following equivalent forms:

$$Q = \sum_{C\in\mathcal{P}}(S_{in}(C) - S_{out}(C)) = \sum_{i,j}[LS + SL]\delta(i,j) \quad (31)$$

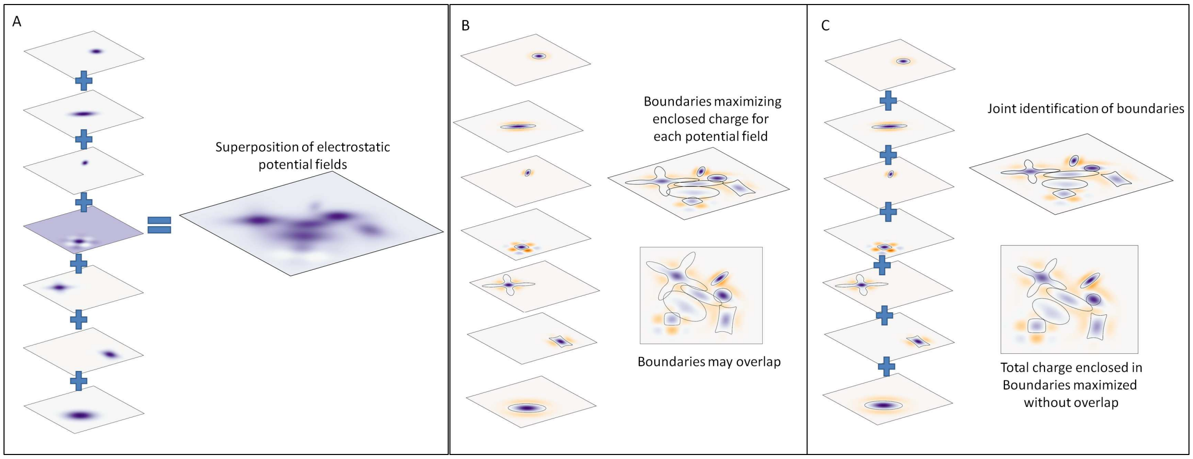

Figure 3: Partitioning a 2D domain using Gauss's law. *A: Superposition of the k potential fields on the 2D domain. B: Independently maximizing the enclosed positive charges gives rise to a soft partitioning of the 2D domain. C: Collectively maximizing the charge distributions outputs a consistent partition without overlap.*

Comparing the above formulas we see that the Laplacian modularity quality Q to be maximized corresponds to the net charge $\mathcal{Q}$ within an enclosed surface, the net connectivity flux flowing through community boundaries $\sum_{C\in\mathcal{P}}(S_{in}(C) - S_{out}(C))$ corresponds to the electric flux through a surface $\oiint_S E.ds$, and the sum over node pairs in the same community $\sum_{i,j}[LS + SL]\delta(i,j)$ corresponds to the volume integral $\iiint_V \Delta S$. Hence, the elements of the Laplacian modularity matrix, $LS + SL$, which we term connectivity density correspond to the local charge density ρ according to Gauss's law. An optimal partition according to Laplacian modularity is one that maximises the total sum of connectivity densities within modules, and can be interpreted as the net charge within modules due to Gauss's law.

We clarify how our method relates to Gauss's law by showing in Figures 2 and 3 an extended illustration of the principles highlighted in Figure 3, main manuscript. We now illustrate that the principles determining a partition of a region using Gauss's law can be extended to determine a partition of a network using Gauss's law for networks. Consider the synthetic network we used in Figure 4 in the main manuscript. For the purpose of illustration and analogy with Figures 2 and 3 only, we embed the our network of 16 nodes on a 4×4 grid. Then we consider a node's potential field to be a column of the connectivity potential matrix, S, shown in Figure 4 [1].

Each connectivity potential field for a node gives rise to connectivity flux, and using Gauss's law, a charge distribution on the nodes on the network (Figure 5). The sum of charge distributions over all nodes is zero, i.e., the column sums of the matrix LS are all zero. Observe that the boundaries shown using dotted blue lines maximizing the enclosed positive charges are not always consistent (For e.g., nodes 1 and 2). Hence, to find a consistent partition of the network

[1] In the main manuscript, we consider the connectivity potentials of a node i to be the ith column and row of S.

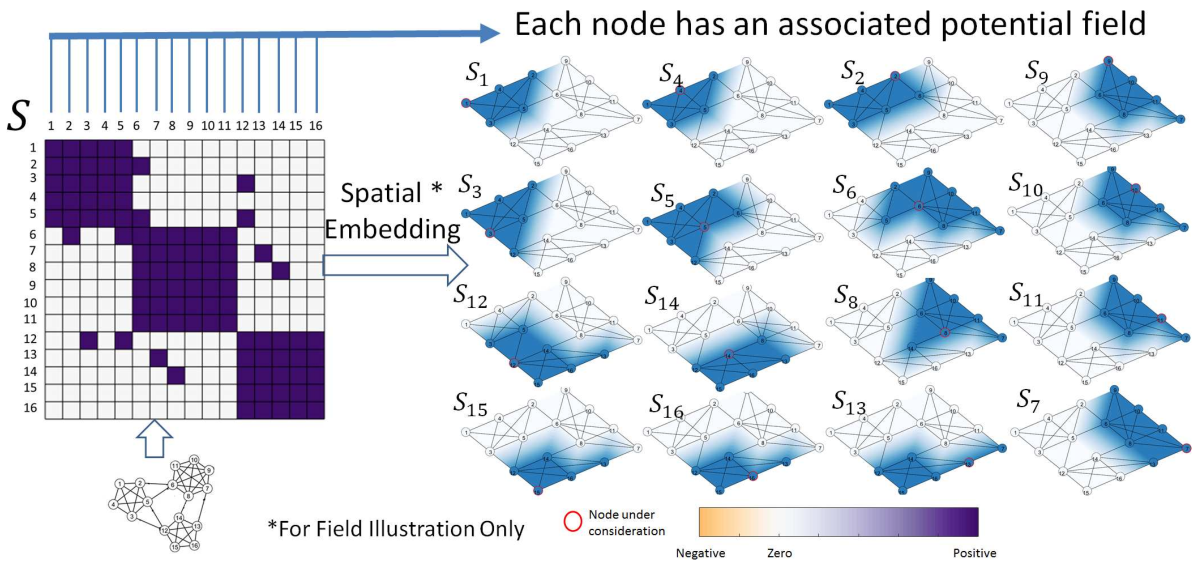

Figure 4: Connectivity potentials for Gauss's law for networks. *Left: The 0-1 connectivity potential matrix for 16 nodes in the network. Right: The connectivity potentials for each node viewed as potential field on a 4×4 grid, wherein each point in the grid corresponds to a node in the original network. Potential changes from 1 to 0 as color varies from purple to white.*

becomes a joint optimization over all independent charge distributions corresponding to the potential field for each node in a network. This is achieved by our Laplacian modularity quality function.

In summary, we run Gauss's law for each node in the network and the charges reside on the nodes. The electrostatic charge of a node in a community is the sum of charges on the node due to the potential fields of nodes in the community, i.e., charge of node i in community C is $\sum_{j \in C} LS(i,j)$.

We now explain our curvature metric. Our expression for curvature of a link is:

$$B(i,j) = d^i \left(S(i,j) - \frac{1}{d^i} \sum_{k \in N(i)} S(k,j) \right) + d^j \left(S(i,j) - \frac{1}{d^j} \sum_{k \in N(j)} S(i,k) \right). \tag{32}$$

In the term $\frac{1}{d^i} \sum_{k \in N(i)} S(k,j)$, the sum is over the similarity between neighbors of the node i and node j. This term does not equate to 1 unless all neighbors of node i are also connected to j. This can be intuitively interpreted as a curvature, because if two nodes have exactly the same set of neighbors, their curvature is zero. We also explained the variation of curvature as function of local connectivity in Figure 3 in the main manuscript. Note that the term in the modularity matrix for all four cases where a link is present between nodes i and j, each with degree 6, is $(1 - \frac{36}{2|E|})$, as modularity only considers the degree of a node and is oblivious to the topology of the graph. Similarly, the value in the modularity matrix for all four cases between two nodes

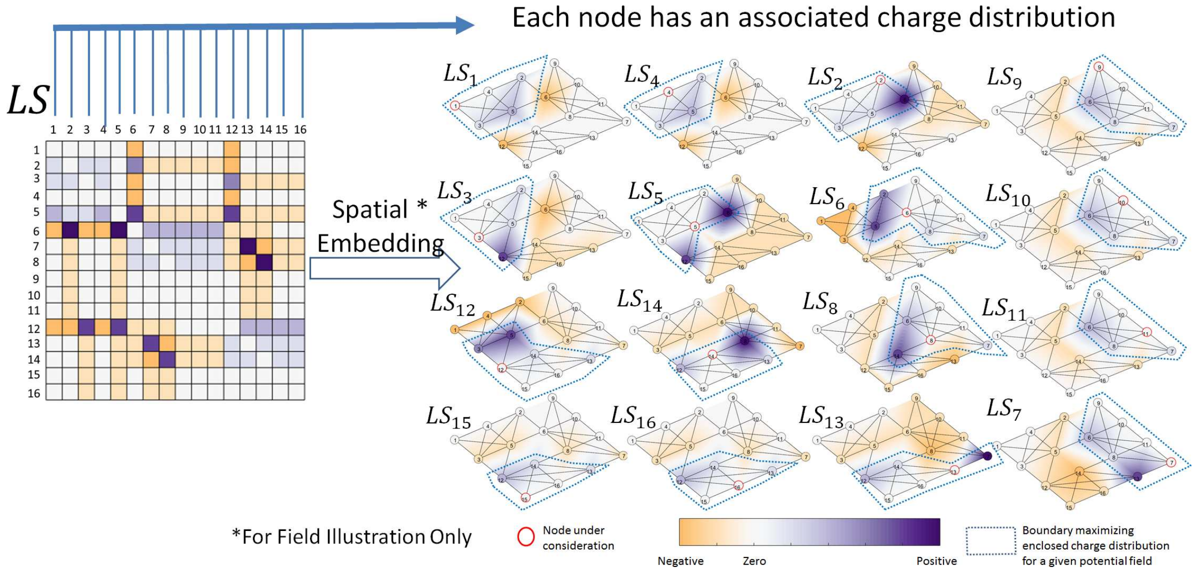

Figure 5: Connectivity distribution for Gauss's law for networks. *Left: The connectivity distribution matrix for 16 nodes in the network, wherein each column of LS corresponds to a connectivity distribution for the connectivity potentials defined by the columns of S. Right: The connectivity distribution for each node viewed as charge field on a 4×4 grid, wherein each point in the grid corresponds to a node in the original network. Charge changes from positive to negative as color varies from purple to red.*

i and j for the non-edges is always $-\frac{36}{2|E|}$. We also list all terms of the Laplacian modularity matrix for the synthetic network used in the main manuscript in Figure 6 for the connectivity potential matrix $S = I + A$, i.e., the adjacency matrix A plus self-loops. The network partition that maximizes the Laplacian modularity is shown as matrix blocks of color green, orange and blue.

As a final note we show that the connectivity field for any node i in the network is conservative, i.e., connectivity flux for node i along any closed path is zero. Recall from the main manuscript that the connectivity flux for a node i through a link E_b constitutive of nodes m and n is:

$$S^i(E_b) = \big(S(i,m) + S(m,i)\big) - \big(S(i,n) + S(n,i)\big). \tag{33}$$

Let a closed path consist of a set of n edges $E_{path} = \{E_1, E_2, \cdots E_n\}$. We need to prove that:

$$\sum_{E_k \in E_{path}} S^i(E_k) = \sum_{(m,n)\in E_k} \big(S(i,m) + S(m,i)\big) - \big(S(i,n) + S(n,i)\big) = 0. \tag{34}$$

Any two adjacent edges in the path, E_{k-1}, E_k possess exactly one common node, say node p. The signs of connectivity potential values, $S(i,p), S(p,i)$, corresponding to nodes i and p

in equation 33 will be positive for link E_{k-1} and negative for link E_k. This is true for every node p along a closed path including the start node (which is also the end node). Consequently, all the connectivity potential values cancel out and we are left with 0. For example, if a path consists of three nodes m, n, p constituting three links E_1, E_2, E_3 such that $(m, n) \in E_1, (n, p) \in E_2, (p, m) \in E_k$, we have:

$$\begin{aligned} S^i(E_1) + S^i(E_2) + S^i(E_3) =& \big((S(i,m) + S(m,i)) - (S(i,n) + S(n,i))\big) + \\ & \big((S(i,n) + S(n,i)) - (S(i,p) + S(p,i))\big) + \\ & \big((S(i,p) + S(p,i)) - (S(i,m) + S(m,i))\big) \\ =& \, 0 \end{aligned} \tag{35}$$

Thus, the connectivity field is conservative or its curl is zero.

2.4 Identifying overlapping nodes

Optimizing Q outputs a non-overlapping partition which are communities in the weak sense by construction. In the main manuscript we state that the positive terms in matrix $X^T L(S + S^T)$ resolve overlap, where X is a 0-1 indicator matrix for the (hard) partition of dimensions $|V| \times K$. $|V|$ is the number of nodes, K is the number of communities, and each row of X is all zeros except for a 1 indicating the node's membership to a community. Next, we formally define overlapping nodes using strong and weak definitions of a community.

Overlapping nodes: If a node associates with weak communities other than the one it is assigned to in the optimal partition, i.e., a node assigned to C satisfies the strong criterion (equation 1) for some other subgraphs in the set $C^*, C^* = \mathcal{P} \backslash C$, then the node is classified as an overlapping node with membership to these communities.

Figure 1 D,E,F displays the difference of internal and external boundary cohesion for the three communities, C^1, C^2, C^3 identified by optimizing the Laplacian modularity. Nodes 6 and 12 are correctly assigned to their respective cliques in the optimal partition, however, have multiple connections to the subgraph C^3, and emerge as overlapping nodes with positive affinity (shaded blue) in the vector $S(C^3_{in}) - S(C^3_{out})$ (Figure 1F).

We prove that $K \times \mid V \mid$ matrix $X^T B_1 + (B_2 X)^T$ can efficiently identify strong communities by checking for positive terms where X is a $[0-1]$ indicator matrix. $X^T B_1 + (B_2 X)^T$ is equivalent to $X^T(B_1 + B_2^T)$. Given a partition of nodes into *weak* communities, strong or weak membership, and hence, overlap of node i is determined by checking if the node satisfies the criterion of a *strong* community for a subgraph other than the one it is assigned in the optimal partition, i.e., we check:

$$S^i(C^*_{in}) > S^i(C^*_{out}), \forall C^*. \tag{36}$$

Equivalently, $(S^i(C^*_{in}) - S^i(C^*_{out})) > 0, \forall C^*$. Using equation 15, this translates to checking if:

$$\sum_{j \in C^*} (B_1(j,i) + B_2(i,j)) > 0, \forall C^*. \tag{37}$$

$B = LS + SL$

	1	2	3	4	5	6	7	8	9	10	11	12	13	14	15	16
1	0	1	1	0	2	-4	0	0	0	0	0	-4	0	0	0	0
2	1	0	2	1	1	8	-2	-2	-2	-2	-2	-4	0	0	0	0
3	1	2	0	1	1	-4	0	0	0	0	0	7	-2	-2	-2	-2
4	0	1	1	0	2	-4	0	0	0	0	0	-4	0	0	0	0
5	2	1	1	2	0	9	-2	-2	-2	-2	-2	8	-2	-2	-2	-2
6	-4	8	-4	-4	9	0	3	3	2	2	2	-2	-2	-2	0	0
7	0	-2	0	0	-2	3	0	2	1	1	1	-2	9	-4	-2	-2
8	0	-2	0	0	-2	3	2	0	1	1	1	-2	-4	9	-2	-2
9	0	-2	0	0	-2	2	1	1	0	0	0	0	-2	-2	0	0
10	0	-2	0	0	-2	2	1	1	0	0	0	0	-2	-2	0	0
11	0	-2	0	0	-2	2	1	1	0	0	0	0	-2	-2	0	0
12	-4	-4	7	-4	8	-2	-2	-2	0	0	0	0	3	3	2	2
13	0	0	-2	0	-2	-2	9	-4	-2	-2	-2	3	0	2	1	1
14	0	0	-2	0	-2	-2	-4	9	-2	-2	-2	3	2	0	1	1
15	0	0	-2	0	-2	0	-2	-2	0	0	0	2	1	1	0	0
16	0	0	-2	0	-2	0	-2	-2	0	0	0	2	1	1	0	0

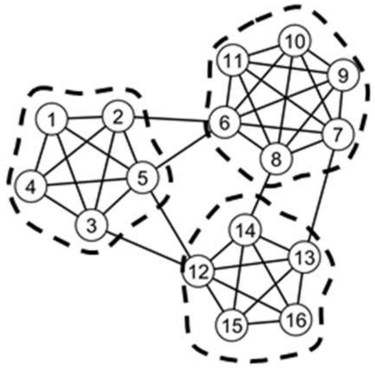

Network

Laplacian modularity matrix

Figure 6: Laplacian modularity matrix. *The Laplacian modularity matrix* $LS + SL$ *for the network shown in left and using* $S = A + I$.

Now consider the $(k, i)^{th}$ term of the matrix $X^T(B_1 + B_2^T)$. Using elementary matrix multiplication properties, it can be verified that:

$$(X^T(B_1 + B_2^T))(k, i) = \sum_{j \in C}(B_1(j, i) + B_2(i, j)). \tag{38}$$

If node i belongs to community C in the strong sense if $(X^T(B_1 + B_2^T))(u, i) > 0, \forall u \in k*$ where $k*$ are the indices of subgraphs $C^* \in \mathcal{P}$. It follows that presence of o positive terms in the ith column implies that node i is classified as an overlapping node with membership to o subgraphs C^u corresponding to the positive indices u, s.t., $(X^T(B_1 + B_2^T))(u, i) > 0$. Thus, $X^T(B_1 + B_2^T)$ encodes all information about overlap between different subgraphs and overlapping nodes can be efficiently identified by identifying positive entries in the matrix. $X^T(B_1 + B_2^T)$ can be equivalently written as $X^T(LS + (SL)^T)$ or $X^T(LS + LS^T)$ as described in the main manuscript. Using equation 12, it follows that each element in $(X^T(B_1 + B_2^T))$ encodes the net connectivity flux for all nodes through each subgraph boundary, and hence, overlapping nodes are simply nodes with net positive connectivity flux to multiple communities as described in the main manuscript.

2.5 Optimization of Laplacian modularity

Modularity optimization is provably NP-hard and the structural similarity of the Laplacian modularity and modularity matrix makes it unlikely that an efficient algorithm exists for Laplacian modularity optimization. Several heuristics and approximate algorithms exist for modularity optimization such as spectral optimization (*New06a*), simulated annealing (*LL10*) and greedy agglomerative techniques (*New04b*). These methods can be adapted for optimizing the Laplacian modularity quality function. We use the Louvain method (*BGLL08*) to optimize Laplacian modularity, Q, in all our experiments due to its ease, speed and accuracy.

Algorithm 1 Louvain Algorithm

procedure LOUVAIN(B)
    $W \leftarrow B$
    $\mathcal{M} \leftarrow 1 : |V|$ ▷ $|V|$ is size of W
    while 1 **do**
        $Q \leftarrow W$
        $flag \leftarrow true$
        while $flag = true$ **do**
            $flag \leftarrow false$
            for $i := 1\mathbf{to}|V|$ **do**
                $\Delta \leftarrow Q(i,:) - Q(i,\mathcal{M}(i)) + W(i,i)$
                if $max(\Delta) > 0$ **then**
                    $j \leftarrow index(max(\Delta))$
                    $Q(:,j) \leftarrow Q(:,j) + W(:,j)$
                    $Q(:,\mathcal{M}(i)) \leftarrow Q(:,\mathcal{M}(i)) - W(:,i)$
                    $\mathcal{M}(i) \leftarrow j$
                    $flag \leftarrow true$
                end if
            end for
        end while
        $h \leftarrow h + 1$
        $\mathcal{P}(h) \leftarrow \mathcal{M}$ ▷ Assign labels
        $W \leftarrow X^T W H$ ▷ $H \leftarrow indicator(C(h))$
        $\mathcal{D}_h^n \leftarrow trace(W)$
        if $\mathcal{D}_h^n - \mathcal{D}_{h-1}^n = 0$ **then**
            break
        end if
    end while
    return $\mathcal{P}$ ▷ The hierarchical labels are stored in $\mathcal{P}$
end procedure

Louvain: The Louvain method is the current state-of-the-art method for optimizing modularity (*BGLL08*). The generalized version of the method adapted for optimizing our quality function Q is a greedy optimization method that attempts to optimize Q in two steps. First, the method looks for small communities by optimizing Q locally. Second, it aggregates nodes belonging to the same community and builds a new network whose nodes are the commu-

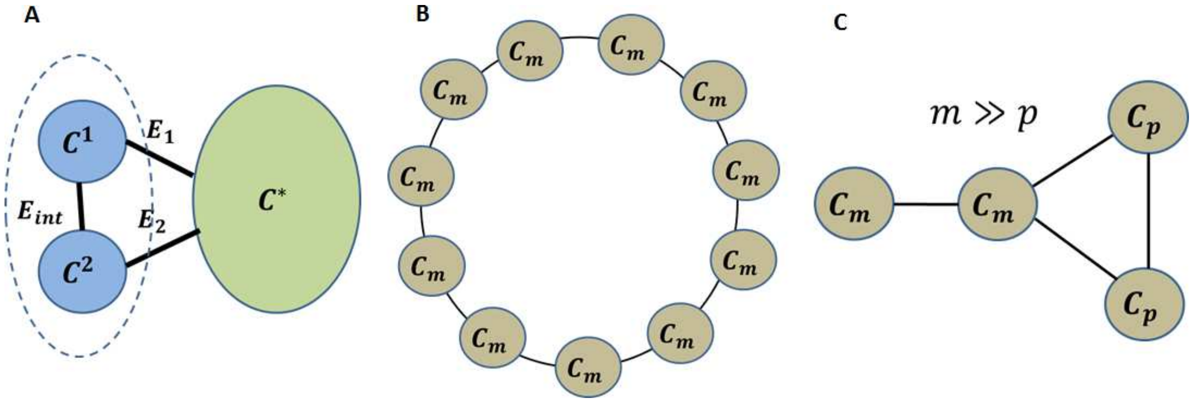

Figure 7: Resolution limit. *A: The Laplacian modularity framework does not suffer from the traditional resolution limit, i.e., C^1 and C^2 are not merged by the Laplacian modularity quality function if C^1 and C^2 are strong communities. B: Ring of cliques, C_m C: Pairwise identical cliques, C_m and C_p*

nities. These steps are repeated iteratively until a maximum of Q is attained. The pseudo code of Louvain method adapted to optimize the Laplacian modularity matrix is in Algorithm 1. The input B is the matrix $[(D-A)S + S(D-A)]$. In Algorithm 1, in the statement $\Delta \leftarrow Q(i,:) - Q(i, M(i)) + W(i,i)$, the terms $Q(i, M(i)), W(i,i)$ are to be interpreted as $Q(i, M(i))\mathbb{1}, W(i,i)\mathbb{1}$ respectively, where $\mathbb{1}$ is a $|V|$ length row vector of ones.

2.6 Analysing the resolution limit of Laplacian modularity

Here we examine the resolution limit of our approach. We analyze the resolution limit of Laplacian modularity for general similarity matrices, S and discuss the resolution limit in reference to the heat kernel in the next section.

Consider two partitions of the network, Y and Z. These two partitions are identical in all subgraphs represented as C^*, except in Z two subgraphs C^1 and C^2 are merged, whereas in Y they are considered as separate subgraphs (see Figure 7 A). Because, Q is expressed as a sum over subgraphs, the corresponding value of Q for these two partitions are then given as:

$$Q^Y = \sum_{C^*} (S_{in}(C^*) - S_{out}(C^*)) + (S_{in}(C^1) - S_{out}(C^1)) + (S_{in}(C^2) - S_{out}(C^2)). \quad (39)$$

$$Q^Z = \sum_{C^*} (S_{in}(C^*) - S_{out}(C^*)) + (S_{in}(C^1, 2) - S_{out}(C^1, 2)). \quad (40)$$

The contribution to Q^Y and Q^Z over subgraphs C^* is the same. Then, the difference in quality of these two partitions is $\Delta Q = Q^Y - Q^Z$.

$$\Delta Q = ((S_{in}(C^1) - S_{out}(C^1)) + (S_{in}(C^2) - S_{out}(C^2)) - (S_{in}(C^1, 2) - S_{out}(C^1, 2)). \quad (41)$$

Expanding as a sum over nodes we get:

$$\begin{aligned}\Delta Q = &\sum_{i\in C^1}(S^i(C^1_{in}) - S^i(C^1_{out})) + \sum_{i\in C^2}(S^i(C^2_{in}) - S^i(C^2_{out})) \\ &- \sum_{i\in C^{1,2}}(S^i(C^{1,2}_{in}) - S^i(C^{1,2}_{out})).\end{aligned} \tag{42}$$

To simplify the calculations, we only consider the contributions from $(D-A)S$ in the Laplacian modularity matrix and the counterpart $S(D-A)$ can be analyzed similarly. We distinguish three types of links: between C^1 and C^2 (E_{int}), between C^1 and the rest of the network C^* (E_1) and between C^2 and the rest of the network C^* (E_2). Using the property that S^i is a sum over links, we separate terms in ΔQ as follows:

$$\sum_{i\in C^1}(S^i(C^1_{in}) - S^i(C^1_{out})) = \sum_{i\in C^1}\left(\sum_{E_1}(S(m,i) - S(n,i)) + \sum_{E_{int}}(S(m,i) - S(n,i))\right) \tag{43a}$$

$$\sum_{i\in C^2}(S^i(C^2_{in}) - S^i(C^2_{out})) = \sum_{i\in C^2}\left(\sum_{E_2}(S(m,i) - S(n,i)) + \sum_{E_{int}}(S(m,i) - S(n,i))\right) \tag{43b}$$

$$\sum_{i\in C^{1,2}}(S^i(C^{1,2}_{in}) - S^i(C^1_{out})) = \sum_{i\in C^{1,2}}\left(\sum_{E_1}(S(m,i) - S(n,i)) + \sum_{E_2}(S(m,i) - S(n,i))\right). \tag{43c}$$

We now analyze ΔQ over two cases. Let $\hat{E}$ be the set of nodes induced by links E. In the first case there are no common nodes induced by links $[E_1, E_2]$, $[E_1, E_{int}]$ and $[E_2, E_{int}]$, i.e., $\hat{E}_1 \cap \hat{E}_2 = \phi$, $\hat{E}_1 \cap \hat{E}_{int} = \phi$ and $\hat{E}_2 \cap \hat{E}_{int} = \phi$. Second we analyze for the case where nodes may be common in the sets. We consider S of the form $S(i,j) > 0$ only if there is a direct connection between i and j.

Case 1: If there are no common induced nodes, then it follows that for $\sum_{E_1}$, $S(m,i) > 0, S(n,i) > 0$ only if $i \in C^1$. Similarly, for $\sum_{E_2}$, $S(m,i) > 0, S(n,i) > 0$ only if $i \in C^2$. Hence, we have:

$$\sum_{i\in C^{1,2}}\left(\sum_{E_1}(S(m,i) - S(n,i))\right) = \sum_{i\in C^1}\left(\sum_{E_1}(S(m,i) - S(n,i))\right) \tag{44a}$$

$$\sum_{i\in C^{1,2}}\left(\sum_{E_2}(S(m,i) - S(n,i))\right) = \sum_{i\in C^2}\left(\sum_{E_2}(S(m,i) - S(n,i))\right). \tag{44b}$$

Therefore these contributions cancel out in ΔQ and we are left with:

$$\Delta Q = \sum_{i\in C^1}\sum_{E_{int}}(S(m,i) - S(n,i)) + \sum_{i\in C^2}\sum_{E_{int}}(S(m,i) - S(n,i)). \tag{45}$$

For E_{int}, the nodes m and n are complementary for the subgraphs C^1 and C^2, i.e., an internal node to C^1 is an external node to C^2 and vice versa. Without loss of generality, let us assume

m belongs to C_1 and n belongs to C_2. If we assume a constant value of $S(m,i) \forall i$, say $S(m)$, then the sum over C_1 is $d^m(C^1)S(m)$, where $d^m(C^1)$ is the number of connections of m to subgraph C^1. Similarly, sum over C_2 is $d^m(\bar{C}^1)S(m)$ where $d^m(\bar{C}^1)$ is the number of external connections of m to C^2 (in this case the external degree of node m). Hence, equation 45 reduces to:

$$\Delta Q = \sum_{E_{int}} \bigg((d^m(C^1) - d^m(\bar{C}^1))S(m) + (d^n(C^2) - d^n(\bar{C}^2))S(n) \bigg). \tag{46}$$

Now under the assumption that C^1 and C^2 are LS-sets or strong communities as per the definition of Radicchi (*RCC$^+$04*), $(d^m(C^1) - d^m(\bar{C}^1))$ and $(d^n(C^2) - d^n(\bar{C}^2))S(n)$ are always greater than 0, and hence $\Delta Q > 0$. In other words, the Laplacian modularity does not suffer from the traditional resolution limit of modularity (*FB07*). Note that equation 46 is derived by taking a difference of the Laplacian modularity quality function, which is turn is derived from our weak definition of communities. Hence, the Laplacian modularity quality function does not suffer from the traditional resolution limit of modularity as long as the nodes induced by boundary links are all disjoint and S is local.

Case 2: For the case when some induced nodes are common, the equation 44 does not hold, and hence, simplification doesn't occur. So we investigate the Laplacian modularity on a per node basis so as to analyze the resolution limit of identifiable modules by the Laplacian modularity without any assumption on the number of induced nodes which are common. Note that the Laplacian modularity quality function is derived from the weak definition of communities, whereas this analysis stems from the strong definition of communities. Let $S(i,C) = \sum_{j \in C} \big(S(i,j) + S(j,i) \big)$, i.e., $S(i,C)$ is the reciprocal sum of connectivity coefficients of a node i to nodes in a subgraph. Then we have:

$$\sum_{i \in C} (S^i(C_{in}) - S^i(C_{out})) = \sum_{i \in C} d^i(\bar{C})S(i,C) - \sum_{j \in C^*} d^j(C)S(j,C). \tag{47}$$

Here $d^i(\bar{C})$ indicates the external degree of node i, j does not belong to subgraph C, and $d^j(C)$ indicates the external degree contribution of node j to subgraph C. Equation 47 constitutes subgraph C's contribution to Q. Then node i's total contribution to Q, $\mathcal{S}^i(Q)$ is:

$$\mathcal{S}^i(Q) = d^i(\bar{C})S(i,C) - \sum_{C^p \in C^*} d^i(C^p)S(i,C^p). \tag{48}$$

This is because node i's positive contributions to Q occur as an internal boundary node of subgraph C and negative contributions occur as an external boundary node to some subgraphs in C^*. We have an additional constraint that $d^i(\bar{C}) = \sum_{C^p \in C^*} d^i(C^p)$, i.e., the total external connections of a node i is equal to the sum of (external) degree contributions to all other subgraphs. So for $\mathcal{S}^i(Q) \geq 0$, we get the relationship:

$$S(i,C) \geq \frac{\sum_{C^p \in C^*} d^i(C^p)S(i,C^p)}{\sum_{C^p \in C^*} d^i(C^p)}. \tag{49}$$

This equation intuitively means that the net internal connectivity of a node to a subgraph should be greater or equal to the weighted sum of net external connectivity to all other subgraphs. If the above equation holds for all nodes in a subgraph, then it is considered a strong module. If we consider S to be equal to the adjacency matrix A, we have $S(i, C) = 2d^i(C)$ and $S(i, C^p) = 2d^i(C^p)$. Equation 49 then reduces to:

$$d^i(C) \geq \frac{\sum_{C^p \in C^*} (d^i(C^p))^2}{\sum_{C^p \in C^*} d^i(C^p)}. \tag{50}$$

Using Cauchy-Schwarz inequality, $x_1^2 + x_2^2 \dots x_n^2 \geq (x_1 + x_2 \dots x_n)^2/n$, we get:

$$d^i(C) \geq \frac{\sum_{C^p \in C^*} (d^i(C^p))^2}{\sum_{C^p \in C^*} d^i(C^p)} \geq \frac{\left(\sum_{C^p \in C^*} (d^i(V^p))\right)^2}{|k| \sum_{C^p \in C^*} d^i(V^p)}. \tag{51}$$

Here k is the number of subgraphs in C^* node i has connections to. Using the relation $d^i(\bar{C}) = \sum_{C^p \in C^*} d^i(C^p)$ we get:

$$d^i(C) \geq \frac{d^i(\bar{C})}{|k|}. \tag{52}$$

Instead of requiring that the internal degree exceeds the external degree as in a strong community of (*RCC$^+$04*), the Laplacian modularity quality function for $S = A$ requires that the internal degree of a node exceeds the average external degree over subgraphs the node is connected to. Furthermore, if all nodes in a subgraph have external connections to a maximum of one other subgraph then $k = 1$ and we recover the strong definition of a community (*RCC$^+$04*). Some recently proposed methods such as modularity density (*CNS13*), excess modularity density (*CSB18*) and surprise maximization (*AM13*) also mitigate the resolution limit. However, unlike these quality metrics, Laplacian modularity quality function can be directly optimized using modularity based methods. Overall, the resolution limit of Laplacian modularity is lower than the traditional resolution limit of modularity, which we validate on networks presented in (*FB07*).

Figure 7 demonstrates the efficacy of our construct against the traditional resolution limit (*FB07*) for $S = A$. On a ring of cliques C_m of size m, our approach always identifies the correct partition with highest Q_{LS} irrespective of size of m for $m > 2$. In contrast, modularity always merges cliques and we observed that Infomap merged cliques for small m. On pairwise identical cliques C_m, C_p of size m and p respectively connected as shown in Figure 7, Q_{LS} never merged the smaller cliques C_p irrespective of the difference in sizes, unlike modularity which merged the smaller cliques C_p into one subgraph. We can conclude from the above analysis that when S is local, the resolution limit of Laplacian modularity is lower than the traditional resolution limit of modularity both in a theoretical and practical setting, and we are guaranteed never to miss strong modules when all boundary links induce different nodes.

2.7 Multi-resolution extension of Laplacian modularity

Hierarchical organization of communities in most real-world networks suggests that there is no *best* partition as derived by quality functions as we mentioned in the main manuscript. Hence, a quality function should be amenable to the resolution of analysis to reveal hierarchy. We distinguish between multi-resolution analysis and identifying hierarchical organization in networks as follows: A multi-resolution method outputs partitions for any input resolution parameter, whereas hierarchical analysis corresponds to a subset of these partitions which are assessed to be of high quality, or correspond to topological scales of the network.

2.7.1 Multi-resolution Laplacian, L_ω

We provide a multi-resolution extension of the Laplacian modularity quality function akin to multi-resolution modularity (*RB06*). The multi-resolution extension of the Laplacian modularity is done using resolution parameter ω as follows:

$$Q = \sum_{i,j}[(D - \omega A)S + S(D - \omega A)]\delta(i,j) = \sum_{i,j}[L_\omega S + SL_\omega]\delta(i,j). \quad (53)$$

Where L_ω is the resolution dependent Laplacian. $\omega = 0$ outputs the trivial partition where all nodes are grouped into one community. Varying ω in the range $[0, 1]$ outputs multi-resolution partitions. In order to determine the relevant hierarchies among the set of partitions output by varying, ω, we construct a stability curve using the equation:

$$r(\omega) = max_X trace[X^T B_\omega X]. \quad (54)$$

where B_ω is the matrix given by $[L_\omega S + SL_\omega]$. Smaller values of ω favor coarser partitions. However, the ω lacks an intuitive interpretation, unlike time t, in the Laplacian modularity using the heat kernel. We shall demonstrate the stability curve for alternate definitions of connectivity potentials in section 8.

2.7.2 Connectivity potential matrix with self-similarity

The resolution of analysis can be amended by changing the self-similarity value $S(i,i)$ in the connectivity potential matrix. To see this, let the self-similarity of nodes be represented by the diagonal matrix S_s. Let the connectivity potential (similarity) matrix be $S = S_{\not s} + S_s$ where $S_{\not s}$ is the matrix of all pairwise similarities but self-similarities. Then the Laplacian modularity matrix can be decomposed as:

$$LS + SL = (LS_s + S_s L) + (LS_{\not s} + S_{\not s} L). \quad (55)$$

The first matrix in the parenthesis, $LS_s + S_s L$ has positive entries only along its diagonal, and hence, outputs the trivial partition where each node is assigned to an independent community.

Whereas the term $LS_{\not{s}} + S_{\not{s}}L$ identifies communities according to our Gauss's law for networks. Hence, the self-similarity of a node can be viewed as resolution parameter, with higher self-similarity values favouring communities of a smaller resolution. Note that the Laplacian of a matrix is unaffected by self-loops, as the diagonal entries in $L = D - A$ cancel each other out. The Laplacian modularity can be extended to a multi-resolution method by varying the similarity matrix S as $S = \omega S_s + S_{\not{s}}$. We can set S_s to be the identity matrix I, if unknown. However, a methodology to identify hierarchy among these partitions at multiple resolutions is not straightforward.

2.7.3 Longer path-length or higher order Laplacian

Another strategy to account for resolution is to consider averaging over longer connected neighborhoods by considering the Laplacian corresponding to powers of A, i.e., L corresponding to $A^2, A^3, \cdots$. For example, the quality function corresponding to A^2 is:

$$Q = \sum_{i,j} [(D_2 - A^2)S + S(D_2 - A^2)]\delta(i,j). \tag{56}$$

Here D_2 are the row or column sums of the symmetric matrix, A^2. Similarly, we can consider higher powers of the Laplacian with the same structural properties of L, i.e., the row and column sums of L^n are 0. The corresponding quality functions are of the form:

$$Q_n = \sum_{i,j} [L^n S + S L^n]\delta(i,j). \tag{57}$$

Where Q_n is the quality function corresponding to the nth power of the Laplacian. However, there is no way to determine relevant hierarchies from the partitions output by the Laplacian corresponding to different powers of A (equation 56), or by considering a higher order Laplacian, L^n of varying n (equation 57). As a result, we advocate the Laplacian modularity using the heat kernel as a principled way to determine relevant hierarchies using the stability curve, the topic of section 3.

2.8 Resistor network interpretation of Laplacian modularity

A large connectivity flux through a community boundary is encoded as high connectivity density among the internal nodes in the Laplacian modularity matrix due to our network analog of Gauss's law. This can be interpreted using resistor networks. The well-known relationship between current vector $Curr$, voltage vector Vol and the Laplacian L in resistor networks is (*Wu04*):

$$L * Vol = Curr. \tag{58}$$

Now if we were to sequentially apply voltages to each node, where the ith voltage vector corresponds to the connectivity potential values (the ith row and column of S), then the Laplacian

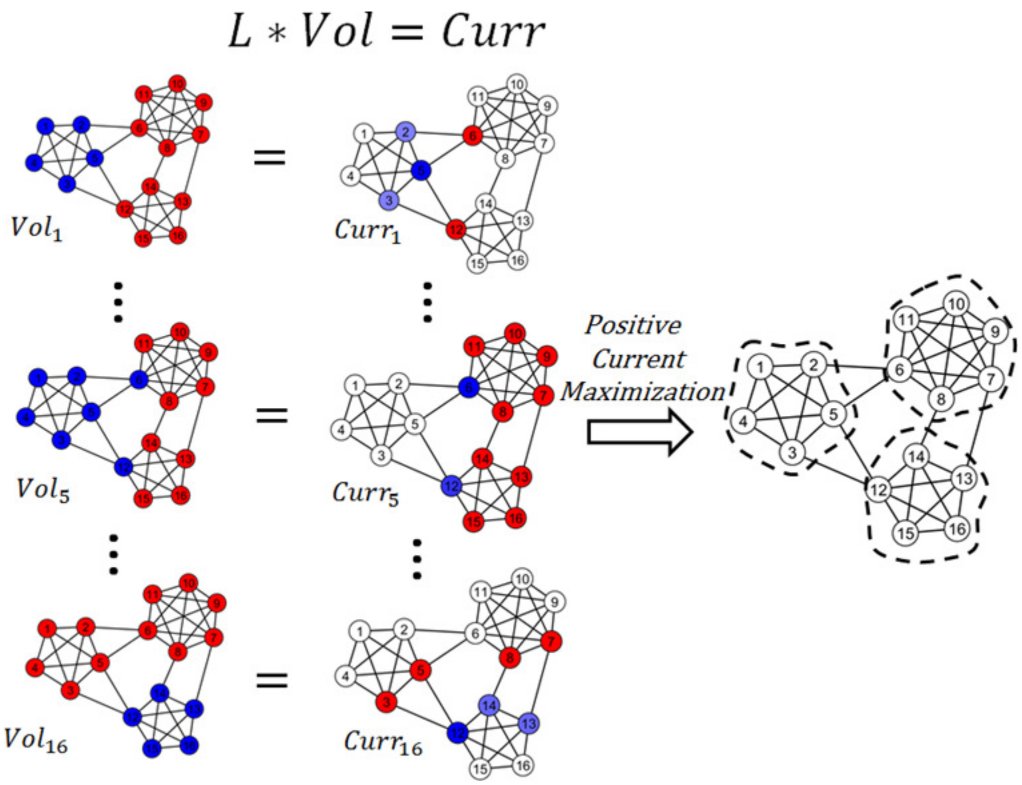

Figure 8: Resistor network interpretation of Laplacian modularity. *The plots to the left show the voltage applied to each node in the network. Blue indicates a unit positive potential applied to a node and its neighbors, whereas red indicates a grounded node. The plots in the middle show the net current flow through each node in the network as a result of the applied voltages on nodes in the network. The current flow changes from positive to negative as the node colors change from blue to red. The plot on the right indicates that we are in effect maximizing the net positive current in the network to identify modules in our Laplacian modularity framework.*

modularity matrix is equal to the net current flow matrix. Let B_1, B_2 be the matrices LS, SL respectively. Then the ith column of B_1 is the current induced by applying voltages equal to i^{th} column of S at the nodes in the network, and the ith column of B_2 is the current induced by applying voltages equal to i^{th} row of S at the nodes in the network. Note that the current is the net current through a node, implicitly compensated by an external source or sink of current connected to the node, so as to respect Kirchhoff's current law. Maximizing the Laplacian modularity matrix then effectively translates to finding subgraphs such that the positive current flow is maximized. The net current flow in the entire network is zero. This is graphically illustrated in Figure 8. The plots on the left show the applied voltage and the induced current at the different nodes due to equation 58 and its symmetric counterpart, $Vol^T * L = Curr^T$. All induced currents are succinctly represented by the Laplacian modularity matrix, and optimizing Q results in a partition wherein the positive currents are maximized (Plot to the right in Figure

8). This also connects to maximizing the charge distribution within modules as discussed in equation 31.

3 Laplacian modularity using heat kernel

In this section we discuss the Laplacian modularity quality function using the heat kernel as a measure of connectivity potential. We first introduce the heat kernel as a solution to the diffusion equation and establish connections to random walks. We mention properties of the Laplacian modularity using the heat kernel, discuss the identification of overlap and hierarchy, and propose a parameter-free algorithm for identifying the best partition of a network. We also discuss the resolution limit of our approach and compare it to the resolution limit of modularity.

3.1 Markov chains and the heat kernel

We describe the heat kernel and its relation to Markov chains. A Markov chain is a mathematical system that undergoes state transitions following the Markov property, i.e., future states depend only upon the present state and not on the past, or that the system is memoryless. Random walks can be viewed as special cases of finite time-reversible Markov chains (*Kei79*). A random walk starting from a node on a graph evolves with time, where time is either discrete (set of positive integers) or continuous (set of positive real numbers). Here, we briefly discuss the formalism of discrete and continuous time random walks, the inter-relationship between the two, and relevant spectral decompositions which are used in subsequent sections for proving some properties and for computational efficiency. For a general discussion on random walks, the reader is asked to refer the excellent survey by Lovász (*Lov96*).

First, we detail basics useful for analyzing discrete and continuous random walks. The probability transition matrix M for the discrete walk is given by $M = D^{-1}A$, indicating the probability of leaving a node is evenly split among all its adjacent neighbors. As M is asymmetric in general, it is useful to convert it into a symmetric normalized matrix N for spectral analysis by considering $N = D^{1/2}MD^{-1/2}$ [2]. The study of continuous time walks is simplified by introducing the random walk Laplacian $\mathbb{L}$ and its normalized counterpart $\mathcal{L}$. Mathematically, $\mathbb{L} = I - M$ and $\mathcal{L} = I - N$ where I is an identity matrix of size N. Let the spectral decomposition of the normalized Laplacian be given by $\mathcal{L} = \Phi\Lambda\Phi^T$ where Λ is a diagonal matrix with the ordered eigenvalues, i.e., $0 = \lambda_1 \leq \lambda_2 ... \leq \lambda_N \leq 2$, Φ is the matrix with corresponding eigenvectors as columns. Then the eigen decomposition of the normalized transition matrix is $\Phi\Lambda'\Phi^T$ with same set of eigenvectors and eigenvalues related as $\Lambda' = I - \Lambda$ (see (*Chu97*)).

[2] Note that our method is not a spectral method and our use of eigenvalues and eigenvectors describe how they can be used in standard ways to reduce the computation involved with the matrix exponential in the heat kernel, and prove some properties of the Laplacian modularity matrix using the heat kernel.

3.1.1 Discrete time random walk

Discrete random walks take place over discrete time steps starting from $t = 0$ over the positive integer domain. The rule of walk on the graph can be expressed as:

$$P_t = M^T P_{t-1}. \tag{59}$$

Hence iterating, we get:

$$P_t = (M^T)^t P_0. \tag{60}$$

Here P_t is the probability distribution (summing to 1) over the nodes of the graph starting from the initial distribution P_0 and depends on the transpose of the state transition matrix, M^T. The stationary distribution or the probability of being at a vertex after the walk has reached equilibrium (after long time or at $t = \infty$) is independent of the initial distribution. Formally $\pi = M^T\pi$ or $\pi = \mathbb{1}D/vol$ where $\mathbb{1}$ is a vector with all coordinates 1 and $vol = \sum_u d_u$ called the volume of the graph.

The distribution at intermediate times, i.e., M^t can be determined using the spectrum of the normalized Laplacian or can be calculated using elementary matrix multiplication. The spectral decomposition of the transition matrix is $M = D^{-1/2}\Phi\Lambda'\Phi^T D^{1/2}$ and $M^t = D^{-1/2}\Phi\Lambda'^t\Phi^T D^{1/2}$. Hence, we have:

$$p_t(i,j) = \sum_{k\geq 1} \lambda_k'^t \Phi_k(i)\Phi_k(j)\sqrt{\frac{d_j}{d_i}} = \pi_j + \sum_{k\geq 2} \lambda_k'^t \Phi_k(i)\Phi_k(j)\sqrt{\frac{d_j}{d_i}}. \tag{61}$$

Here $p_t(i,j)$ is the probability of starting at i and reaching j in t steps and is the $(i,j)^{th}$ entry of the matrix M^t and tends to π_j in the long time limit as $\mid \lambda_k' \mid\leq 1$. We have used the orthogonality of eigenvectors $\Phi_i^T\Phi_j = \delta(i,j)$). Note that P_t is a $|V|\times 1$ vector whereas p_t is a $|V|\times|V|$ matrix. The limitation of discrete time steps is overcome in the continuous time limit and is the topic of our next discussion.

3.1.2 Continuous time random walk

The difference between the discrete and continuous setting of the random walk is the waiting time between hops of a random walker, i.e., constant 1 for the discrete walk (as $t = 0, 1, 2\ldots$) and an exponential distribution for continuous time random walks (*KS60*). Recall, the exponential distribution characterizes the waiting time in a Poisson process and hence the number of jumps completed by a random walker at time t is a Poisson distribution (*SR14*). The rule of walk is expressed by the Kolmogorov differential equation:

$$\frac{dp_t}{dt} = -\mathbb{L}p_t. \tag{62}$$

We solve the Kolmogorov equation to determine the probability distribution over all pairs of state spaces at time t and the solution is an exponential matrix. We recover the probability

distribution at time t by using the formula:

$$P_t = [e^{-t\mathbb{L}}]^T P_0. \tag{63}$$

Here P_0 is the initial probability distribution vector and P_t is the probability distribution vector at time t. Again using the orthogonality of the eigenvectors, the relation $\mathbb{L} = D^{-1/2}\Phi\Lambda\Phi^T D^{1/2}$ and noticing that the normalized Laplacian matrix is diagonalizable, the matrix exponential is calculated using the eigenvectors as $e^{-t\mathbb{L}} = D^{-1/2}\Phi e^{-t\Lambda}\Phi^T D^{1/2}$. And the spectral decomposition of the exponential matrix at time t is given as:

$$p_t(i,j) = \pi_j + \sum_{k\geq 2} e^{-t\lambda_k}\Phi_k(i)\Phi_k(j)\sqrt{\frac{d_j}{d_i}}. \tag{64}$$

As in the discrete setting, the $(i,j)^{th}$ entry $p_t(i,j)$ evaluates the probability of transition from i to j in time t and the probability tends to π_j in the long time limit as $\mid \lambda_k \mid > 0$. Note that probability conservation holds for both discrete and continuous time random walks, i.e., $\sum_j p_t(i,j) = 1 \; \forall i \in V$ or that the probability of a random walker to jump from state i to any other state is 1.

3.1.3 Heat kernel and diffusion dynamics

The diffusion equation is central to many graph theoretic applications ranging from consensus in multi-agent systems (*OSFM07*) to synchronization in community structure (*ADGPV06*). The diffusion equation models flow from high density regions to low density regions based on the density gradient and is succinctly captured by the equation:

$$\frac{d\Psi}{dt} = -\Delta\Psi. \tag{65}$$

Here Ψ is the quantity that flows (gas, heat, etc.) and Δ is the Laplace operator which governs the rate of flow. On discrete graphs, the Laplace operator takes different forms based on the context of application. It is apparent that if we take $\Delta = \mathbb{L}$, and $\Psi = P_t$, the equation describing the system is the same as equation 62. The solution to the above differential equation also gives rise to the heat kernel (*Chu07*), i.e., the heat kernel H_t is continuous time random walk matrix, p_t. From here on, we refer to the continuous time random walk matrix as the heat kernel, H_t and represent the discrete time analog in terms of the transition matrix M.

Each element of H_t can be represented by expanding the exponential $e^{-\mathbb{L}t}$ using the Maclaurin series of $e^{-t(I-M)}$ as:

$$H_t(u,v) = e^{-\mathbb{L}t}(u,v) = e^{-t}\sum_{k=0}^{\infty} M^k(u,v)\frac{t^k}{k!}. \tag{66}$$

$M^k(u,v)$ is interpreted as the sum of all transfer probabilities of length k joining nodes u and v. t is a real positive number in the range $[0,\infty)$. As the heat kernel H_t is an exponential

sum of random walks, it follows that the derived node-to-node similarities are in some sense a measure of regular equivalence (*LHN06a*). Regularly equivalent nodes serve the same role in a network, which in itself is a reasonable notion of a community (such as functionally related protein complexes or social circles). Furthermore, the exponential sum over paths of all lengths makes the heat kernel robust to noise.

3.2 Diffusion based Laplacian modularity (DLM)

Having discussed the preliminaries, we now focus on using the heat kernel as the connectivity potential matrix in the Laplacian modularity quality function. If we take $S = H_t$, then the resulting Laplacian modularity matrix is $(D - A)H_t + H_t(D - A)$. However, we only consider $B_t = (D - A)H_t$ for analysis as B_t has a precise physical interpretation as the rate of diffusion matrix. In all our subsequent discussions, we term the corresponding quality function, Q_t as the diffusion based Laplacian modularity, abbreviated as DLM. It is worthwhile to note that the random walk Laplacian hints at an alternate strategy to construct the normalized version of the Laplacian modularity quality function, given as:

$$\tilde{Q} = \sum_{i,j}[(I - D^{-1}A)S + S(I - D^{-1}A)]\delta(i, j). \tag{67}$$

Where $I - D^{-1}A$ is the row normalized random walk Laplacian. The column normalized counterpart, $I - AD^{-1}$ can also be used instead of $I - D^{-1}A$ in the above equation. The properties of the Laplacian modularity quality function, Q, such as unbiasedness hold true for the normalized Laplacian modularity quality function, $\tilde{Q}$. If we take $S = H_t$ in the above equation, it can be proved that $(I - D^{-1}A)H_t = H_t(I - D^{-1}A)$ (see Property 2). Hence, the node-to-boundary cohesion matrix, $H_t(I - D^{-1}A)$ contains no additional information. It suffices to only consider the symmetric form $(D - A)H_t$ in the DLM. The definitions of strong and weak communities under the DLM construct are discussed next.

3.2.1 Defining communities using the diffusion based Laplacian modularity

Random walks have been used extensively for community detection (*Alv07, RB08, PL04*), all following the common intuition of a random walker getting trapped in densely connected parts corresponding to communities. We demonstrate that the DLM quality function has a precise probabilistic interpretation in terms of random walks, an alternate viewpoint to the cohesion to subgraph boundaries discussed in the main manuscript. The basic quantity to consider is the probability $p_t(i, j)$ of a random walker to hop from node i to j in time t. This corresponds to the probability after t discrete jumps or Poisson number of jumps with mean t for discrete and continuous time random walks respectively (*Lov93*). Intuitively, a random walker tends to visit nodes in its own subgraph more often than nodes belonging to another subgraph. We formalize this intuition by comparing the frequency of a random walker starting from a node to visit nodes in its own subgraph relative to the frequency of visiting the rest of the nodes at time

t. In order to facilitate pairwise comparison, we consider contributions from random walkers starting from two disjoint sets of internal C_{in} and external C_{out} nodes belonging to the boundary links, i.e., links connecting the subgraph C to the rest of the network. Formally, $H_t^i(C_{in})$ and $H_t^i(C_{out})$ are the sum of probabilities of independent random walks starting from the internal and external boundary nodes of subgraph C to reach node i in time t. Let the set of interfacing links for subgraph C be E_B such that the node pair $[m, n]$ associated with each link satisfy $m \in C, n \notin C$. Note m is an internal node ($m \in C_{in}$) and n is an external node ($n \in C_{out}$). Mathematically, then the two contributions are represented as:

$$H_t^i(C_{in}) = \sum_{(m,n)\in E_B, m\in C} p_t(m,i) \tag{68a}$$

$$H_t^i(C_{out}) = \sum_{(m,n)\in E_B, n\notin C} p_t(n,i). \tag{68b}$$

Intuitively, the two quantities represent the sum of probabilities of two multisets of $|E_B|$ independent random walkers starting from the internal and external boundary nodes to reach the node being evaluated for. Equipped with these definitions, we proceed to define communities in the strong and weak sense.

Community in the strong sense The subgraph C is a community in a strong sense if:

$$H_t^i(C_{in}) > H_t^i(C_{out}), \forall i \in C. \tag{69}$$

In a strong community, the sum of probabilities of the set of random walkers starting from the multiset of internal boundary nodes to visit each node of the subgraph C is greater than the sum of probabilities of the multiset of random walkers starting from external boundary nodes at time t.

Community in the weak sense The subgraph C is a community in a weak sense if:

$$\sum_{i\in C} H_t^i(C_{in}) > \sum_{i\in C} H_t^i(C_{out}). \tag{70}$$

In a weak community, the sum of probabilities of the set of random walkers starting from the multiset of internal boundary nodes of the subgraph C to collectively visit the nodes of subgraph C is greater than the sum of probabilities of the multiset of random walkers starting from the external boundary nodes.

The optimal partition satisfying the definition of community in the weak sense at time t is the DLM quality function:

$$Q_t = \sum_{i,j} [(D - A)H_t]\delta(i,j). \tag{71}$$

Here we represent $(D-A)H_t = B_t$ and term it the DLM matrix. We now prove a few properties of the DLM matrix, B_t.

3.2.2 Properties of the DLM matrix

We use spectral decomposition to prove the symmetry of the DLM matrix.

Property 2. *Although the matrix H_t is asymmetrical in general, the DLM matrix is symmetrical, i.e., for all node pairs (i, j) we have:*

$$B_t(i, j) = B_t(j, i). \tag{72}$$

Proof. **Part a:** We prove the above property using spectral decomposition of the DLM matrix. We rewrite B_t as:

$$\begin{aligned} B_t &= (D - A)H_t \\ &= D(I - M)H_t \\ &= D^{1/2}(I - N)D^{1/2}H_t \\ &= D^{1/2}\mathcal{L}D^{1/2}H_t. \end{aligned} \tag{73}$$

We have used $M = D^{-1}A$ and $N = D^{1/2}MD^{-1/2}$ and $\mathcal{L} = I - N$. H_t is given as $e^{-t\mathbb{L}}$. Substituting the spectral representations we get:

$$\begin{aligned} B_t &= D^{1/2}\mathcal{L}D^{1/2}e^{-t\mathbb{L}} \\ &= D^{1/2}\Phi\Lambda\Phi^T D^{1/2}D^{-1/2}\Phi e^{-t\Lambda}\Phi^T D^{1/2}. \end{aligned} \tag{74}$$

Using the orthogonality of the eigenvectors $\Phi^T\Phi = I$, the final spectral representation for B_t is $D^{1/2}\Phi\Lambda e^{-t\Lambda}\Phi^T D^{1/2}$. Taking the transpose of the derived matrices, we verify that the matrix is symmetrical:

$$\begin{aligned} B_t^T &= (D^{1/2}\Phi\Lambda e^{-t\Lambda}\Phi^T D^{1/2})^T \\ &= (D^{1/2})^T(\Phi^T)^T(e^{-t\Lambda})^T\Lambda^T\Phi^T(D^{1/2})^T \\ &= D^{1/2}\Phi e^{-t\Lambda}\Lambda\Phi^T D^{1/2} \\ &= B_t. \end{aligned} \tag{75}$$

Note, D and $e^{-t\Lambda}, \Lambda$ are diagonal matrices. Symmetry implies the row sums, like the column sums of B_t are all 0.

Part b: We now prove the above property for discrete random walks where the similarity matrix at time t is given as M^t.

$$\begin{aligned} B_t^{discrete} &= D^{1/2}\mathcal{L}D^{1/2}M^t \\ &= D^{1/2}\Phi\Lambda\Phi^T D^{1/2}D^{-1/2}\Phi\Lambda'^t\Phi^T D^{1/2}. \end{aligned} \tag{76}$$

The spectral representation for $B_t^{discrete}$ is $D^{1/2}\Phi\Lambda(I-\Lambda)^t\Phi^T D^{1/2}$ and we have:

$$\begin{aligned}
(B_t^{discrete})^T &= (D^{1/2}\Phi\Lambda(1-\Lambda)^t\Phi^T D^{1/2})^T \\
&= (D^{1/2})^T(\Phi^T)^T((1-\Lambda)^t)^T\Lambda^T\Phi^T(D^{1/2})^T \\
&= D^{1/2}\Phi(I-\Lambda)^t\Lambda\Phi^T D^{1/2} \\
&= B_t^{discrete}.
\end{aligned} \tag{77}$$

Part c: The spectral decomposition can also be used to prove $(I-D^{-1}A)H_t = H_t(I-D^{-1}A)$ as follows:

$$\begin{aligned}
(I-D^{-1}A)H_t &= D^{-1/2}(I-N)D^{1/2}H_t \\
&= D^{-1/2}\mathcal{L}D^{1/2}e^{-t\mathbb{L}} \\
&= D^{-1/2}\Phi\Lambda\Phi^T D^{1/2}D^{-1/2}\Phi e^{-t\Lambda}\Phi^T D^{1/2} \\
&= D^{-1/2}\Phi\Lambda e^{-t\Lambda}\Phi^T D^{1/2}.
\end{aligned} \tag{78}$$

$$\begin{aligned}
H_t(I-D^{-1}A) &= H_t D^{-1/2}(I-N)D^{1/2} \\
&= e^{-t\mathbb{L}}D^{-1/2}\mathcal{L}D^{1/2} \\
&= D^{-1/2}\Phi e^{-t\Lambda}\Phi^T D^{1/2}D^{-1/2}\Phi\Lambda\Phi^T D^{1/2} \\
&= D^{-1/2}\Phi\Lambda e^{-t\Lambda}\Phi^T D^{1/2}.
\end{aligned} \tag{79}$$

□

When S is the heat kernel, H_t or powers of the transition matrix, M^t, the DLM matrix like the modularity matrix has row and column sums equal to zero.

3.2.3 Connection of DLM to the rate of diffusion

We showed that the DLM matrix is a scaled derivative of the diffusion matrix, i.e., $(D-A)H_t$ is $-D$ times the derivative of $e^{-t\mathbb{L}}$ in the main manuscript. This interpretation enables us to make an implicit connection with diffusion which underscores the dynamics governing community exploration. The physical analogy of the diffusion equation is that a commodity (heat, current) flows from a high density region to a low density region until equilibrium and flow rate is dependent on the negative of the density gradient (*New10*). Consider a network with $|V|$ nodes on which we independently administer a substance of d_i units to node i at time $t=0$ and the ground the rest of the nodes. We do this sequentially for all nodes and observe the dynamics of the diffusion process over time. Then the matrix $-(D-A)e^{-t\mathbb{L}}$ precisely encapsulates the information about the rate of flow for all node pairs of this physical process. Tailoring equation 63 so as to account for the amount of substance administered at each node, we get:

$$\begin{aligned}
H_t &= [e^{-t\mathbb{L}}]^T D H_0 \\
&= [e^{-t\mathbb{L}}]^T D^T H_0 \\
&= [De^{-t\mathbb{L}}]^T H_0.
\end{aligned} \tag{80}$$

Differentiating, we have:

$$\frac{dH_t}{dt} = [-D\mathbb{L}e^{-t\mathbb{L}}]^T H_0. \tag{81}$$

As time grows tightly connected nodes reach a local equilibrium with and long scale flow comes into play (*She13*). Hence, the intuition behind using the rate of diffusion for finding community structure is to minimize the rate of flow $\frac{dH_t}{dt}$ within well connected groups or equivalently maximize the negative of the diffusion flux matrix $D\mathbb{L}e^{-t\mathbb{L}}$, where this flux flows through the community boundary links. The property of the all columns of B_t summing to zero physically follows from flow conservation in diffusion systems.

3.2.4 Connection of DLM to multiscale modularity

We discussed that the Laplacian modularity quality function is closely related to the modularity in the previous section. The diffusion based Laplacian modularity is also closely related to the multiscale modularity (*LDB14*). The multiscale modularity using the heat kernel, $\breve{Q}_t$ for undirected, unweighted networks at time t can be written as:

$$\breve{Q}_t = \frac{D}{2|E|}\sum_{i,j}[e^{-t\mathbb{L}} - \mathbb{1}\pi^T]\delta(i,j). \tag{82}$$

Here $\mathbb{1}$ is a $|V|$ length row vector of ones, π is the vector of stationary distribution and $|E|$ is the number of links in the network. Let us consider the multiscale modularity matrix, $\breve{B}_t = D(e^{-t\mathbb{L}} - \mathbb{1}\pi^T)$. Differentiating, $\breve{B}_t$ with respect to t, we get:

$$\begin{aligned}
\frac{d\breve{B}_t}{dt} &= D\frac{d(e^{-t\mathbb{L}} - \mathbb{1}\pi^T)}{dt} \\
&= D\frac{d[e^{-t\mathbb{L}}]}{dt} - D\frac{d[\mathbb{1}\pi^T]}{dt} \\
&= -D\mathbb{L}e^{-t\mathbb{L}} + 0 \\
&= -(D-A)H_t \\
&= -B_t.
\end{aligned} \tag{83}$$

Hence, the DLM matrix is the negative differential of the multiscale scale modularity matrix using the heat kernel. This connection establishes a relationship between our stability curve and the stability curve of (*LDB14*), discussed in section 3.4

3.3 Identifying overlapping nodes using DLM

Similar to the Laplacian modularity quality function discussed in the previous section, the $K\times \mid V \mid$ matrix $X^T B_t$ can efficiently identify communities in the strong sense by checking for positive entries, and hence, identify overlapping nodes for DLM at any time t.

A distinctive feature of our approach to determine overlap in networks using DLM is that it progressively considers contribution from paths of all lengths in contrast to a local edge density evaluation in (*LRRF, ABL10*). This has the desirable effect of uncovering overlapping nodes dynamically with time in contrast to all other approaches. In the previous section we defined overlapping nodes as those which individually satisfy the strong criterion of a community. In order to gain an intuition of the dynamic process revealing overlap in the DLM approach, we provide an alternate viewpoint in terms of path length distribution. Consider each element of DLM matrix for continuous time random walks expressed in terms of path length probabilities:

$$\begin{aligned} B_t(i,j) &= [D-A]H_t \\ &= D(I-M)e^{-t\mathbb{L}} \\ &= D(I-M)[e^{-t}\sum_{k=0}^{\infty} M^k(i,j)\frac{(t)^k}{k!}] \\ &= De^{-t}\left(\sum_{k=1}^{\infty}[\frac{(t)^k}{k!} - \frac{(t)^{k-1}}{(k-1)!}]M^k(i,j) + I\right) \\ &= \sum_{k=1}^{\infty} f_{ij}(t,k)M^k(i,j). \end{aligned} \tag{84}$$

Here each element of$e^{-t\Delta_{\alpha M}}$ is expanded using Maclaurin series expansion of $e^{-t(I-M)}$ to get $B_t(i,j)$ in terms of $M(i,j)$. $f_{ij}(t,k)$ is a function of time t which absorbs the constants and the final expression validates that each term of $B_t(i,j)$ is a combination of all path lengths from 1 to ∞. The membership of a node j to subgraph C is decided by the value of $\sum_{i\in V^k} B_t(i,j)$ which again is a dynamic function of time:

$$\sum_{i\in V^k} B_t(i,j) = \sum_{i\in V^k}\sum_{k=1}^{\infty} f_{ij}(t,k)M^k(i,j). \tag{85}$$

Consequently, overlap emerges dynamically based on probabilistic paths of all length from nodes in V_k to node j, a feature unique to our method. As we consider a weighted sum over all possible path lengths instead of shortest path length, overlap determination is robust to small topological changes in the network. This is desirable when the network has spurious or missing links as common in many biological networks. We will discuss the strategy we adopt to identify overlap using a threshold parameter in section 7.3.

3.4 Identifying hierarchy using DLM

We mentioned some strategies to extend Laplacian modularity using a resolution parameter in the last section. However, a common problem in all these methods was that there was no principled way to determine the relevant partitions from the set of all partitions obtained by

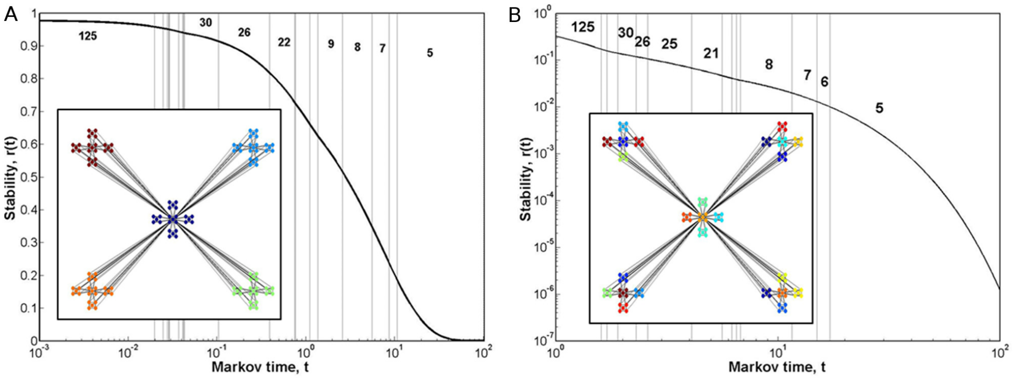

Figure 9: Comparing stability curves. *A: Stability curve using clustered autocovariance (DYB10) for a hierarchical, scale-free graph proposed in (RB03) with 125 nodes. The curve identifies the natural 5-way partition (inset) but fails to identify the finer 25-way partition for all values of the resolution parameter* t*. The 26-way partition which is identical to the 25-way partition with 5 nodes in all but one community, in which the central node is assigned to an independent community. The stability value for this 26-way partition exceeds that of the 25-way partition for all* t*, and hence, the autocovariance stability function fails to identify the relevant hierarchical organization of the network. B: Our stability function identifies the 25-way partition (inset) along with the 5-way partition to be the persistent over relatively longer time spans.*

varying the resolution parameter. We call these relevant partitions as hierarchy or the topological scales of the network. DLM too is a multi-resolution extension of the Laplacian modularity as there is a parameter t which controls the scale of analysis. In order to identify hierarchy, it is important to determine which partitions are relevant over the time range $t = [0, \infty]$. In the main text, we introduce the stability curve $r(t)$ to determine persistent partitions over range of analysis. It is given by:

$$r(t) = max_X trace[R_t]. \tag{86}$$

Where R_t is the clustered matrix for partition X given by $R_t = \frac{1}{2|E|}X^T[(D-A)H_t]X$. Although our stability function shares several features of the stability function described in (*DYB10*), the dynamics governing the clustering process are fundamentally different. Specifically, our stability curve is the derivative of the stability curve of (*LDB14*) as described in equation 83. We now state the similarity between these stability functions and describe the differences in Section 3.6.

3.4.1 Properties of the stability function

The similarity between our stability curve and the stability curves of (*DYB10, LDB14*) is that all these stability curves organize a hierarchy of fine to coarse communities as time increases, with the trivial $|V|-$way partition at time $t = 0$ and 2-way partition at time $t = \infty$.

Property 3. *Let B_t represent the DLM matrix, $(D - A)H_t$. The associated quality function Q_t partitions the graph into two communities in the limit $t \to \infty$ and places each node in a separate community for $t = 0$.*

All nodes are placed in individual communities, if and only if merging two nodes leads to an inferior quality of partition. Hence for the case $t = 0$, it suffices to prove that all non-diagonal elements are negative, i.e., the number of communities K is $|V|$, *iff* $[D - A]H_0(i,j) \leq 0$, $\forall i \neq j$. H_t is equal to the identity matrix I at time $t = 0$ for both continuous and discrete time random walk. Hence, $B_0 = [D - A]$. As D is a diagonal matrix with positive entries and $[D - A]$ considers the negative of the adjacency matrix A with all positive entries, it is clear that the only positive entries in $[D - A]$ lie on its trace. Hence, all non-diagonal entries are negative leading to $K = |V|$. We resort to the spectral decomposition of B_t in order to show Q_t outputs a 2-partition as $t \to \infty$. As $t \to \infty$, barring degenerate eigenvalues, the DLM matrix is dominated by the normalized Fielder vector given by:

$$\begin{aligned}
\lim_{t\to\infty} B_t(i,j) &= \lim_{t\to\infty}[D - A]H_{t\to\infty}(i,j) \\
&= \lim_{t\to\infty} D(I - M)e^{-t\Delta_M} \\
&= \lim_{t\to\infty} D(D^{-1/2}\Phi\Lambda\Phi^T D^{1/2})(D^{-1/2}\Phi e^{-t\Lambda}\Phi^T D^{1/2}) \\
&= \lim_{t\to\infty} D^{1/2}\Phi\Lambda e^{-t\Lambda}\Phi^T D^{1/2} \\
&\approx D^{1/2}\Phi_2(\lambda_2 e^{-t\lambda_2})\Phi_2^T D^{1/2} \\
&= \sqrt{d_i d_j}\lambda_2 e^{-t\lambda_2}\Phi_{2,i}\Phi_{2,j}.
\end{aligned} \tag{87}$$

The approximation of $D^{1/2}\Phi\Lambda e^{-t\Lambda}\Phi^T D^{1/2}$ is $D^{1/2}\Phi_2(\lambda_2 e^{-t\lambda_2})\Phi_2^T D^{1/2}$ at long times because the eigenvalue λ_2 dominates all eigenvalues asymptotically, including λ_1 ($\lambda_i e^{-t\lambda_i} = 0$ for $\lambda_1 = 0$). Let $r(t, X)$ be the stability curve for partition X. Now, consider the finest partition into $|V|$ communities with X_f equal to an identity matrix of size $|V|$. Then the asymptotic value of stability curve for X_f as $t \to \infty$ is given by:

$$r(t, X_f) \approx \frac{\lambda_2 e^{-t\lambda_2}}{2 \mid E \mid} \sum_i d_i \Phi_{2,i}^2. \tag{88}$$

The value for the partition X_{nf} where we merge 2 nodes i and j into a single community and assign the rest of the nodes independent communities is:

$$r(t, X_{nf}) \approx \frac{\lambda_2 e^{-t\lambda_2}}{2 \mid E \mid} \big(\sum_{k\neq i,j} d_k \Phi_{2,k}^2 + (\sqrt{d_i}\Phi_{2,i} + \sqrt{d_j}\Phi_{2,j})^2\big). \tag{89}$$

The difference of these values is:

$$r(t, X_{nf}) - r(t, X_f) \approx \frac{\lambda_2 e^{-t\lambda_2}}{\mid E \mid} \sqrt{d_i d_j}\Phi_{2,i}\Phi_{2,j}. \tag{90}$$

Which only increases if $sign[\Phi_{2,i}] = sign[\Phi_{2,j}]$ as $\Phi_{2,i}\Phi_{2,j}$ becomes positive. Inductively, the quality function $\tilde{Q}$ is maximized by two-way partition based on signs of Fielder vector (eigenvector corresponding to λ_2) as $t \to \infty$. Although, we proved the property for continuous walks, the proof for discrete walks is analogous.

An important property of the DLM matrix is the monotonicity of the diagonal terms. This enables us to analyze stability without resorting to the minimum over time as in the stability formalism of (*DYB10*). The monotonicity of the diagonal terms $B_t(i,i)$ follows from spectral decomposition:

$$\begin{aligned} B_t(i,j) &= \sqrt{d_i d_j} \sum_{k \geq 2} \lambda_k e^{-t\lambda_k} \Phi_{k,i} \Phi_{k,j} \\ B_t(i,i) &= d_i \sum_{k \geq 2} \lambda_k e^{-t\lambda_k} \Phi_{k,i}^2. \end{aligned} \tag{91}$$

We have $\Phi_{k,i}^2 > 0$ and $\lambda_k e^{-t\lambda_k} > 0$ and in addition $\lambda_k e^{-t\lambda_k}$ is monotonic over time for $0 < \lambda_k < 2$. Hence, the diagonal terms decrease over time and diagonal becomes the zero vector as $t \to \infty$. The monotonicity directly implies that Q_t is maximum at time, $t = 0$ with magnitude 1 (the trace of the clustered matrix $X_f^T D - AX_f = D - A$ is equal to $2|E|$).

A compelling validation of our approach to identify hierarchy is displayed in Figure 9. Our stability curve for a hierarchical (Figure 9A), scale-free graph (*RB03*) with 125 nodes identifies the 25-way partition along with the 5-way partition to be the persistent over relatively long time spans. Figure 9B shows the stability curve for the clustered autocovariance (*DYB10*), a generalization of the modularity to identify topological scales, optimized over entire time range. We observe that it identifies the natural 5-way partition but fails to identify the finer 25-way partition for all values of the resolution parameter t. Although, the authors in (*DYB10*) state that they identify the 25-way partition to be stable according to their method, we contend that it is due to the modularity optimization technique used to evaluate the stability curve. The 26-way partition with the center node assigned to an independent community always has a higher stability value, $r(t)$ according to the clustered autocovariance than the 25-way partition over the time range [0.1,1]. This is independent of the choice of modularity optimization.

3.4.2 Stability on random graphs

As mentioned previously, our stability curve is the continuous time derivative of the stability curves of (*DYB10, LDB14*). Hence the analysis of the stability curve $r(t)$ mirrors the analysis of (*DYB10*) on random networks such as the Erdős-Rényi random graphs. As argued in (*DYB10*), the stability value $r(t)$ can used to distinguish between random graphs and graphs with community structure. In random graphs, the stability of all detected partitions will decay quickly whereas the stability value for a partition corresponding to a valid community structure of a network will have relatively higher values. We demonstrate this phenomenon by comparing the stability curves of a random network generated by the Erdős-Rényi model with no community structure against a real-world network and a synthetic network with community structure

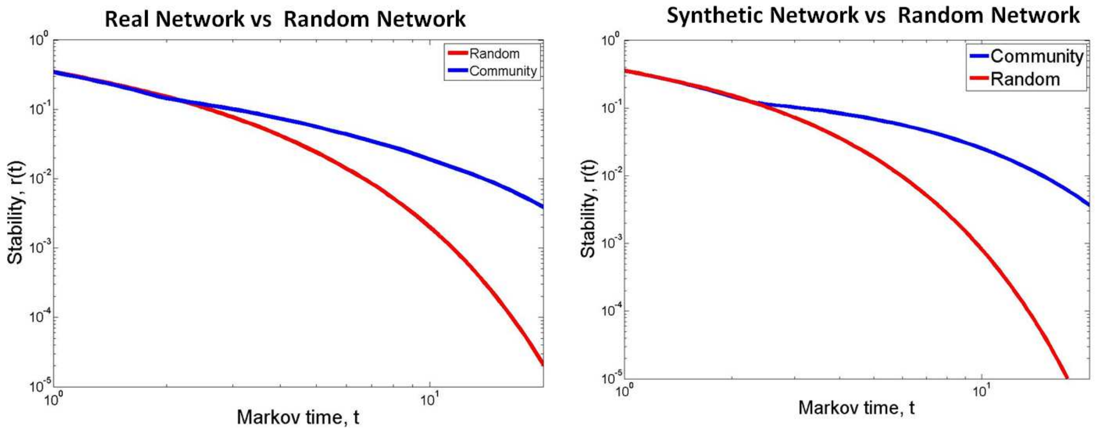

Figure 10: Comparing stability curves of networks with communities and random networks. *Left: Stability plots of the football network (blue) and of an Erdős-Rényi random graph (red) of same size and average degree. Right: Stability plots of a synthetic network of 1000 nodes with a planted community structure (blue) and of an Erdős-Rényi random graph (red) of same size and average degree.*

in Figure 10. We compare the stability plot of the football network (blue) and the stability curve of an Erdős-Rényi random graph (red) of same size and average degree on the left, and compare the stability plot of a synthetic network of 1000 nodes with a planted community structure (blue) and the stability curve of a Erdős-Rényi random graph (red) of same size and average degree on the right. In both cases, we see that the stability value of all partitions identified by our method on random networks quickly converges to zero, whereas the stability values over time for networks with community structure are higher, especially at larger values of time t. In the next section, we provide a parameter-free algorithm to select a time range of analysis for the DLM. The choice of a time range enables the algorithm to neglect the two-way partition when irrelevant.

3.5 Automatic community identification

The thrust of our framework to identify communities is based on the assumption that there is no best partition in real world networks but several valid community assignments which we discern using the parametric stability curve, the parameter being time, t. In the main body of the paper, we establish that partitions optimal over long time windows of the stability curve correspond to the set of relevant partitions of the network. However, for fair comparison to other parameter-free community detection methods, we provide a method to obtain the most relevant or best partition from the stability curve in the continuous time case. This partition is used to compare our approach to the other methods in the main manuscript.

3.5.1 Identifying optimal partition

For the purpose of constructing the stability curve $r(t)$, we first discretize t over the range $[0, t_{max}]$ into say τ distinct values. We run Louvain for each value of t and obtain a set of τ partitions. We extract the set of unique partitions from these τ partitions as some (persistent) partitions naturally occur more than once. We draw a stability curve $r(t, X)$ for each unique partition and the optimal stability curve follows equation 86. A naive analysis of this stability curve based on length of persistent time window of partitions in the stability curve will always output the 2-way partition to be most relevant as it persists over the time range $[t_2, \infty]$ where t_2 is the smallest time value for which the 2-partition becomes optimal. Hence, it is imperative to discern whether the 2-way partition is a sound partition of the network in a practical context or an artifact of the stability curve. For this purpose, we explore our network over a limited time horizon $[t_{min}, t_{max}]$ with the expectation that communities identified at $t < t_{min}$ and $t > t_{max}$ are second fiddle to the overall network analysis. Recall that at $t = 0$, the number of identified communities is the trivial $|V|$-way partition, assigning each node to its own community. This partition is ignored by setting t_{min} to be equal to the smallest value of t for which the number of identified communities is less that $|V|$. Naively fixing t_{max} to a constant value without regard to graph topology could lead to missing vital information present in the analysis beyond t_{max}. Hence, it is reasonable to assert that the time t_{max} should be dependent on a global property of the graph and guarantee random walkers have explored a *sufficient* horizon of the graph. There exists several possible choices, but a heuristic that works well in practice is to take t_{max} to be equal to the relaxation time of the Markov chain or when the Markov chain is close to the steady state distribution. Mathematically, the relaxation time is the inverse of the asymptotic rate of convergence to the stationary distribution and equal to the inverse of the spectral gap, $1/\lambda_2$ for continuous time chains and $1/(\lambda'_2)$ for discrete time chains (*Lov96*). Thus, we first discretize t with logarithmic spacing in the time range $[t_{min}, t_{max}]$ and then run the Louvain algorithm on the corresponding DLM matrices for these values of t. We set the number of discrete values of t to be 200 for all networks. Next, the partitions are used to construct the stability curve and subsequently ranked based on the length of the persistent time window. The length of time window is set to be equal to the difference of $log(t_{end})$ and $log(t_{int})$ where t_{int} and t_{end} are the start and end values of the time t over which a given partition is optimal. We choose the logarithmic time scale because the DLM matrix at time t is an exponential function of t and the logarithm is the reciprocal function of exponentials, i.e., the exponentially weighted time t scales linearly on a logarithmic scale for comparison between partitions. The partition with maximum $log(t_{end}) - log(t_{int})$ value is chosen to be the most relevant hierarchical partition.

3.5.2 Identifying overlapping nodes in best partition

After extracting the hard maximally persistent partition over the time window $[t_{int}, t_{end}]$ in the stability curve, we obtain overlapping communities at each discrete value of t falling in the interval $[t_{int}, t_{end}]$ using the method described in Section 2.6. These overlapping partitions obtained for different values of $t \in [t_{int}, t_{end}]$ may not be mutually consistent, as overlap may

dynamically emerge or disappear based on path length distribution over the time interval. We resolve this conflict using the magnitude of $X_o^T B_t(k,i)$ as it is indicative of the strength of association of an overlapping node i with the subgraph C^k it overlaps with, where X_o is the indicator matrix for an overlapping partition. $X_o^T B_t(k,i)$ is also the magnitude of connectivity flux (difference of internal and external boundary cohesion)of node i flowing the boundary links of the subgraph C^k wherein some nodes are overlapping. Hence, the positive values indicate the relative membership strength of an overlapping node with its corresponding subgraph, i.e., leads to *fuzzy* overlap. We evaluate the fuzzy overlap of each overlapping node in the range $t \in [t_{int}, t_{end}]$. This dynamically varying fuzzy overlap is transcribed to hard overlap by thresholding the normalized magnitude of association as follows:

$$(i \in C^u) \iff (\frac{X_o^T B_t(u,i)}{X_o^T B_t(k,i)} \geq \epsilon) \forall t \in [t_{int}, t_{end}]. \tag{92}$$

Where ϵ is a threshold parameter which discerns the minimum strength of relative association of a node i with subgraph V^u (normalized by association strength with the subgraph C, node i was assigned in the hard partition) necessary for *hard* overlap. It is easy to verify that $\epsilon = 1$ recovers the hard partition as $\frac{X_o^T B_t(u,i)}{X_o^T B_t(k,i)}$ is 1 only for $u = k, \forall t \in [t_{int}, t_{end}]$. Note that we can also use X instead of X_o in the above equations, with the difference being the inclusion or exclusion of overlapping nodes in the determination of fuzzy overlap membership strength.

Although we do not identify background nodes in this manuscript, we note that the ideas for detecting overlapping nodes can be extended to identify background nodes as done in (*LRRF*). Specifically, the connectivity flux of a background node across the community boundary will be relatively lower than the core community nodes. These flux values are encoded as $X^T B_t(u,i)$ for a subgraph C^u and nodes $i \in C^u$, and comparing these values can lead to identifying background nodes.

3.5.3 Parameter-free approach for community detection

The steps for identifying the most relevant communities in a network are as follows:

- Discretize time t over time range $[t_{min}, t_{max}]$, where t_{min} is the value of time t such that the number of identified communities is less than number of nodes in the network and $t_{max} = 1/(\lambda_2')$.
- Run the Louvain algorithm on the DLM matrices corresponding to the discretized values of t and obtain a set of partitions.
- Calculate the stability values, and hence, the stability curve for the network using the set of partitions.
- Extract the most relevant partition from set of partitions optimal over different time windows using the length of persistence, i.e., partition with maximum $log(t_{end}) - log(t_{int})$ value.

- Find the overlapping nodes for a given threshold value ϵ using equation 92

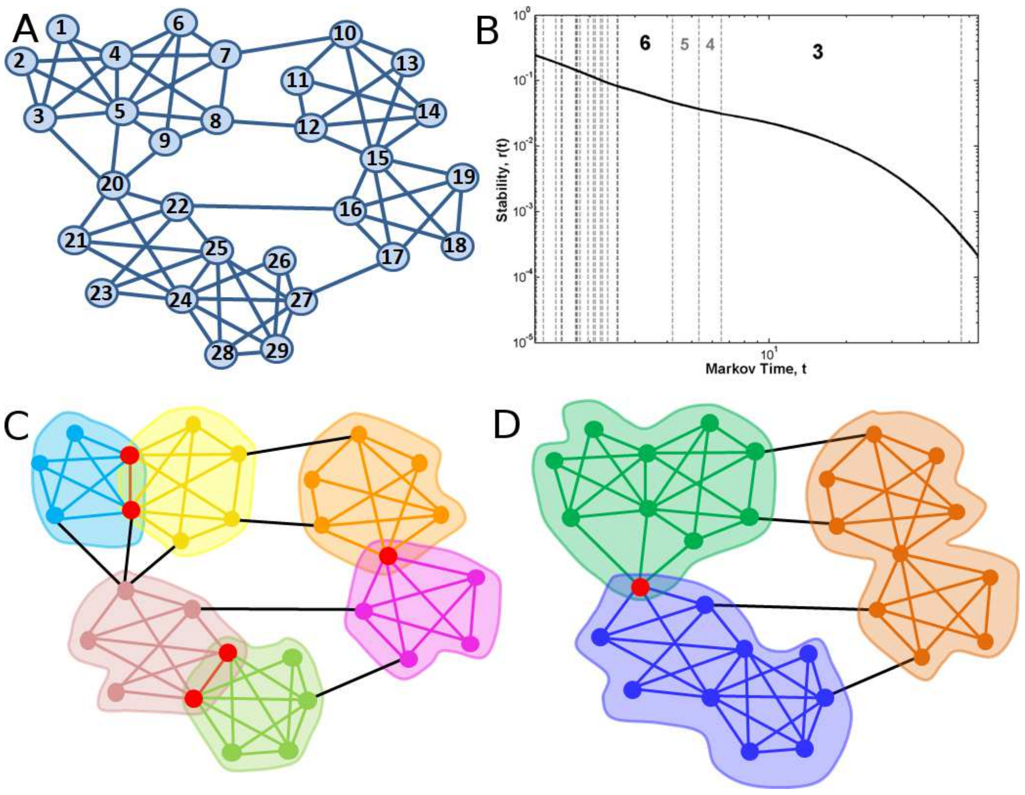

Figure 11: Community detection using DLM. *A: Network of 29 nodes. B: Stability curve as derived by DLM. C: 6 identified communities with overlapping nodes highlighted in red at threshold* $\epsilon = 0.5$. *D: 3 identified communities with overlapping nodes in red at threshold* $\epsilon = 0.5$.

Figure 11 demonstrates DLM on a synthetic network of 29 nodes as shown in the main manuscript. The stability curve for the network in figure 11 B shows two persistent basins enclosed within dotted lines corresponding to the 3-way partition and 6-way partition. The 3 and 6 communities along with the corresponding overlap at $\epsilon = 0.5$ are shown in Figure 11 C,D. We next discuss the computational complexity of our method and provide approximate techniques for constructing the DLM matrix for very large graphs.

3.5.4 Computational complexity and approximation

We analyze the complexity of constructing the DLM matrix. In order to calculate the B_t exactly, we need to find H_t. For discrete time random walks, H_t is directly computable in $\mathcal{O}(cEt)$, or estimated in $\mathcal{O}(Kt)$ with accuracy $\mathcal{O}(c/\sqrt{K})$ through K random walks of length t (***PL04***). For continuous time walks, calculating matrix exponential $e^{-t\mathbb{L}}$ unfortunately has complexity

$\mathcal{O}(|V|^3)$. Instead of recalculating $e^{-t\mathbb{L}}$ for different values of t, we decompose the normalized Laplacian $\mathcal{L}$ into its eigenvalues and eigenvectors. The eigenvectors serve as a common orthonormal basis, and hence, $e^{-t\mathbb{L}}$ is efficiently calculated by changing the weighting function of the eigenvalue matrix, i.e., $e^{-t\Lambda}$ in $D^{-1/2}\Phi e^{-t\Lambda}\Phi^T D^{1/2}$. Calculating the full spectral decomposition of the Laplacian has complexity $\mathcal{O}(|V|^3)$. However, as most real world networks are sparse, complexity reduces to $\mathcal{O}(|V|^2)$ using the efficient Lanczos method (*QH07*). It is approximated in $\mathcal{O}(k|V|)$ by calculating the first (smallest) k eigenvalues and corresponding eigenvectors. The error bound is (generally) linear in $1/k$ (*Rus11*) due to the decreasing smoothness property of the eigenvectors. An alternative approach to speed up calculations is to use the technique proposed in (*KG13*) to sequentially compute each column of the exponential matrix with sublinear time complexity. Eigen decomposition is prohibitive for very large graphs, and hence, discrete random walks are more suitable for large networks. The second part to our method is optimization using Louvain for τ distinct values of t. Although the exact computational complexity of the method is not known, the method seems to run in time $O(|V|log|V|)$ with most of the computational effort spent on the optimization at the first level (*BGLL08*). We demonstrate the applicability of DLM to very large networks using the sparse random walk transition matrix M as S in Section 8.

3.6 Analysing the resolution limit for DLM

We analyze the resolution limit of DLM for S equal to the heat kernel, H_t as well as S equal to the transition matrix M in terms of the horizon of interacting node pairs in the network.

3.6.1 Analysing the resolution limit in DLM for $S = M$

Modularity compares the number the edges inside a community relative to the number of edges inside a community expected at random. This comparison is expressed succinctly using our representation as $DM > DM^{\infty}$ where $M^{\infty} = \mathbb{1}\pi^T$ represents the matrix of stationarity distributions and multiplying by D preserves the degree distribution and volume of the graph while randomly rewiring the edges. The quality function $DM - DM^{\infty}$ aims to maximize edges inside a module relative to that expected at random. The resolution limit of modularity (*FB07*) stems from implicitly assuming all node pairs interact with each other in the null model. On the contrary, our notion of a community using discrete time Markov process translates to $(D-A)M^t > 0$ or $DM^t > DM^{t+1}$ and unlike modularity, we only consider node pairs at resolution $t+1$ to be interacting while resolving the community structure at resolution t.

Our representation to quantify interacting node pairs also provides a mechanism to define null models conditioned on a horizon of interaction, i.e., DM^t wires edges based on random paths of length t or less emanating from each node. Such a formulation of nulls models based on path lengths instead of degrees, has remained elusive in community detection literature (*For10*), and we contend to be an important step towards precise definition of nulls models. It also provides an alternate approach to reduce the traditional resolution limit of modularity optimization.

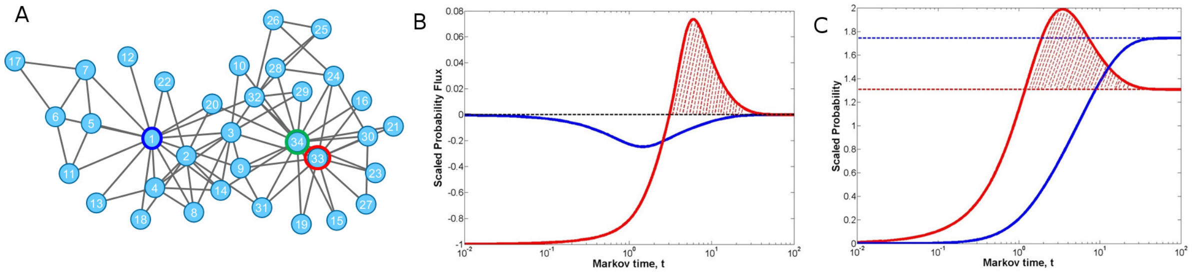

Figure 12: Comparing diffusion dynamics and rate of diffusion dynamics. *A: Karate network B: The scaled rate of diffusion dynamics over time for two pairs of nodes. The the rate of diffusion between node pair [34,33] is shown by red curve. The the rate of diffusion between node pair [34,1] is shown by blue curve. Two nodes are considered for coarse graining only if the probability flux is positive. The probability flux between pair [34,33] is positive for some time t (shown by shaded red region), whereas the probability flux between pair [34,1] is never positive. C: The diffusion dynamics over time for same pairs of nodes. The horizontal dotted lines indicate the baseline value for the node pairs in the null model. Two nodes are considered for coarse graining only if the probability over time exceeds the baseline value. The probability flux between pair [34,33] is positive for some time t (shown by shaded red region), whereas the probability flux between pair [34,1] is never positive. It follows from elementary calculus that the time at which probability value for node pair [34,33] is maximum (red curve) corresponds to the time at which the probability flux value for node pair [34,33] is 0 in B. Hence, the gain by coarse graining two nodes is dependent on extrinsic null model for diffusion dynamics, whereas is dependent on intrinsic network topology for rate of diffusion dynamics.*

We validated in section 2.6 that when S is local, the Laplacian modularity does not suffer from the traditional resolution limit of modularity optimization. The transition matrix M is local and also will not suffer from the traditional resolution limit of modularity in the Laplacian modularity framework. Guided by the intuition that DM^t restricts the horizon of interaction, we can instead define modularity as $DM > DM^t$ with some t less than or equal to the diameter of the graph. In contrast to ad-hoc resolution parameters weighing standard configuration null model, DM^t progressively restricts the horizon of interacting node pairs, and smaller choices of t will have progressively lower resolution limit than modularity, with $t = 1$ having the least resolution limit which corresponds to Laplacian modularity quality function for $S = M$. We contend that modularity defined as $DM > DM^t$ will have a lower resolution limit that the definition of modularity as $DM^t > DM^\infty$ in (***DYB10***) as the former does a comparison over a local neighborhood of interaction, whereas the latter compares interactions of random walkers at time t to the configuration null model or equivalently stationary distribution matrix DM^∞ which is global in nature.

3.6.2 Analysing the resolution limit in DLM for $S = H_t$

The horizon of node interactions is further suppressed in the continuous time random walks or for the heat kernel H_t as S because node pairs at resolution $t + dt$ implicitly interact to resolve the community structure at resolution t, where dt is an infinitesimally small period of time. This can be verified by considering the DLM matrix $[D - A]e^{-t\mathbb{L}}$ and rewriting it as:

$$\begin{aligned}
[D - A]e^{-t\mathbb{L}} &= D\mathbb{L}e^{-t\mathbb{L}} \\
&= \frac{D\mathbb{L}e^{-t\mathbb{L}}dt}{dt} \\
&= \lim_{dt\to 0} \frac{D\mathbb{L}e^{-t\mathbb{L}}dt}{dt} \\
&= \lim_{dt\to 0} \frac{D(e^{-t\mathbb{L}} - e^{-(t+dt)\mathbb{L}})}{dt}.
\end{aligned} \tag{93}$$

We have used the Maclaurin series expansion to rewrite $\mathbb{L}e^{-t\mathbb{L}}dt$ as $(e^{-t\mathbb{L}} - e^{-(t+dt)\mathbb{L}})$. $e^{-t\mathbb{L}}$ corresponds to M^t and $e^{-(t+dt)\mathbb{L}}$ corresponds to M^{t+1} for the DLM matrix with $S = M^t$.

Considering the rate of diffusion dynamics for reducing the resolution limit has a decisive advantage over multiscale modularity using the heat kernel (*LDB14*) and expected diffusion dynamics in general. This is highlighted by investigating the question: At what specific instant of time do the two dynamic approaches coarse-grain a pair of nodes into one? For diffusion dynamics as in (*LDB14*), it is equivalent to choosing a choosing a sufficiently large t such that $d_iH_t(i,j) > d_iH_\infty(i,j)$, i.e., the scaled probability of a random walker to reach node j starting from i after t steps exceeds its stationary probability scaled by d_i. This is because $d_iH_\infty(i,j) = 2|E|\pi_i\pi_j$ for undirected networks, i.e., the $(i,j)^{th}$ element of the configuration null model. For rate of diffusion dynamics, we choose t such that $d_i\frac{-dH_t(i,j)}{dt} > 0$ or equivalently any value of time t after the probability curve $H_t(i,j)$ has reached a maximum (the derivative is 0 at the peak). This also follows from equation 83. Using the Kolmogorov's backward equation, $d_i\frac{-dH_t(i,j)}{dt}$ can also be expressed as $d_jH_t(i,j) - \sum_{k\in N(j)} H_t(i,k)$ where $N(j)$ indicates the 1-neighborhood of node j. It follows from Markov chain theory that the gain by coarse graining a pair of nodes is positive if the probability of a random walker to reach node j after t steps from node i exceeds the average probability of reaching node j's neighbours, $H_t(i,j) > \frac{\sum_{k\in N(j)} H_t(i,k)}{d_j}$. Thus, the decision to coarse grain is independent of the stationary distribution but instead dependent on the collective dynamics at the same time resolution over the 1-neighborhood (see Figure 12). At the network level, this translates into exploring community structure by performing a local comparison of probability measures where the precise formalism of local evolves dynamically with time. This is in stark contrast to expected diffusion dynamics of (*LDB14*) which resolves communities by analyzing the local to global probability measures against the global stationary distribution, and hence, invariably involves a global comparison.

4 Extending Laplacian modularity to other network types

In this section, we extend the Laplacian modularity framework to other network types encountered in network science analysis like weighted, directed, signed and multiplex networks.

4.1 Weighted Networks

We denote a weighted graph by the triple $G = (V, E, A)$ where V is the set of nodes, $E \subseteq V \times V$ is the set of edges and A is the weighted adjacency matrix:

$$A(i,j) = \begin{cases} w(i,j) & \text{if} \quad (i,j) \in E \\ 0 & \text{otherwise.} \end{cases} \tag{94}$$

Here $w(i,j)$ is the weight on the edge $(i,j) \in E$. Let $D = diag(d^i_{wgt})$ be the diagonal weighted degree matrix with elements given by the degree of the nodes $d^i_{wgt} = \sum_j w(i,j)$. The weighted Laplacian matrix takes the same form as in the unweighted case, i.e., $L = D - A$. The definition of strong and weak communities follow those of the unweighted graph, except that the membership vector includes the weights of the induced links, i.e., in the equation:

$$S(V) = S^T \mathcal{V} + S\mathcal{V}. \tag{95}$$

Here $\mathcal{V}$ is the membership vector, where the element $\mathcal{V}^i$ is equal to the sum of edge weights node i is connected to, instead of the cardinality as in unweighted graphs. If we have a weighted network, then the weights indicate the capacity of flow through each link. Our formulation of flux flowing through a boundary link then modifies to a product of the flux flowing through the boundary link for the corresponding unweighted network times the weight of the link. We write weighted version of equation 4 in the main manuscript as:

$$S^i(E_b) = w(m,n)\Big(\big(S(i,m) + S(m,i)\big) - \big(S(i,n) + S(n,i)\big)\Big). \tag{96}$$

Here $w(m,n)$ is the weight of the link connecting nodes m and n. The resulting quality function is identical in form. The quality function Q_{wgt} is mathematically represented as:

$$Q_{wgt} = \sum_{i,j} [(D - A)S + S(D - A)]\delta(i,j). \tag{97}$$

We resort to the same algorithms to determine overlap as in the unweighted case. For weighted networks, equation 3 in the main manuscript modifies to:

$$B(i,j) = d^i_{wgt}\Big(S(i,j) - \frac{1}{d^i_{wgt}} \sum_{k \in N(i)} w(i,k)S(k,j)\Big) + d^j_{wgt}\Big(S(i,j) - \frac{1}{d^j_{wgt}} \sum_{k \in N(j)} w(k,j)S(i,k)\Big), \tag{98}$$

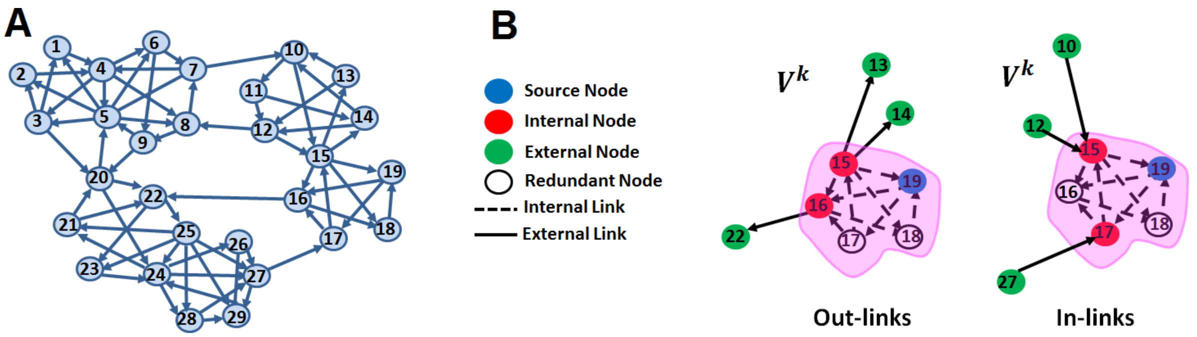

Figure 13: Laplacian modularity for a directed network. *A: A strongly connected directed network of 29 nodes B: Defining communities in the strong sense for a node in subgraph C (or V^k) with respect to out-links and in-links. The strong community criterion for the blue node ([19]) in subgraph, C, shaded pink (nodes [15,16,17,18,19]) over the 3 interfacing out-links with internal nodes ([15,16]) in red and external nodes ([13,14,22]) in green is:* $2S(19,15) + S(19,16) > S(19,13) + S(19,14) + S(19,22)$. *The strong community criterion for the blue node ([19]) in subgraph, C, shaded pink (nodes [15,16,17,18,19]) over the 3 interfacing in-links with internal nodes ([15,17]) in red and external nodes ([10,12,27]) in green is:* $2S(15,19) + S(17,19) > S(10,19) + S(12,19) + S(27,19)$.

where $d^i_{wgt} = \sum_j w(i,j)$. The weighted Laplacian will react continuously to the vanishing of an edge and the extension of Laplacian modularity to weighted networks cannot be arbitrarily manipulated. Note that the heat kernel for weighted networks (S in Laplacian modularity) is given by matrix exponential $H_t = e^{-t(I-D^{-1}A)}$ where A is the weighted adjacency matrix, $A(i,j) = w(i,j)$ and d^i_{wgt} are the elements of the diagonal matrix D.

4.2 Directed Networks

We naturally extend our Laplacian modularity framework to directed networks. Let G be a strongly connected directed graph with $|V|$ nodes and E be a set of directed edges. Strong connectivity ensures that the dynamics over time is ergodic useful for DLM. Edge directionality changes the calculation of boundary cohesion. Specifically, instead of the entire set of interfacing links E_B for subgraph C as in undirected communities, we consider two sets of interfacing links, in-links E_{in} (those that point towards the subgraph) and out-links E_{out} (those that point away from the subgraph). Formally, if node pair associated with a directed edge is $[m,n]$ where m is the origin node and n the destination node, then E_{in} is the set of edges with $m \notin C, n \in C$ and E_{out} is the set of edges with $m \in C, n \notin C$. The two sets of contributions for internal and external nodes used in the boundary cohesion calculation are modified to account for in-links and out-links separately (see Figure 13). Mathematically, $S^i(C_{in})$ and $S^i(C_{out})$ are represented as:

$$S^i(C_{in}) = \sum_{E_{out}} S(m,i) + \sum_{E_{in}} S(i,m) \tag{99a}$$

$$S^i(C_{out}) = \sum_{E_{out}} S(n,i) + \sum_{E_{in}} S(i,n). \tag{99b}$$

Following a similar derivation, the quality function to be optimized becomes:

$$Q_{dir} = \sum_{i,j} [(D_{out} - A_{dir})S + S(D_{in} - A_{dir})]\delta(i,j). \tag{100}$$

Here A_{dir} is the adjacency matrix with the $A_{dir}(i,j)^{th}$ entry denoting a directed edge from node i to j, D_{out} is a diagonal matrix of out-degrees, i.e., $D_{out} = diag(d_{out})$ where d^i_{out} is the ith element corresponding to node i of the out-degree vector given by $d^i_{out} = \sum_j A_{dir}(i,j)$, D_{in} is a diagonal matrix of in-degrees, i.e., $D_{in} = diag(d_{in})$ where d^i_{in} is the ith element corresponding to node i of the out-degree vector given by $d^i_{in} = \sum_j A_{dir}(j,i)$, S is the connectivity matrix, and $\delta(i,j)$ is 1 if i and j belong to the same community and 0 otherwise.

We also introduce the directed counterpart of the random walk Laplacian so as to be able to use eigen decomposition for efficient optimization of the DLM quality function. The probability transition matrix M_{dir} is given by $M_{dir} = D^{-1}_{out}A_{dir}$, indicating the probability of leaving a node is evenly split among its adjacent neighbors. Adjacent neighbors of a node i in a directed network are those connected with out-links starting from node i. Naive pre and post multiplication by an exponent of the degree matrix doesn't convert M into a symmetric normalized matrix, as in the undirected case. Instead, the normalized symmetric matrix N_{dir} for directed graph is given by $N_{dir} = \Pi^{1/2} M_{dir} \Pi^{-1/2}$ where Π is a diagonal matrix with elements corresponding to the stationary distribution vector π for the directed graph (*Chu97*). The stationary distribution vector is well defined for a strongly connected graph and is given by the eigenvector corresponding to eigenvalue 1 of M_{dir}. The random walk Laplacian Δ_{M_dir} and its normalized counterpart $\mathcal{L}_{dir}$ are given by $\Delta_{M_dir} = I - M_{dir}$ and $\mathcal{L}_{dir} = I - N_{dir}$ where I is an identity square matrix of size $|V|$. Let the spectral decomposition of the normalized Laplacian be again given by $\mathcal{L}_{dir} = \Phi\Lambda\Phi^T$ as in the undirected case. The eigen decomposition of the normalized transition matrix is then given by $N_{dir} = \Phi\Lambda'\Phi^T$ with same set of eigenvectors and eigenvalues related as $\Lambda' = I - \Lambda$ as in the undirected case. Using the above relations, the probability transition matrix M^t_{dir} for discrete directed random walks at time t is:

$$M^t(i,j) = \sum_{k \geq 1} \lambda'^t_k \Phi_k(i)\Phi_k(j)\sqrt{\frac{\pi_j}{pi_i}}. \tag{101}$$

The exponential matrix $e^{-t\Delta_{M_{dir}}}$, denoting the probability transition at time t for continuous time directed random walks is given by:

$$H_t(i,j) = \sum_{k \geq 1} e^{-t\lambda_k} \Phi_k(i)\Phi_k(j)\sqrt{\frac{\pi_j}{\pi_i}}. \tag{102}$$

In both the above equations, the $(i,j)^{th}$ term is the probability of starting at i and reaching j in time t. It is important to note that all formulaes for undirected networks reconcile with the

ones for directed networks in the Laplacian modularity as well as DLM frameworks. Thus, one efficacy of our formalism is that undirected networks are a special case of directed networks. However, the Laplacian and DLM matrices for directed networks are not symmetric in general.

Another appealing feature of our approach using random walks is that it provides a rigorous formalism of modularity for directed networks. Modularity Q_{dir}^{mod} for directed networks is defined as:

$$Q_{dir}^{mod} = \frac{1}{\mid E \mid} \sum_{i,j} [A - \frac{d_{in} d_{out}^T}{\mid E \mid}] \delta(i,j). \tag{103}$$

Here d_{in} and d_{out} are $|V| \times 1$ length vectors of the in-degree and out-degree respectively ($d_{in}^i = \sum_j A_{dir}(j,i)$) (*LN08*). However, this definition of modularity has critical limitations as expressed in (*KSJ10*), particularly the inability to coherently account for edge directionality but instead favor partitions with high edge densities. In order to circumvent this problem, the modification proposed in (*KSJ10*) is to instead define modularity as:

$$\tilde{Q}_{dir}^{mod} = \sum_{i,j} [\Pi M_{dir} - \pi \pi^T] \delta(i,j). \tag{104}$$

However, as $\Pi M_{dir} \neq A$ in general, the above definition introduces an undesirable transformation to the original adjacency matrix, i.e., we introduce structural changes to the topology of the original network. We overcome this limitation as well as limitation of original modularity for directed networks with our own version which appears naturally from the properties of random walks. It is given by:

$$\hat{Q}_{dir}^{mod} = \frac{1}{\mid E \mid} \sum_{i,j} [A - d_{out} \pi^T] \delta(i,j). \tag{105}$$

This definition stems from defining modularity matrix as $D_{out}[M_{dir} - M_{dir}^{\infty}]$ where M_{dir}^{∞} is the matrix of stationarity distribution. M_{dir}^{∞} is given by $\mathbb{1}\pi^T$ where $\mathbb{1}$ is a $|V| \times 1$ vector with all coordinates 1. As $D_{out} M_{dir} = A$ and $D_{out} M_{dir}^{\infty} = d_{out}\pi^T$, $D_{out}[M_{dir} - M_{dir}^{\infty}]$ simplifies to $[A - d_{out}\pi^T]$ as in equation 105. Note that the modularity matrix reduces to $D[M - M^{\infty}]$ for undirected networks. We use the relation $M^{\infty} = \mathbb{1}\pi^T$ where $\pi = \mathbb{1}D/vol$ and $DM = A$ to recover the original modularity matrix given by $[A - \frac{d^T d}{vol}]$. Overall, we contend that equation 105 offers a more coherent formalism of modularity for directed networks than those proposed previously.

4.3 Signed networks

In signed networks, there are both positive and negative links. We perform a separate analysis for positive and negative links:

$$\begin{aligned} S_+(V_+) &= S_+^T \mathcal{V}_+ + S_+ \mathcal{V}_+ \\ S_-(V_-) &= S_-^T \mathcal{V}_- + +S_- \mathcal{V}_-. \end{aligned} \tag{106}$$

Here $\mathcal{V}_-, \mathcal{V}_+$ are the membership vectors indicating boundary nodes for the positive and negative networks with connectivity potentials S_+, S_-. Following a similar analysis, the quality function for signed networks is:

$$Q_{sgn} = \sum_{i,j}[(D_+ - A_+)S_+ + S_+(D_+ - A_+) - (D_- - A_-)S_- - S_-(D_- - A_-)]\delta(i,j). \quad (107)$$

Here D_+, D_- are the positive and negative degrees, A_+, A_- are the positive and negative adjacency matrices. In terms of the Laplacian for the positive and negative adjacency network, the equation becomes:

$$Q_{sgn} = \sum_{i,j}[L_+S_+ + S_+L_+ - L_-S_- - S_-L_-]\delta(i,j). \quad (108)$$

Here A is the signed adjacency matrix and D is sum of signed diagonal degrees. Note that when the resulting matrix is asymmetrical, we symmetrize it, in order to apply the Louvain algorithm.

4.4 Multislice Networks

Another class of networks receiving a lot of attention are multislice networks that are defined by coupling multiple adjacency matrices. Each slice encodes connections that may vary over time or across different attributes of the network. (*MRM*$^+$*10*) generalizes modularity for multislice networks by making a connection between Laplacian dynamics and null models. The general nature of our framework enables extending our definition of communities to multislice networks with the additional feature of identifying overlap unlike (*MRM*$^+$*10*).

A natural feature of a multislice network is that it introduces replicates of each node in every slice. Hence we get a set of $|V| \times Y$ nodes, V_{multi} for which we wish to explore the community structure where Y is the number of slices. Let us represent nodes in unweighted undirected multislice networks as i_s where the subscript s indicates the slice under consideration for node i. The edges in the network $E_{multislice}$ are broadly characterized into two types, E_{multi}^{intra} indicating an edge between node pair $[i_s, j_s]$ in the same slice and E_{multi}^{inter} indicating an edge between the same node i in different slices r and s (node pair $[i_r, i_s]$).We construct the multislice adjacency matrix A_{multi} in order to quantify random walks on the network:

$$A_{multi}(i_s, j_s) = \begin{cases} 1 & \text{if} \quad (i_s, j_s) \in E_{multi}^{intra} \\ \omega & \text{if} \quad (i_r, i_s) \in E_{multi}^{inter} \\ 0 & \text{otherwise.} \end{cases} \quad (109)$$

Here the parameter ω controls the coupling between slices. The diagonal matrix is given by $D_{multi} = diag(d_{i_s})$ where $d_{i_s} = \sum_{j_r} w(i_s, j_r)$. We account for intra- and inter-slice interfacing links separately for internal and external nodes (see Figure 14). Let the set of intra-slice interfacing links for subgraph C be E_{intra} and the set of inter-slice interfacing links be E_{inter}.

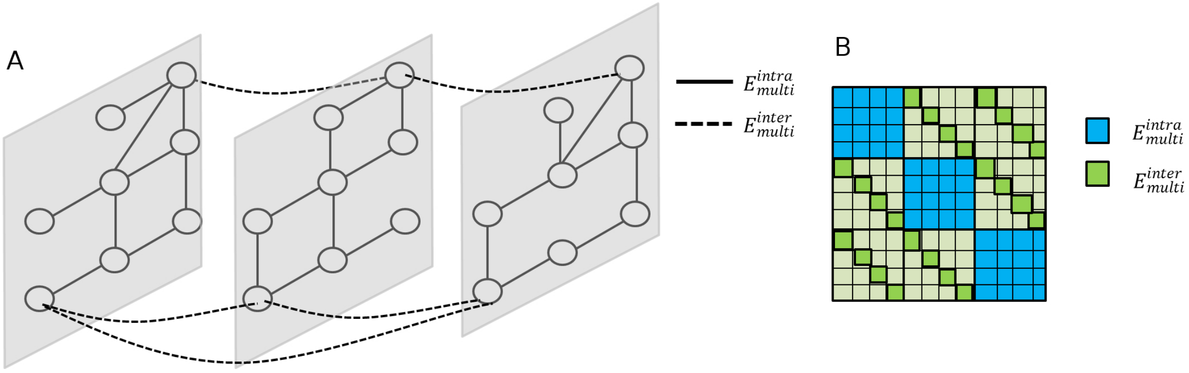

Figure 14: Laplacian modularity for a multislice network. *A: An illustrative multislice network with dashed inter-slice links and solid intra-slice links. B: Constructing the adjacency matrix* A_{multi} *for the multislice network. The blue areas correspond to intra-slice links and green blocks correspond to the placement of inter-slice links*

Mathematically, the list of the four contributions are:

$$S^{i_s}(C_{in \cap intra}) = \sum_{E_{intra}} (S(m_s, i_s) + S(i_s, m_s)) \tag{110a}$$

$$S^{i_s}(C_{in \cap inter}) = \sum_{E_{inter}} (S(i_r, i_s) + S(i_s, i_r)) \tag{110b}$$

$$S^{i_s}(C_{out \cap intra}) = \sum_{E_{intra}} (S(n_s, i_s) + S(i_s, n_s)) \tag{110c}$$

$$S^{i_s}(C_{out \cap inter}) = \sum_{E_{inter}} (S(i_r, i_s) + S(i_s, i_r)). \tag{110d}$$

The net internal boundary cohesion is then calculated as $(S^{i_s}(C_{in \cap intra}) + S^{i_s}(C_{in \cap inter}))$. Similarly, the net external boundary cohesion is calculated as $(S^{i_s}(C_{out \cap intra}) + S^{i_s}(C_{out \cap inter}))$. For notational simplicity, let us denote $(S^{i_s}(C_{in \cap intra}) + S^{i_s}(C_{in \cap inter}))$ as $S^{i_s}(C_{in})$, and represent $(S^{i_s}(C_{out \cap intra}) + S^{i_s}(C_{out \cap inter}))$ as $S^{i_s}(C_{out})$. Then the definitions of strong and weak communities are given by:

Community in the strong sense: The subgraph C is a community in a strong sense if:

$$S^{i_s}(C_{in}) > S^{i_s}(C_{out}), \forall i \in C. \tag{111}$$

In a strong community, the internal boundary cohesion of all (intra or inter-slice) nodes is greater than its external value for subgraph, C.

Community in the weak sense: Then the subgraph C is a community in a weak sense if:

$$\sum_{i_s \in C} S^{i_s}(C_{in}) > \sum_{i_s \in C} S^{i_s}(C_{out}). \tag{112}$$

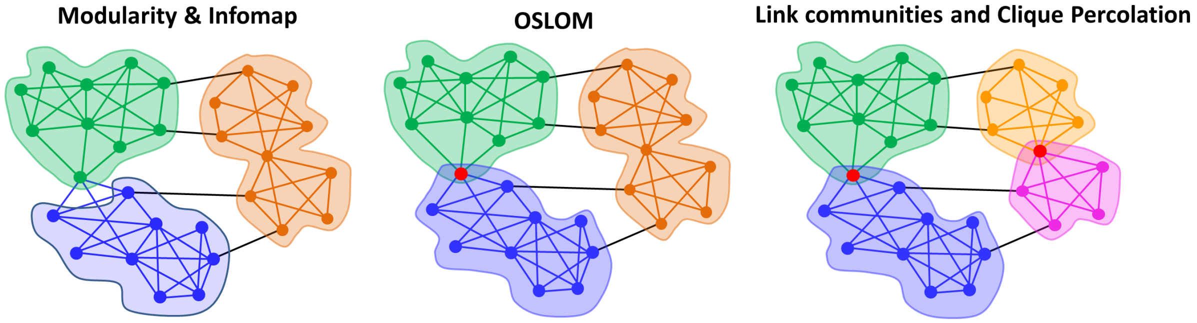

Figure 15: Results of other community detection methods on synthetic graph. *The identified communities for the same synthetic network of 29 nodes by different community detection methods; Left:Modularity and Infomap, Center: OSLOM, Right: Link communities and clique percolation. Observe no other method identifies the 6 communities as in Figure 3 in the main manuscript.*

In a weak community, the sum of internal boundary cohesion over all (intra or inter-slice) nodes in C is greater than the sum of external boundary cohesion, for subgraph, C.

Using these sets of definition, the quality function to be optimized takes the familiar form:

$$\tilde{Q}_{multi} = \sum_{i_s, j_r} [(D_{multi} - A_{multi})S + S(D_{multi} - A_{multi})]\delta(i_s, j_r). \tag{113}$$

The spectral representations for S can be derived by following the procedure in Section 3.1. The multislice quality function in equation 114 is can identify overlap in multislice networks using the same principles derived for single slice networks.

An alternate way is to neglect the cohesion from inter-slice coupling and consider it as a free parameter. In order words, only the intra-slice edges are considered while calculating the average similarity over the 1-neighborhood. In such a scenario, the quality function takes the form of the quality function introduced in (*MRM*$^+$*10*), and mathematically represented as:

$$\tilde{Q}_{multi} = \sum_{i_s, j_r} \left([(D^s_{multi} - A^s_{multi})S^s + S^s(D^s_{multi} - A^s_{multi})]\delta_{sr} + \delta_{ij}(C_{jsr} + C_{isr}) \right) \delta(i_s, j_r) \tag{114}$$

Here superscript s indicates the slice under consideration, and C_{jsr} is the parameter representing the inter-slice weights.

Overall, in this section we show that the Laplacian modularity framework is one of the most complete architectures to determine community structure in various network types.

5 Network datasets with known community assignment

In this section we first introduce other community detection methods used for comparison in the main manuscript and then describe the metric used to evaluate the quality of partitions, followed by brief description of each network along with the associated partition obtained by our method.

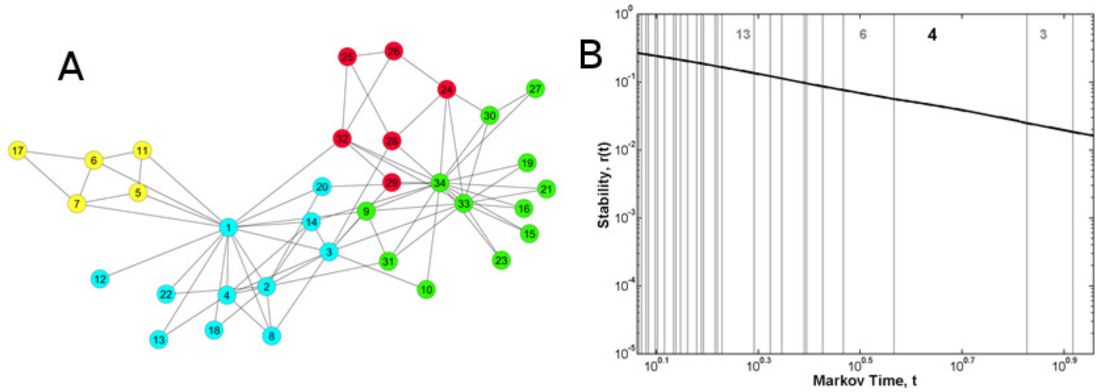

Figure 16: Diffusion based Laplacian modularity applied to karate network. *A: Identified communities in the karate network B: Stability curve for the karate network*

5.1 Other community detection methods

Modularity Modularity maximization is the most popular method for non-overlapping community detection (*New06b*). Modularity measures the excess of links relative to a baseline, usually taken to be the configuration model. The modularity quality function initially proposed to detect non-overlapping communities in unweighted and undirected networks, has been extended to weighted (*New04a*), directed (*LN08*), overlapping (*NMCM09*) and multi-slice networks (*MRM*$^+$*10*). Despite its wide appeal, modularity suffers from the resolution limit and numerous approaches have been proposed to overcome this limit (*AFG08, BHLP11, TVDN11*). Modularity optimization is provably NP-hard (*BDG*$^+$*08*), so we use the popular Louvain method to assess the performance of modularity maximization in a practical context (*BGLL08*).

Infomap The Infomap algorithm detects communities by minimizing the code length of map equation encoded using information theoretic principles (*RB08*). Recent experiments on benchmarks demonstrate the Infomap algorithm to be the one of the most accurate method for community finding (*For10*). Although the Infomap does not suffer from the resolution limit, it has the tendency to overpartition a network (the field-of-view limit) (*SDYB12*). We use the implementation provided by the authors to minimize the code length (out of 100 restarts) and derive the final community structure.

OSLOM: OSLOM, an acronym for ‘order statistics local optimization method’, is one of the most versatile methods of community detection capable of detecting overlapping and hierarchical communities in weighted, directed and dynamic networks (*LRRF*). OSLOM like modularity uses the configuration model to estimate statistical significance of communities. In order to overcome the resolution limit posed by this null model, it performs an iterative cluster search within the detected communities, and consequently, limits the horizon of the network under exploration.

Link Clustering Link clustering defines a community to be a group of links instead of tightly

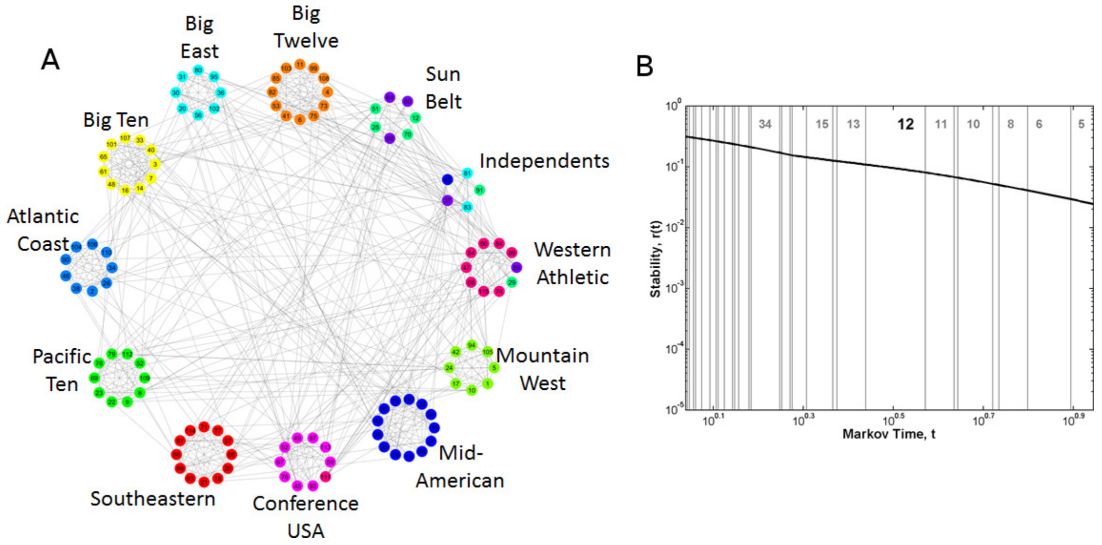

Figure 17: Diffusion based Laplacian modularity applied to football network. *A: Each circular layout of nodes corresponds to one ground truth community in the football network. The communities identified by our method are color coded B: Stability curve for the football network*

connected nodes and builds an agglomerative link-dendrogram to uncover overlap and hierarchy (*ABL10*). The link dendrogram is cut at the level which maximizes the partition density in order to obtain the most relevant community structure. Although with its own set of merits, the unconventional approach of grouping links is symmetric to the traditional approaches grouping nodes, and hence, not *a priori* better as argued in (*For10*). We contend that the Laplacian Modularity can be used to determine the overlapping nodes in the line graph, or equivalently the boundary links in the original graph and overcome the limitations of link clustering.

Clique Percolation The clique percolation method is one of the earliest approach to detect overlapping communities in networks (*PDFV05*). The method proceeds by first finding all complete subgraphs or cliques of size k and nodes that belong to multiple disjoint (or non-percolating) cliques are classified as overlapping nodes. The parameter k provides an intuitive way to define the strength of communities and acts as an effective resolution parameter. However, the rigid definition of clique communities makes it undesirable for extremely dense or sparse networks leading to giant components or absence of communities, respectively for all values of k (*ABL10*). We scan over the entire range of k and use the optimal k to demonstrate its performance.

The above community detection methods are used for comparison in the main manuscript (also see Figure 15), and we introduce and compare our method to spectral community detection methods in section 7.2.

5.2 Results on networks with known ground truth

Omega Index

The omega index ω is an extension of the adjusted Rand index to overlapping communities (*CD88*). It is defined as:

$$\omega(C_i, C_j) = \frac{\omega_u(C_i, C_j) - \omega_e(C_i, C_j)}{1 - \omega_e(C_i, C_j)}. \tag{115}$$

where C_i, C_j are the partitions to be compared, $\omega_u(C_i, C_j)$ is the fraction of node pairs occurring together in same number of communities in C_i and C_j, and $\omega_e(C_i, C_j)$ is the expected value of this fraction. Intuitively, $\omega_u(C_i, C_j)$ is the observed agreement between the partitions C_i and C_j, whereas $\omega_e(C_i, C_j)$ is the expected agreement of the partitions. The omega index is then observed agreement adjusted by expected agreement divided by the maximum possible agreement, i.e., 1 adjusted by expected agreement.

Karate club

The karate club network has served as a proving ground for many community detection algorithms. The network was constructed by Wayne Zachary and consists of ties between 34 members of a karate club at a US university in the 1970s (*Zac77*). The club split up into two groups following disputes among the members over the price of karate lessons. Our community detection algorithm splits the network into 4 communities, the same as modularity optimization (Figure 16). It is interesting to note that the 2-way partition obtained in the long time limit is identical to the original community assignment by Zachary. Hence, it may be useful to analyze partitions beyond t_{end} in certain cases, for example if the number of partitions is known apriori.

Football

The football network compiled by M. Girvan and M. Newman contains the set of American football games played by Division IA colleges during regular season in Fall 2000 (*GN02*). The ground truth community assignment is according to the conference each team belongs to. There are in total 12 conferences: Atlantic Coast, Big East,Big Ten, Big Twelve, Conference USA, Independents, Mid-American, Mountain West, Pacific Ten, Southeastern, Sun Belt and Western Athletic. Our community detection algorithm correctly partitions the network into 12 groups. In Figure 17 we see that nine of the twelve conferences are correctly identified without any misclassifications. The 3 other conferences with partial misclassifications also reveal interesting features about the network. For example, the group of Independents comprising of 5 universities is not a conference, but institutions not affiliated with NCAA. We see that institutions in Independents all are classified in another conference, presumably with the conference it has the closest affiliation with. Louisiana Tech University shifted from the Sun Belt conference to Western Athletic conference in 2001. Consequently, node 59 corresponding to Louisiana Tech University in the western athletic conference is misclassified to be in the sun belt conference by our method.

Polbooks

The political books network compiled by Valdis Krebs represents co-purchased political books sold by Amazon.com around the time of the 2004 presidential election. The ground truth com-

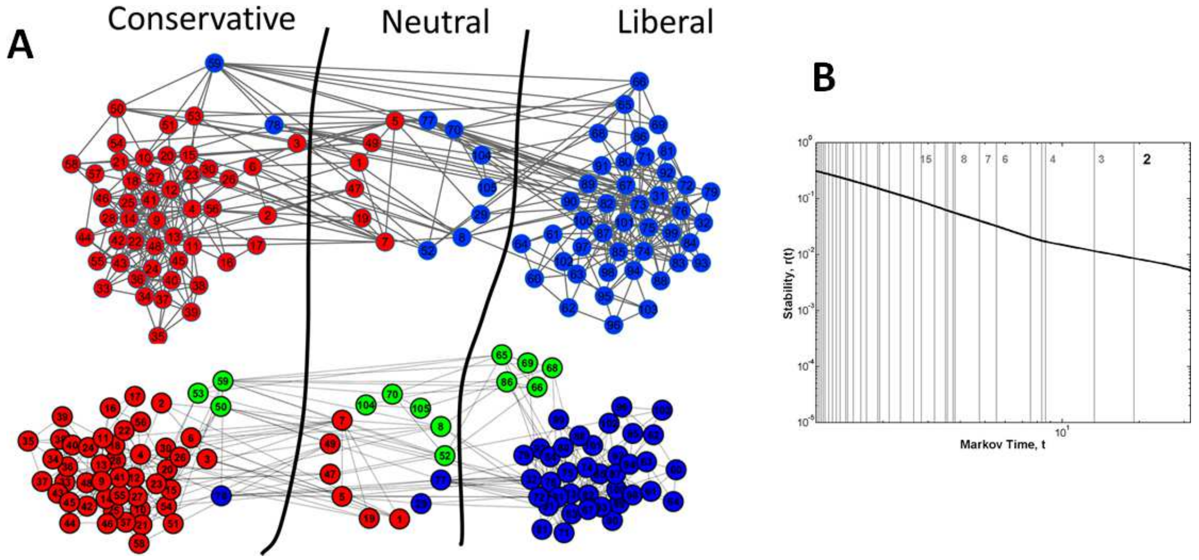

Figure 18: Diffusion based Laplacian modularity applied to political books network. *A:The three communities in the ground truth are separated by the two black lines. They correspond to conservative, neutral and liberal communities from left to right. The plot in top shows the two communities identified by our method. The nodes are colored red and blue. The plot in the bottom shows the 3-way partition identified by the stability curve. The nodes are colored red, green and blue. B: Stability curve for the polbooks network*

munity assignment corresponds to the political philosophy advocated by the book as either conservative, neutral or liberal. In Figure 18 we see that our methods identifies two groups and classifies books in the ground truth neutral community to be either right-leaning or left-leaning based on the network topology. It is also interesting to note that the node 59 (Rise of the Vulcans) and node 78 (Bush at War) are written by journalists associated with the left-leaning Washington Post. The two nodes are misclassified to be liberal by our method, although the ground truth assignment is conservative. We also show the 3-way partition identified by the stability curve. The identified communities correspond well to conservative and liberal political books. We further investigate the assignment of neutral political books by our method. The liberal books classified as neutral (nodes 77 and 29) as well as the neutral political books classified as liberal (nodes 65,66,68,69,86) are all identified as overlapping nodes with membership to liberal and neutral communities at $\epsilon = 0$. This validates the relevance of the 3-way overlapping community structure identified by our method.

Polblogs

The political blogs network compiled by Lada Adamic and Natalie Glance represents the political blogosphere around the time of the 2004 presidential election (*AG05*). Nodes are classified to be liberal or conservative. Our method achieves the highest omega index among all community detection methods. The stability curve in shown in Figure 22.

Dolphin

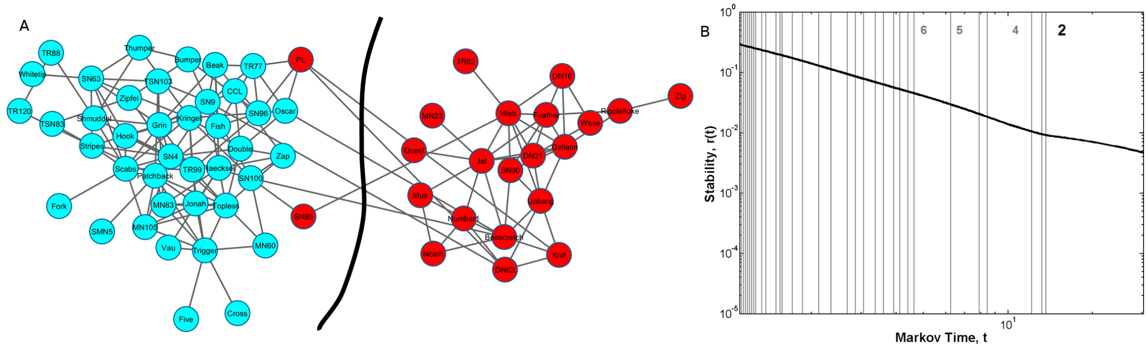

Figure 19: Diffusion based Laplacian modularity applied to dolphin network. *A: The two communities in the ground truth are separated by the black line. The two communities identified by our method are colored red and blue B: Stability curve for the dolphins network*

The dolphin social network compiled by D. Lusseau represents frequent associations between 62 dolphins in a community living off Doubtful Sound, New Zealand (*LSB*$^{+}$*03*). The ground truth community assignment into 2 groups is primarily based on the gender of the dolphins. Our community detection method correctly identifies 2 clusters, correctly classifying all but 2 dolphins. The misclassified dolphins PL and SN89, structurally share more number of links with the identified group than with the other group, and hence, the identified communities correspond to a sound partition (19).

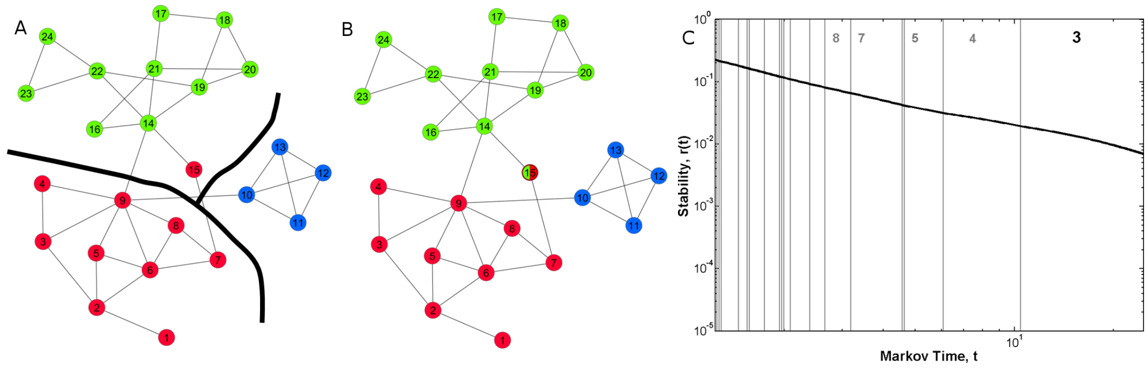

Figure 20: Diffusion based Laplacian modularity applied to strike network. *A: The black lines separate the network into its constituent ground truth communities. The communities identified by our method are color coded. B: The overlapping communities identified by our method at* $\epsilon = 0.5$ *B: Stability curve for the strike network*

Strike The strike network represents the communication structure among employees in a wood processing facility during a strike (*LM97*). The network displays fairly stringent demarcations between groups defined on age and language. The 3 ground truth communities correspond to English-speaking young employees, English-speaking old employees and finally Spanish-speaking young employees. Our community detection method correctly identifies the commu-

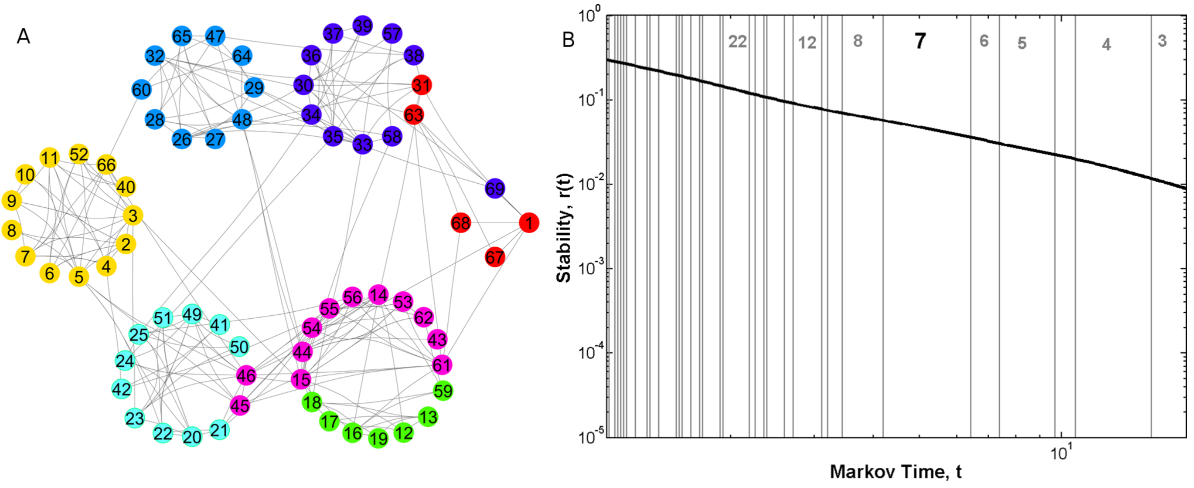

Figure 21: Diffusion based Laplacian modularity applied to highschool network. *A: Each circular layout of nodes corresponds to one ground truth community in the highschool network. The communities identified by our method are color coded B: Stability curve for the highschool network*

nities and misclassifies one node (Figure 20). Node 15 (Ozzie) is the father of node 14 (Karl), which explains the misclassification. Interestingly, node 15 is identified as an overlapping node, indicating it shares membership with the group of old English-speaking employees and the father of a young English-speaking employee (node 14).

Highschool

The highschool network, compiled by the National Longitudinal Study of Adolescent Health, represents self-reporting of friendship between students from grades 7 to 12 (*hig*). The ground truth community assignment corresponds to the students' grade, with the expectation that students belonging to the same grade form stronger associations than those belonging to different grades. In Figure 21 we see that our community detection method identifies 7 communities contrary to the 6 ground truth communities. Most nodes agree with the ground truth assignment in 5 of the 7 identified communities. However, the sixth ground truth community is split into 2 groups (colored pink and green) in our identified 7-way partition. Upon further investigating this community, it is revealed that it possesses hierarchical organization of friendship and the pink and green nodes correspond well with the group of white and black students, respectively. Hence, our community detection method is revealing of the community structure and the hierarchy present in the network.

Contact-1 and Contact-2

The two networks represent the face-to-face proximity between students and teachers in a primary school for 2 days (*SVB$^+$11*). Although, the original network is weighted, we analyze the unweighted version of the network. As in the highschool network, the ground truth communities correspond to the class and grade of the students along with a separate community for

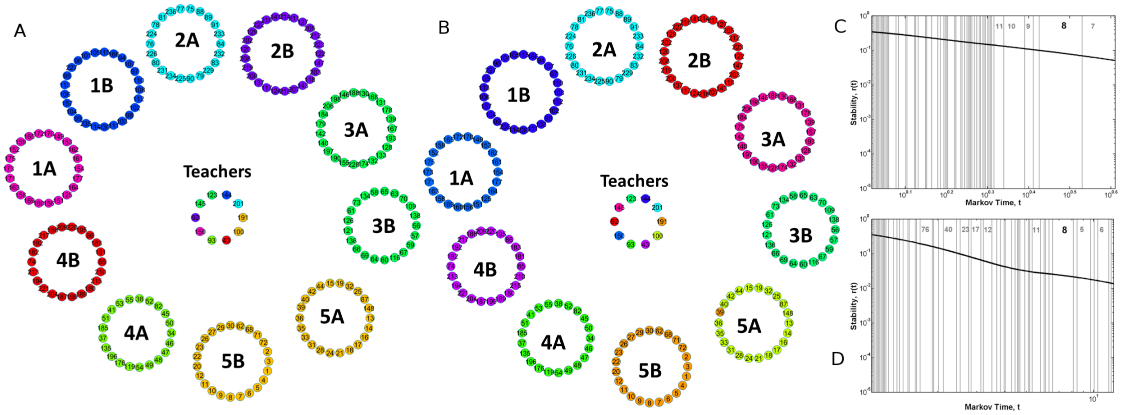

Figure 22: Diffusion based Laplacian modularity applied to contact-1 network. *A: Each circular layout of nodes corresponds to one ground truth community in the contact1 network. The communities identified by our method are color coded B: The communities identified by our method corresponding to the stable 10-way partition C: Stability curve for the contact1 network D: Stability curve for the polblogs network*

teachers. There are a total of 11 communities in the network, 10 communities of students and 1 of teachers. Figures 22 and 23 display the 8 and 9 communities identified by our automated method for contact-1 and contact-2 networks respectively. Observe that the 3A, 3B and 5A,5B communities are merged in contact-1 network suggesting hierarchical organization of the students at the same grade level but different divisions. The 10-way partition emerges as one of the stable partitions in the stability curve and the identified communities are color coded in Figure 22 B. All but 1 of the 226 students are correctly classified and the community of teachers is split by the class it teaches. Similarly, the 3A, 3B communities are merged in contact-2 network indicating hierarchy. Again, the 10-way partition emerges as one of the stable partitions in the stability curve and the identified communities are color coded in figure 23 B. All of the 228 students are correctly classified and the 10 teachers are assigned to the class it teaches.

Citation

The citation network comprises of computer science research papers categorized in 7 groups: neural networks, rule learning, reinforcement learning, theory, probabilistic methods, case based and genetic algorithms. The stability curve for this dataset is displayed in Figure 23 D.

We compare our method against Neo K-means (*WDG15*) to analyze the quality of overlapping communities in real world networks with known ground truth. We showed that our method was superior to all other methods when we set our method to identify hard partitions, i.e., the threshold parameter to be 0 as the ground truth are hard paritions. When we set the threshold parameter to be 0.25, many communities identified for threshold value 0 remain unperturbed. However, the community quality in terms of omega index for some networks naturally deteriorate. Figure 24 shows that our method identifies better overlapping communities than Neo K-means on most networks, even though the number of ground truth communities are supplied

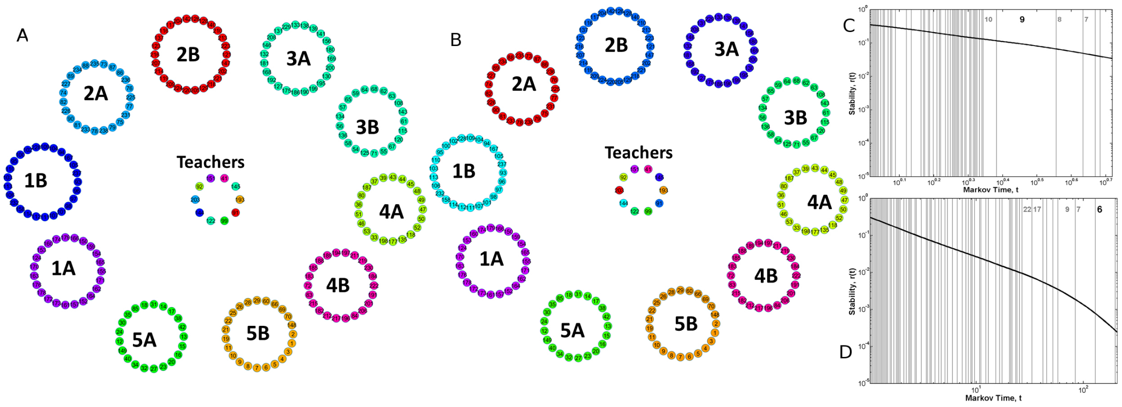

Figure 23: Diffusion based Laplacian modularity applied to contact-2 network. *A: Each circular layout of nodes corresponds to one ground truth community in the contact2 network. The communities identified by our method are color coded B: The communities identified by our method corresponding to the stable 10-way partition C: Stability curve for the contact2 network D: Stability curve for the citation network*

as an input to the Neo K-means algorithm.

6 Network datasets with unknown community assignment

In this section we first describe the four measures used to evaluate the quality of communities output by different community detection algorithms. Next, we briefly describe each of the 10 networks and the associated metadata used to evaluate the quality measures.

6.1 Measures

We follow the approach in (*ABL10*) to derive a composite performance metric for the communities identified in the network. The composite performance metric consists of 4 measures: 2 quality measures that assess the relevance of the discovered memberships and 2 coverage measures that quantify the quantity of information extracted. These measures are mathematically formalized as follows:

Community Quality: Community quality is evaluated using the network metadata under the assumption that nodes belonging to the same community are similar and hence, share more metadata than nodes belonging to different communities. This is quantified as:

$$Enrichment = \frac{\langle \mu(i,j) \rangle_{\delta(i,j)}}{\langle \mu(i,j) \rangle_{\varrho(i,j)}}. \tag{116}$$

where $\mu(i,j)$ is metadata-based similarity between nodes i and j, $\langle \mu(i,j) \rangle$ is the mean similarity and the subscripts $\delta(i,j)$, $\varrho(i,j)$ indicate the average calculated over all node pairs be-

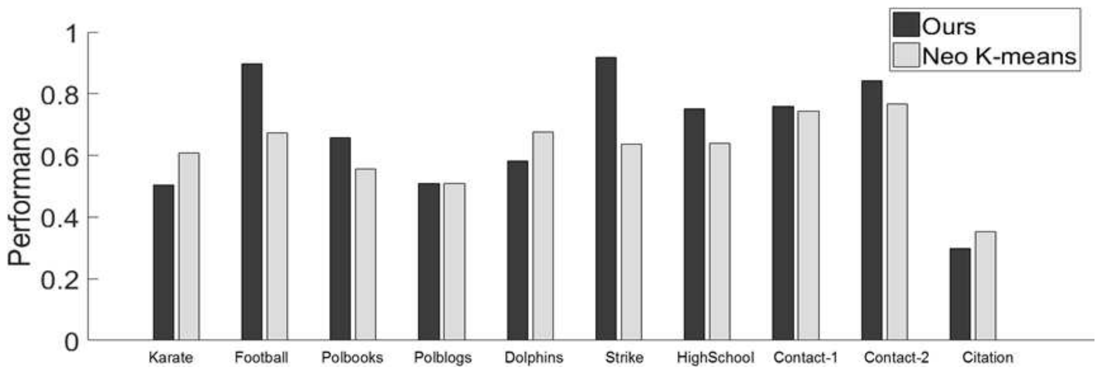

Figure 24: Comparison with Neo K-means on real-world networks.*Performance on ten networks with known community assignment measured with the omega index of identified communities against ground truth for our method, and Neo K-means with known K.*

longing to the same community or different community respectively. Unlike (*ABL10*) where the enrichment is calculated over the baseline similarity between all node pairs, we calculate the ratio of the average intra-community and inter-community similarity, which we discern to more discriminative. High intra-community similarity and low inter-community similarity lead to favourable values of enrichment.

Overlap Quality: To assess the quality of overlap output by a method, we calculate the mutual information between the number of memberships identified by the method and the overlap metadata of the network. Unlike (*ABL10*), homeless nodes are assigned to an independent community, and hence, have one membership (to itself). This avoids artificially increasing the mutual information due to homeless nodes which do not convey any overlap information, and consequently, methods identifying non-overlapping communities always have 0 overlap quality. Note that in the absence of any overlap metadata for a node, we take the number of associated memberships to be trivially 1. In 2 of the 10 networks, we use the average split-betweenness of overlapping nodes as a proxy for community quality instead of mutual information.

Community Coverage: Community coverage is the fraction of nodes which belong to non-trivial communities, i.e., of size 3 or higher. Community coverage hints at the quantity of useful membership information output by the community detection method.

Overlap Coverage: Overlap coverage measures the average membership of nodes belonging to non-trivial communities. Note that the community coverage and overlap coverage are identical for non-overlapping community methods.

The composite performance metric is derived by first normalizing all four measures by the corresponding best performing method, and next summing the four measures. A method which outputs the best community in terms of all 4 measures has composite performance value 4. The 4 measures are visually represented as a stacked bar chart, as in (*ABL10*).

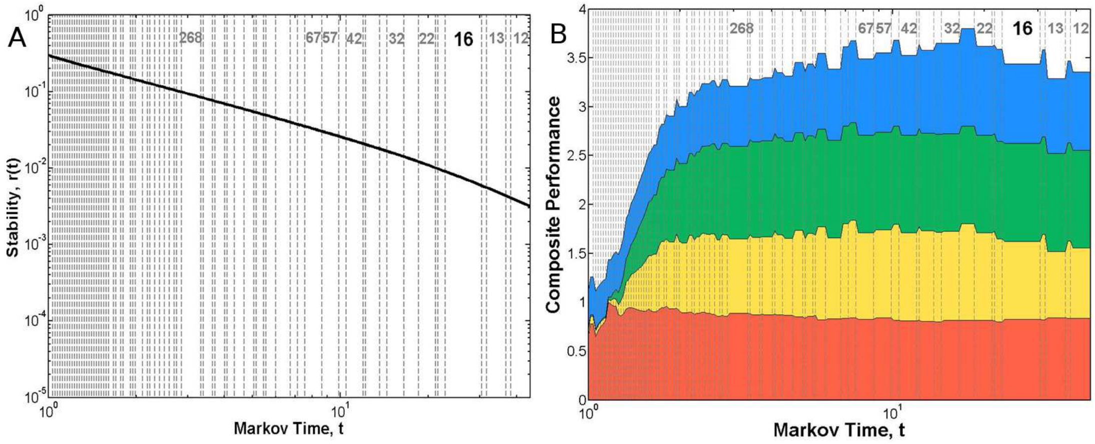

Figure 25: Diffusion based Laplacian modularity applied to S.cerevisiae network. *A: Stability curve for Saccharomyces cerevisiae network B: Variation of composite performance as a function of the stable communities identified by the stability curve over time shown as an area chart. The red corresponds to community quality, yellow to overlap quality, green to community coverage and blue to overlap coverage. The number of communities in the partitions with high persistence is shown above the composite performance index curve. The number of communities in the partition used for evaluation in the main manuscript is highlighted in bold.*

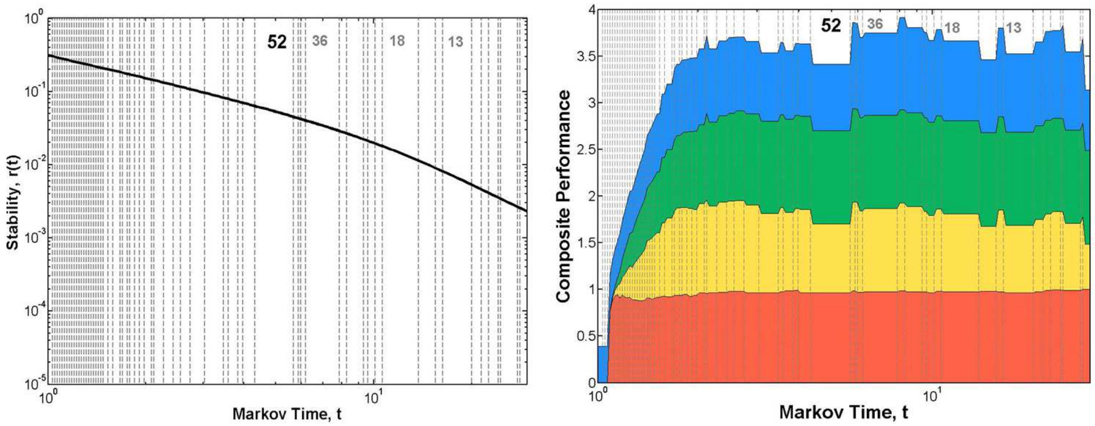

Figure 26: Diffusion based Laplacian modularity applied to H.sapiens network. *A: Stability curve for Homo sapiens network B: Variation of composite performance as a function of the stable communities identified by the stability curve over time shown as an area chart. The red corresponds to community quality, yellow to overlap quality, green to community coverage and blue to overlap coverage. The number of communities in the partitions with high persistence is shown above the composite performance index curve. The number of communities in the partition used for evaluation in the main manuscript is highlighted in bold.*

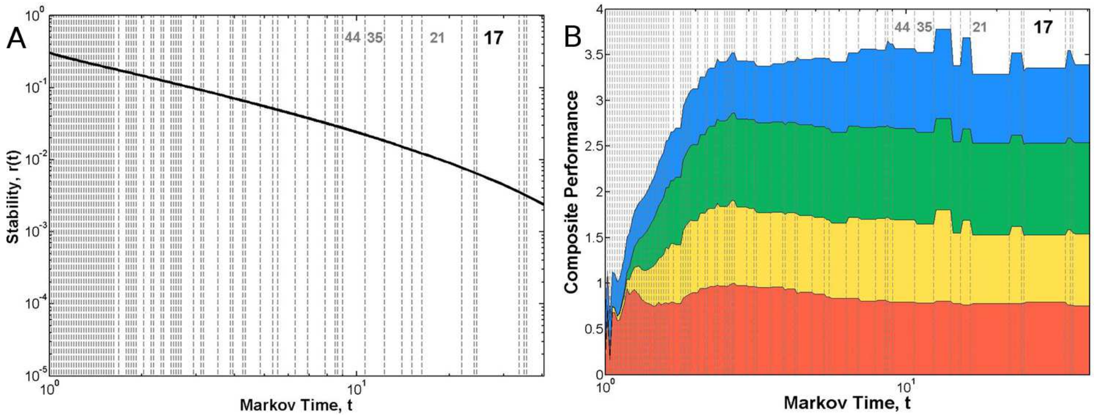

Figure 27: Diffusion based Laplacian modularity applied to C.elegans network. *A: Stability curve for Caenorhabditis elegans network B: Variation of composite performance as a function of the stable communities identified by the stability curve over time shown as an area chart. The red corresponds to community quality, yellow to overlap quality, green to community coverage and blue to overlap coverage. The number of communities in the partitions with high persistence is shown above the composite performance index curve. The number of communities in the partition used for evaluation in the main manuscript is highlighted in bold.*

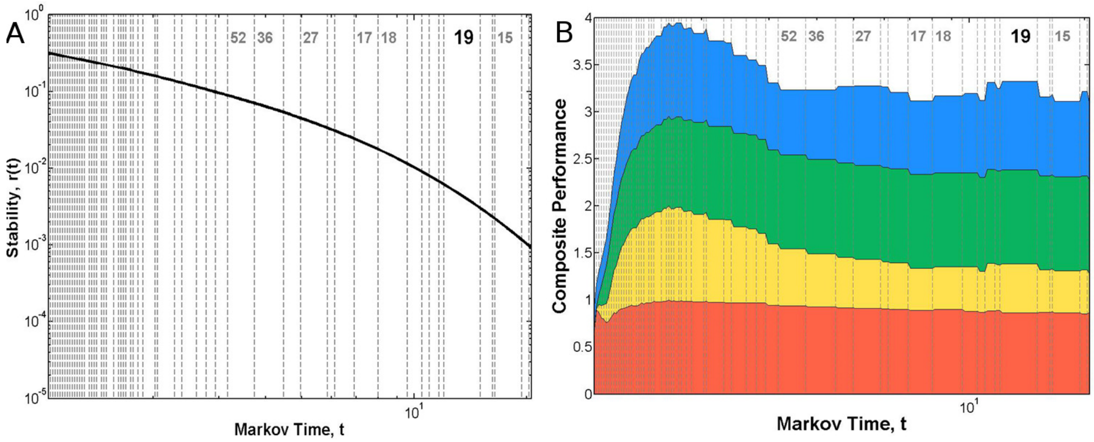

Figure 28: Diffusion based Laplacian modularity applied to D.melanogaster network. *A: Stability curve for Drosophila melanogaster network B: Variation of composite performance as a function of the stable communities identified by the stability curve over time shown as an area chart. The red corresponds to community quality, yellow to overlap quality, green to community coverage and blue to overlap coverage. The number of communities in the partitions with high persistence is shown above the composite performance index curve. The number of communities in the partition used for evaluation in the main manuscript is highlighted in bold.*

6.2 Biological networks

We analyzed the protein-protein interaction (PPI) network of Saccharomyces cerevisiae, Homo sapiens, Caenorhabditis elegans and Drosophila melanogaster. We use the Gene Ontology (GO)

terms as metadata to assess the quality of communities. The quality measures are first evaluated using GO annotations for biological process, molecular function and cellular components. The overall quality is simply taken as the average of these three quality measures.
Construction The PPI network of Saccharomyces cerevisiae is constructed using the yeast two-hybrid (Y2H) dataset published in (*YBY$^+$08*). The PPI network of Homo sapiens is constructed using the high quality Y2H interactions determined in (*YTT$^+$11*). The PPI network of Caenorhabditis elegans is constructed from the dataset released in (*KL13*). Finally, the PPI network of Drosophila melanogaster is taken from (*SLM$^+$04*). We use the largest component of each network.
Community quality Three separate directed acyclic graphs are constructed using the controlled vocabulary for biological process, molecular function and cellular components. The functional similarity between all pairs of proteins is then calculated from the directed acyclic graph using Lin's information theoretic definition (*cit98*). We use the implementation provided with (*YNP12*) . The corresponding enrichment values are calculated using equation 116 and the overall quality is taken as the average of these enrichment values.
Overlap quality Overlap quality is calculated for biological process, molecular function and cellular components to be the mutual information between the number of associated GO terms and number of memberships. The net overlap quality is taken to be the average of the three individual overlap qualities.

Figures 25, 26, 27 and 28 show the stability curve and the variation of composite performance as a function of the stable communities identified by the stability curve for the Saccharomyces cerevisiae, Homo sapiens, Caenorhabditis elegans and Drosophila melanogaster networks respectively. For all biological networks, we observe that communities with higher composite performance index than the one used for evaluation are identified.

6.3 Social networks

6.3.1 Facebook network

We analyzed the Facebook network of students in an American university graduating in the same year. Communities were evaluated using biographic information such as college major/minor, highschool they attended and dorms they reside in.
Construction The full Facebook network of Michigan university published in (*TKMP11*) consists of friendship links between students and faculty. Only the students stating biographic information in their profile are considered. We would expect students graduating in the same year to form communities based on major/minor or residency. Hence, the friendship circles of the students graduating in the year 2008 are used in constructing the network. We use the largest component of this network.
Community quality The metadata contains anonymized information regarding the students' major/minor, the dorms they reside in and their highschool. We build a bipartite network linking each student to its corresponding major/minor, dorm and highschool. The similarity between

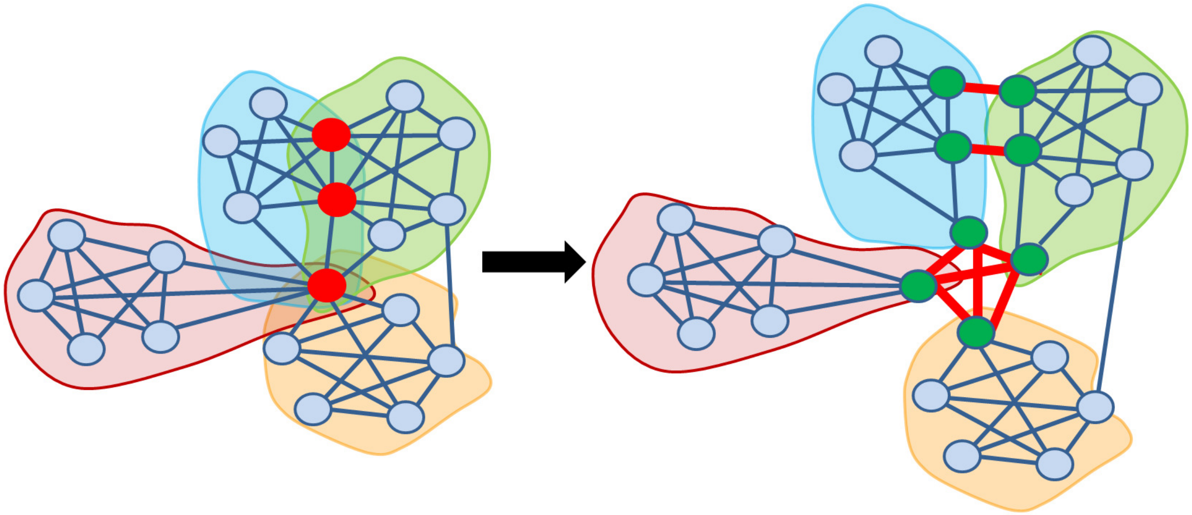

Figure 29: Split-betweenness evaluation. *Left network shows the communities identified by an algorithm with overlapping nodes colored red. Right network displays the transformed network used for overlap quality determination. The overlapping nodes are replicated such that the replicated nodes belong to only one community, shown in green. The overlap quality is the average edge betweenness of the edges colored red*

students is inferred from the one-mode projection onto the students of this two-mode bipartite network. The maximum similarity between two students is 3 if they share the same major/minor, dorm and highschool. The one-mode projected matrix is used to calculate the enrichment as per equation 116.

Overlap quality The community quality partially reflects the overlap quality as a student participates in multiple (possibly overlapping) communities corresponding to their major/minor, dorm and highschool. However, additional factors like a student's participation in hobby-clubs, college events or sports teams may contribute to forming friendship, and hence, communities that overlap. We use the concept of split betweenness to evaluate the quality of overlapping nodes due to extraneous information not available in the metadata. Unlike (*Gre07*) where the split betweenness metric is used to determine overlap, we use it to evaluate the quality of overlapping nodes. The network with overlapping nodes as determined by the community finding algorithm is first transformed into a similar network where all nodes and their linkages remain the same, but the nodes classified as overlapping. An overlapping node with membership to o communities is replicated o times in the transformed network such that each replicated node is a member to only one community, i.e., we convert the network with overlapping communities into one with no overlap by replicating overlapping nodes. The overlap quality is calculated to be the average edge betweenness of new edges introduced in the transformed network with higher values favored, i.e., a high value of average split betweenness hints at better overlap quality (see Figure 29).

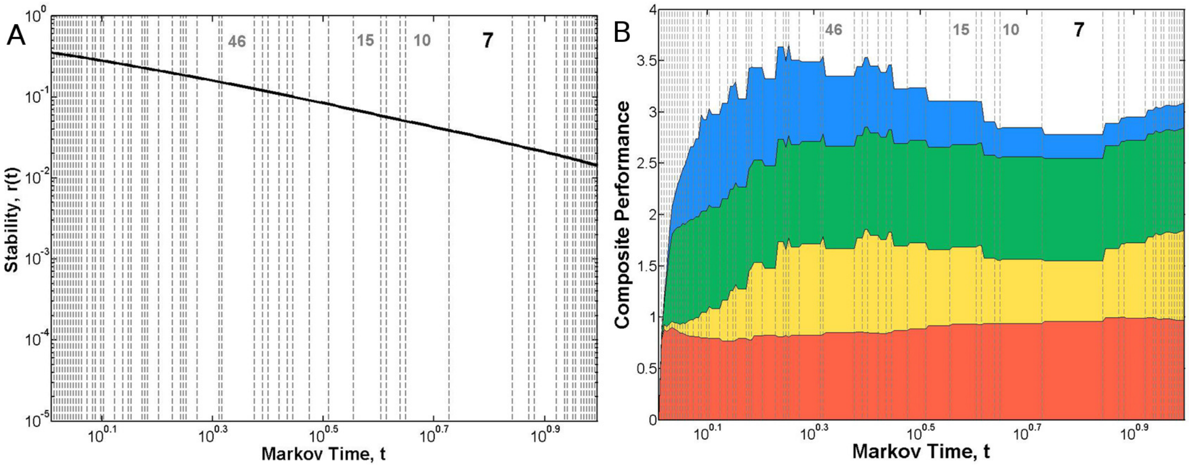

Figure 30: Diffusion based Laplacian modularity applied to Facebook network. *A: Stability curve for the Facebook network B: Variation of composite performance as a function of the stable communities identified by the stability curve over time shown as an area chart. The red corresponds to community quality, yellow to overlap quality, green to community coverage and blue to overlap coverage. The number of communities in the partitions with high persistence is shown above the composite performance index curve. The number of communities in the partition used for evaluation in the main manuscript is highlighted in bold. Communities with higher composite performance index than the one used for evaluation are identified.*

Figure 30 shows the stability curve and the variation of composite performance as a function of the stable communities identified by the stability curve for the Facebook network.

6.3.2 Cosponsorship network

The cosponsorship network consists of legislative collaborations between the United States House of Representatives for the 93rd to 108th Congresses (*Fol06*).

Construction As in (*ABL10*), we construct a bipartite network of house members and the bills they (co)-sponsored. Projecting this network onto the representatives results in a very dense, nearly complete graph for which methods like clique percolation with exponential running time fail to provide any meaningful communities. To avoid this, we only consider bills that contain 10 or less (co)-sponsors in building the bipartite network, and subsequently project it onto the representatives. In order to capture the tightest working relationships we delete links with weight less that 15, i.e., the final network has an edge between two house members if they (co)-sponsor 15 or more bills that contain 10 or less (co)-sponsors total. The giant connected component of this network consists of 560 nodes.

Community quality We follow the approach in (*SLM$^+$04*) to evaluate community quality using the common space score of house representatives. The common space score consists of two values between -1 and 1, wherein the first dimension represents liberal/conservative bias and

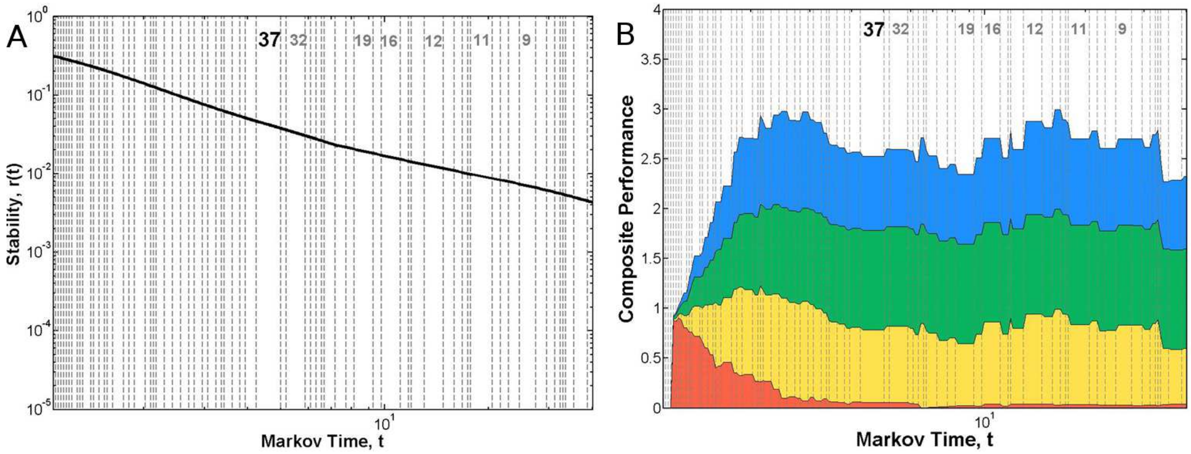

Figure 31: Diffusion based Laplacian modularity applied to cosponsorship network. *A: Stability curve for the cosponsorship network B: Variation of composite performance as a function of the stable communities identified by the stability curve over time shown as an area chart. The red corresponds to community quality, yellow to overlap quality, green to community coverage and blue to overlap coverage. The number of communities in the partitions with high persistence is shown above the composite performance index curve. The number of communities in the partition used for evaluation in the main manuscript is highlighted in bold. Communities with higher composite performance index than the one used for evaluation are identified.*

the second dimension is related to civil-rights and social issues. The similarity $\mu(i, j)$ between two house members is taken to be the Euclidean distance between the common space scores with smaller values denoting higher similarity. Hence, the overall enrichment is taken to be:

$$Enrichment = 1 - \frac{\langle \mu(i,j) \rangle_{\delta(i,j)}}{\langle \mu(i,j) \rangle_{\varrho(i,j)}}. \qquad (117)$$

Overlap quality The overlap quality is determined to be the mutual information between number of community memberships and the number of elected terms of representatives with the expectation that, representatives serving longer terms have (co)-sponsored more bills, and hence, participate in many collaborations.

Figure 31 shows the stability curve and the variation of composite performance as a function of the stable communities identified by the stability curve for the cosponsorship network.

6.4 Other networks

6.4.1 Word association

The word association dataset is compiled by University of South Florida and University of Kansas (*NMS98*). It consists of three-quarters of a million free association responses to 5019 stimulus words from more than 6000 participants.

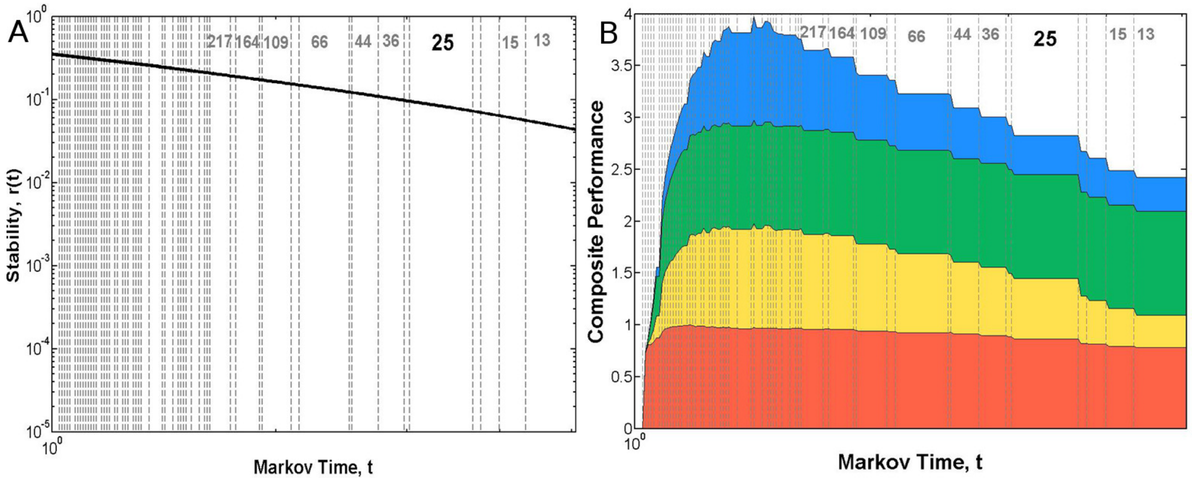

Figure 32: Diffusion based Laplacian modularity applied to word association network. *A: Stability curve for the word association network B: Variation of composite performance as a function of the stable communities identified by the stability curve over time shown as an area chart. The red corresponds to community quality, yellow to overlap quality, green to community coverage and blue to overlap coverage. The number of communities in the partitions with high persistence is shown above the composite performance index curve. The number of communities in the partition used for evaluation in the main manuscript is highlighted in bold. Communities with higher composite performance index than the one used for evaluation are identified.*

Construction Although there are 5019 stimulus words, the set of total words including responses has cardinality 10617. The edges represent discrete association of two words, i.e., the first meaningfully related word that came to a participant's mind in response to the presented word. We ignore the directionality and weights of the edges in the network, and hence, the final network for evaluation is undirected and unweighted.

Community quality We use the WordNet database to extract all synsets (or senses) associated with a word in the network. The Leacock-Chodorow similarity score (*LCM98*) between two words is calculated for all combination of associated synsets, based on the shortest path distance that connects the synsets and the maximum depth of the taxonomy in which the senses occur. The similarity between two words is then taken to be equal to the maximum Leacock-Chodorow score from these combinations and further, equation 116 is used to calculate the overall enrichment.

Overlap quality The more synsets a word is associated with, greater the likelihood of it participating in multiple communities. Hence, the overlap quality is determined to be the mutual information between the number of synsets and the number of community memberships for all words in the network.

Figure 32 shows the stability curve and the variation of composite performance as a function of the stable communities identified by the stability curve for the word association network.

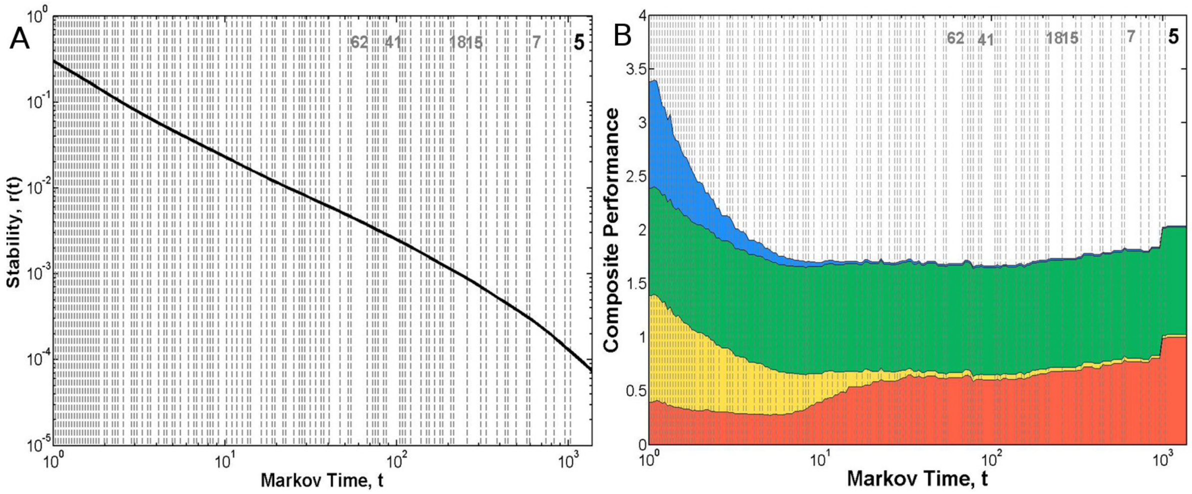

Figure 33: Diffusion based Laplacian modularity applied to citation network. *A: Stability curve for the citation network B: Variation of composite performance as a function of the stable communities identified by the stability curve over time shown as an area chart. The red corresponds to community quality, yellow to overlap quality, green to community coverage and blue to overlap coverage. The number of communities in the partitions with high persistence is shown above the composite performance index curve. The number of communities in the partition used for evaluation in the main manuscript is highlighted in bold. Communities with higher composite performance index than the one used for evaluation are identified.*

6.4.2 Citation network- PRL

The network is created using the citation information of papers published in the Physical Review Letters (PRL).

Construction The network is created using a subset of all papers published in the PRL. We consider all papers published in Volume-96 to Volume-100 to be nodes and place an undirected edge between two nodes, if any one cites the other. This network is disconnected and we take the largest connected component for our evaluation.

Community quality Each paper is associated with a general category such as, 'Atomic, Molecular, and Optical Physics', 'Gravitation and Astrophysics', etc. We define two papers to be similar if they share a paper category, i.e., $\mu(i, j) = 1$ if i and j share the same category, 0 otherwise. Equation 116 is used to calculate the community quality.

Overlap quality We assume that a paper with many authors is more likely to have diverse content, and hence, cited in multiple disciplines as compared to a paper with a single author. We calculate the overlap quality to be the mutual information between the number of non-trivial community memberships and number of authors of each paper in the network.

Figure 33 shows the stability curve and the variation of composite performance as a function of the stable communities identified by the stability curve for the citation network.

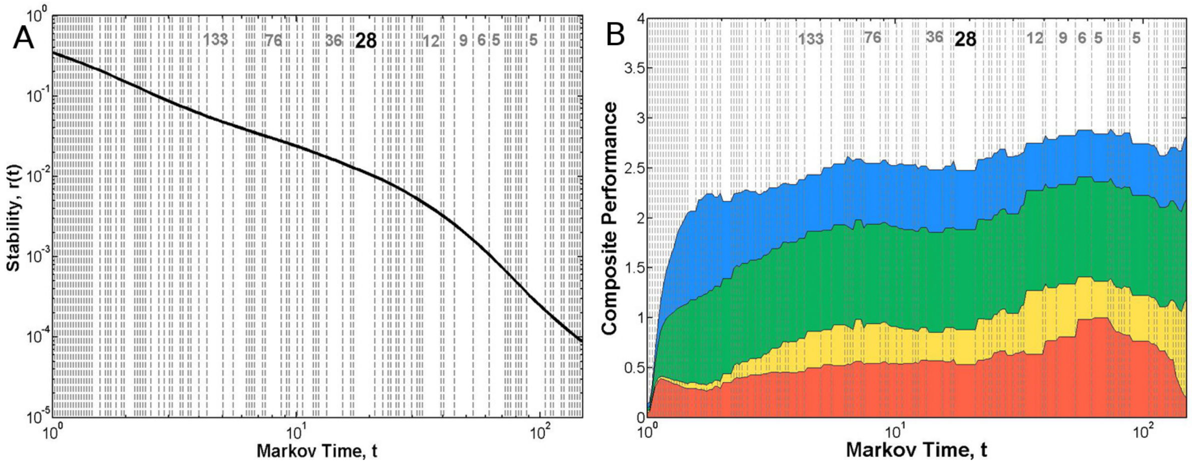

Figure 34: Diffusion based Laplacian modularity applied to air transport network. *A: Stability curve for the air transportation network B: Variation of composite performance as a function of the stable communities identified by the stability curve over time shown as an area chart. The red corresponds to community quality, yellow to overlap quality, green to community coverage and blue to overlap coverage. The number of communities in the partitions with high persistence is shown above the composite performance index curve. The number of communities in the partition used for evaluation in the main manuscript is highlighted in bold. Communities with higher composite performance index than the one used for evaluation are identified.*

6.4.3 Air Transport

The air transport network represents flights connecting airports all over the world.
Construction The network was created using the flights connectivity information between different airports available at www.openflights.org. The nodes represent airports and an edge between two nodes represents a flight connecting these two airports. We take the largest connected component for network evaluation consisting of 3412 airports.
Community quality The international organization for standardization(ISO) has codes for different regions in the world. We associate each airport to three features, its ISO-region, the country it belongs to and the continent it resides in. The similarity between two airports is taken to equal to the number of features it shares, i.e., has maximum similarity value 3 if it shares the region, country and continent; 2 if it shares country and continent; 1 if the airports are in the same continent; and 0 otherwise. Equation 116 is used to calculate the enrichment using the similarity values.
Overlap quality An airport is expected to overlap with different communities if there are many connecting flights emerging from it. This in turn depends on the betweenness of the airport, i.e., how many shortest paths (connecting flights) pass through that node (airport). Hence, we use the average split betweenness of overlapping airports as a measure of overlap quality.

Figure 34 shows the stability curve and the variation of composite performance as a func-

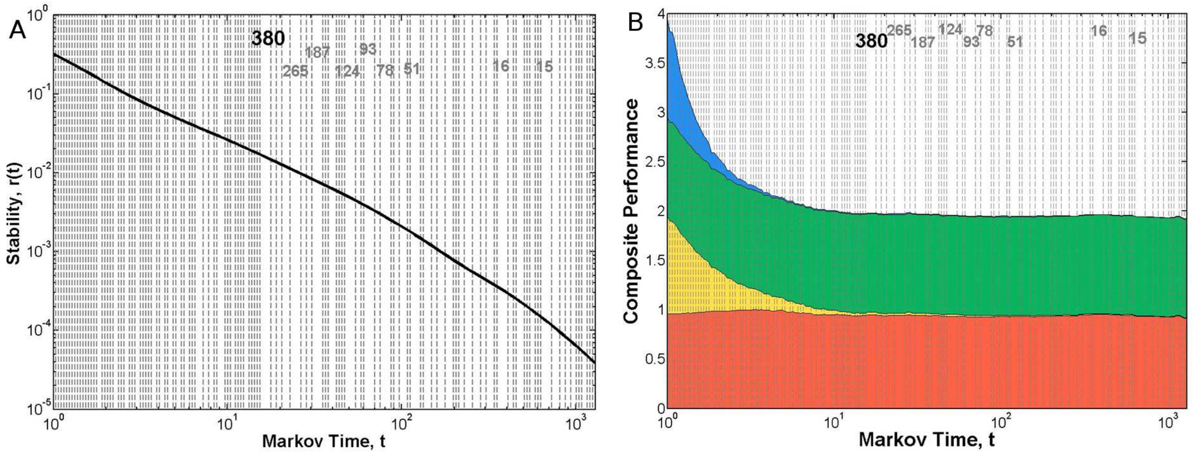

Figure 35: Diffusion based Laplacian modularity applied to Amazon co-purchasing network. *A: Stability curve for the co-purchasing network B: Variation of composite performance as a function of the stable communities identified by the stability curve over time shown as an area chart. The red corresponds to community quality, yellow to overlap quality, green to community coverage and blue to overlap coverage. The number of communities in the partitions with high persistence is shown above the composite performance index curve. The number of communities in the partition used for evaluation in the main manuscript is highlighted in bold. Communities with higher composite performance index than the one used for evaluation are identified.*

tion of the stable communities identified by the stability curve for the air transport network. We also analyzed the spatial proximity of the identified overlapping communities. A voronoi diagram was constructed using the geographic coordinates of all airports. Then we evaluated the adjacency of overlapping nodes according to the voronoi diagram. An overlapping node was deemed to be spatially adjacent if all communities it belonged to were adjacent, i.e., each pair of communities shared at least one edge in the voronoi diagram. 97% of all overlapping nodes were spatially adjacent according to DLM, compared to 91% for OSLOM, 71% for link communities and 64% for the best partition according to the clique percolation method. This validates the relevance of the identified overlapping nodes.

6.4.4 Co-purchasing

This network represents the co-purchasing of DVD titles at the site Amazon.com, i.e., DVD's that are purchased simultaneously by the same customer.

Construction The network is taken from www.snap.stanford.edu which contains metadata information related to wide range of products like books, music CD's etc. We consider the information relevant to co-purchasing of DVD's for our network construction. Associated with each DVD, is a set of 5 or less similar co-purchased titles. We build a network using this information, linking two nodes if they are co-purchased. The final strongly connected network consists of

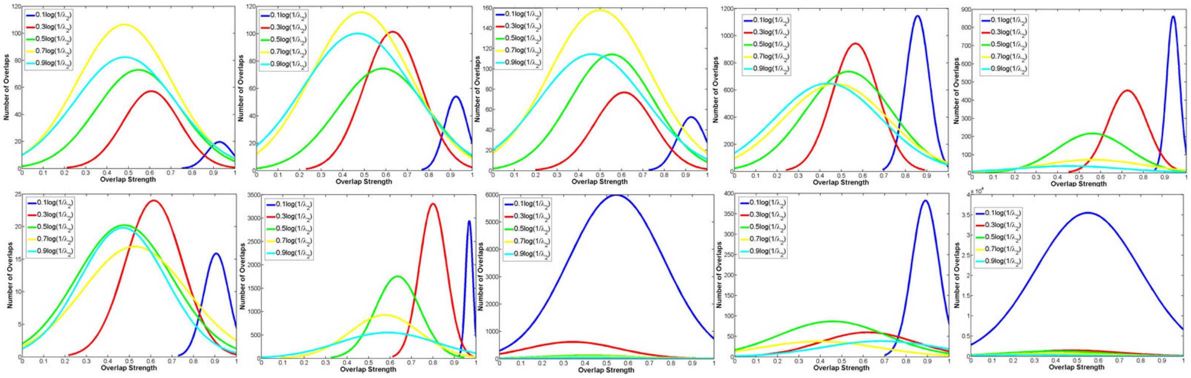

Figure 36: Overlapping landscape strength over time. *Top(Left to Right): S.Cerevisiae, H.sapiens, C.elegans, D.Melanogaster, Facebook networks. Bottom(Left to Right): US congress, Word assoc., Citation, Air transport, Amazon networks*

14436 DVD titles.

Community quality Each DVD is associated with a set of related categories. For eg., the DVD 'NASA' is associated with space exploration, documentary, DVD titles starting with 'N' etc. The similarity between two nodes is evaluated to be equal to the number of categories it shares. The enrichment is then calculated using equation 116

Overlap quality We calculate the mutual information between the number of product categories and the number of non-trivial community memberships for the products.

Figure 35 shows the stability curve and the variation of composite performance as a function of the stable communities identified by the stability curve for the air transport network.

6.4.5 Overlap Landscape

For each of the 10 networks, we investigate the overlapping landscape over time, i.e., we evaluate the number of nodes against strength of overlap over time. Figure 36 shows the overlapping landscape for each of the 10 networks and reveals characteristic patterns in the overlapping community landscape. We fit a curve to the histogram of number of overlapping nodes against the overlap strength which lies between 0 and 1. We consider at 5 values of time as multiples of the relaxation time $1/\lambda_2$: $0.1log(1/\lambda_2), 0.3log(1/\lambda_2), 0.5log(1/\lambda_2), 0.7log(1/\lambda_2), 0.9log(1/\lambda_2))$. A common pattern is that the peaks of the curve gradually shift over time to the left, i.e., at small time most nodes overlap with high strength whereas at longer times, the overlap strength is normalized towards the average of 0.5. Interestingly, the protein interaction networks have more overlaps at long time compared to small time, in contrast to other networks.

7 Results on benchmarks

We compare our method to multiscale extensions of modularity, Infomap using the heat kernel, to spectral methods and finally to community detection methods on benchmark graphs where the partition is planted.

7.1 Comparison with multiscale modularity and multiscale Infomap

We evaluate our method against multiscale extensions of modularity (*LDB14*) and the map equation (*SLB12*) using the heat kernel. The optimal partition was determined as the community with maximal persistence in the stability curve over a logarithmic time range for the multiscale extension of modularity (*LDB14*). The number of communities, K was determined as the value with maximal persistence in the Markov sweeping map plot over a logarithmic time range for the multiscale extension of Infomap (*SLB12*). The optimal partition was determined by optimizing Infomap using the heat kernel at time, $t = e^{(log(t_{int})+log(tend))/2}$. Here t_{int} and t_{end} are the start and end times for which partitions with K number of communities are optimal. The total time range was set as $[0, t_{mix}]$ as done for Laplacian modularity using the heat kernel, which worked best in comparison to other heuristics for all three methods. We see that our method (N) performs better than the other two methods (M (*LDB14*) and I (*SLB12*)) in all, but the karate network in Figure 37 [3]. Also, the multiscale extension of modularity performs better than multiscale InfoMap using H_t. Our analysis indicates that the greedy optimization of the map equation often fails to identify solutions for dense similarity matrices, such as the heat kernel, especially for higher values of t.

We compared our method to the multiscale extensions of modularity and Infomap on the LFR benchmark (*LFR08*) where the ground truth is planted. The parameters used for generating the networks are described in the Figure 38 caption. We plot the value of normalized mutual information (NMI) between the identified communities and ground truth for three values of the average degree: 10,20 and 30. The LFR benchmark is parameterized by μ in this case, which correlates with the strength of communities. For small μ, the communities are fairly obvious whereas as μ tends to 1, the community structure disappears. Figure 38 shows that our method performs better than the multiscale versions of modularity and InfoMap over the entire range of mixing parameter μ for all three values of average degree. The performance of multiscale InfoMap deteriorates as the average degree increases. These experiments validate that the Laplacian modularity is able to better reveal community structure compared to modularity and Infomap when we use the same measure of similarity, i.e., heat kernel.

We also compare the Laplacian modularity using the heat kernel against the multiscale extension of modularity using the heat kernel (*LDB14*) for hierarchical LFR benchmark graphs composed of fine-level micro communities and coarse-level macro-communities. The hierarchical benchmarks have two values of mixing parameters for generating the networks: μ_1, the

[3] We identify the perfect two-way partition in the long-time limit, but is ignored when we operate in time-range, $[0, t_{mix}]$

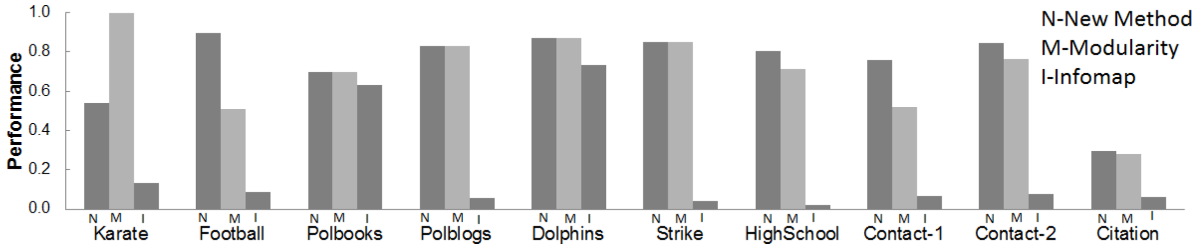

Figure 37: Performance of multiscale community detection methods on real-world networks with known ground truth.*Performance on ten networks with known community assignment measured with the omega index of identified communities against ground truth for the multiscale versions of Laplacian Modularity, Modularity and InfoMap using the heat kernel.*

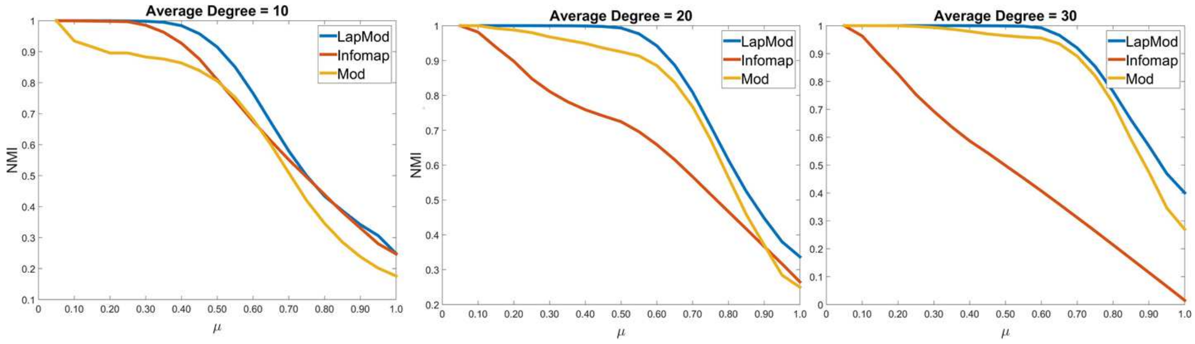

Figure 38: Performance of multiscale community detection methods on undirected and unweighted LFR benchmark graphs without overlapping communities.*The parameters of the graphs are: network Size* $|V| = 1000$, *maximum degree* $k_{max} = 50$, *exponents of the power law distributions are* $\tau_1 = 2$ *for degree and* $\tau_2 = 3$ *for community size, the community sizes are in the range* $[10, 50]$. *We consider three average degree* $\langle k \rangle$ *of nodes: 10,20 and 30 from left to right respectively. The performance metric is the normalized mutual information between the identified communities and ground truth.*

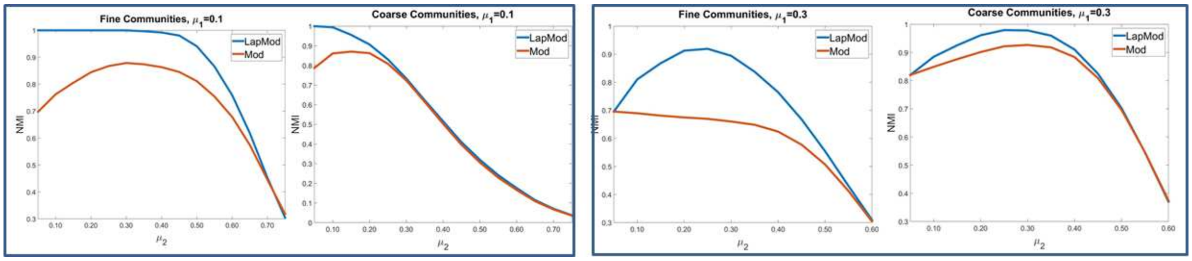

Figure 39: Performance of multiscale community detection methods on hierarchical LFR benchmark graphs (undirected, unweighted, without overlapping communities). *The parameters of the graphs are: network size* $|V| = 2000$*, maximum degree* $k_{max} = 50$*, average degree* $\langle k \rangle = 20$*, exponents of the power law distributions are* $\tau_1 = 2$ *for degree and* $\tau_2 = 1$ *for community size, the micro community sizes are in the range* $[20, 50]$ *and macro community sizes are in the range* $[200, 500]$*. We fix* μ_1 *to be 0.1 and 0.3 respectively and vary* μ_2 *under the constraint* $\mu_1 + \mu_2 = 1$*. The performance metric is the normalized mutual information between the identified fine and coarse scale communities and ground truth.*

fraction of neighbors of each vertex belonging to different macro-communities, and, μ_2, the fraction of neighbors of each vertex belonging to the same macro-community but to different micro-communities (*LDB14*). The coarse and fine level communities are identified as the two maximally persistent communities in the stability curve over a logarithmic time range for both multiscale methods. In Figure 39 we see that the Laplacian modularity is able to better identify both the fine and coarse-level community structure. The optimization of multiscale Infomap was too slow to complete the analysis, however, performed poorer than multiscale modularity on a few examples we tested. All these results indicate that identifying boundaries as done by Laplacian modularity quality function is highly relevant for identifying communities in networks, and the superior performance is not due to the higher computational expense.

7.2 Comparison with spectral methods

Spectral algorithms are a popular class of community detection methods. Spectral algorithms proceed by first finding the eigenvectors corresponding to K largest eigenvalues if the operator is measure of association such as the transition matrix, M, the adjacency matrix A; or the K smallest eigenvalues if the operator is a measure of disassociation or cut such as the Laplacian, L or the random walk Laplacian, $\mathbb{L}$. These eigenvectors assign a $K-$dimensional feature to each node, and the communities are identified using a heuristic such as K-means algorithm on this feature space.

We view spectral algorithms as approximate or relaxed solutions to a discrete objective function. For example the spectra of the adjacency matrix arises as an approximation to the average association, the spectra of the Laplacian arises as an approximation to minimize the

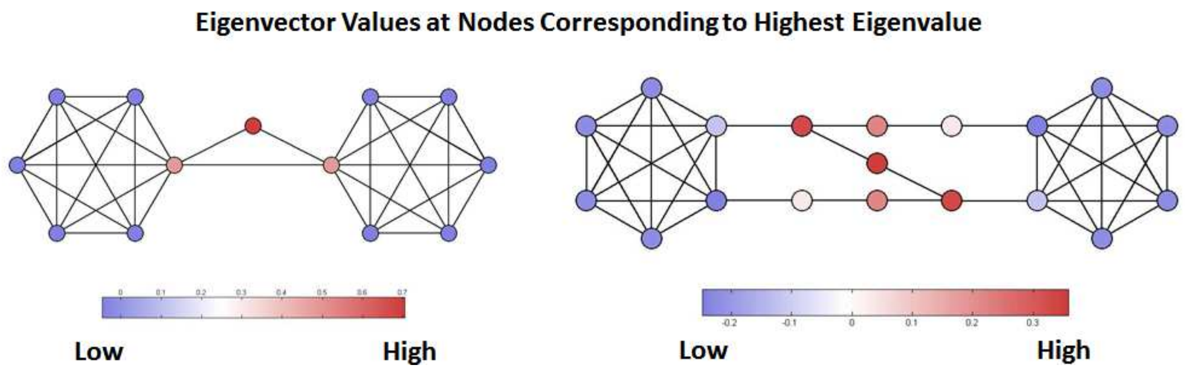

Figure 40: Eigenvector corresponding to largest eigenvalue for the operator $LS+SL$.*The values of the eigenvector corresponding to the largest eigenvalue are color coded for each node. We see that this eigenvector clearly separates boundary nodes from non-boundary nodes.*

ratio cut (*WS03*), the spectra of the random walk Laplacian or the transition matrix arise as an approximation to minimize the normalized cut (*SM00*) or the conductance of the graph, and so on. These objective functions are suitable for partitioning a graph into two or K components, where the parameter K must be known *a priori*. Using the eigengap for identifying K performs poorly as real world networks seldom have a discernable eigengap. In contrast, we view quality functions like modularity as specialized objective functions suitable for community detection as it is capable of directly determining the optimal number of partitions K for a given network.

Laplacian modularity, like modularity, is a quality function capable of optimally partitioning a network and automatically determine K. However, in contrast to all other quality functions, the Laplacian modularity metric is based on identifying boundary links. This is realized as a discrete measure in our paper, but could easily be relaxed to a spectral-type method. For example, Figure 40 shows the eigenvector corresponding to the highest eigenvalue of the matrix $LS + SL$ where S is the adjacency matrix itself. This eigenvector identifies boundaries nodes on the toy networks taken from (*New05*). Thus, our quality function suggests a new class of spectral methods based on properties of $LS + SL$. Our work in this manuscript doesn't pursue this particular line of research as we found that directly optimizing the measure as in the Louvain method sufficed, but we think that spectral techniques will be an extremely fruitful area of subsequent work on this idea of boundary links.

That said, we did compare our automated approach with automated spectral clustering approach based on the Hashimoto or non backtracking operator (*KMM$^+$13*). The number of real eigenvalues contained outside the bulk is suggested as an estimate to automatically identify number of communities for the Hashimoto operator. We see in Figure 41 that in all but one network, our method performs better than this automated spectral approach, $H2$. Furthermore, even when we input K to this spectral approach, the Hashimoto operator performs better on only two (karate and strike) out of the ten networks. Note that when we select number of communities based on ground truth, our method also achieves perfect performance on the karate

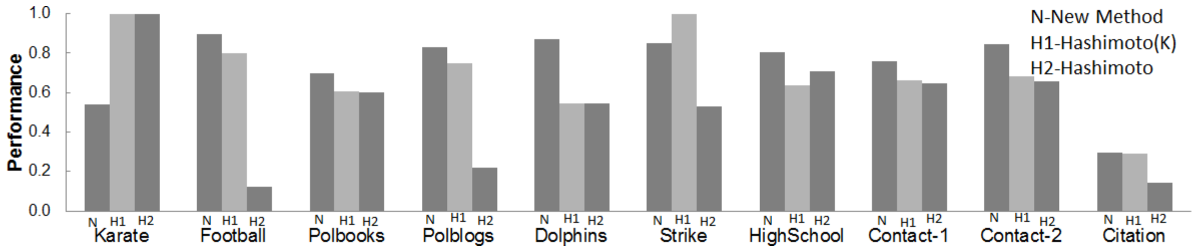

Figure 41: Comparison with spectral community detection using Hashimoto operator on real-world networks.*Performance on ten networks with known community assignment measured with the omega index of identified communities against ground truth for our method, spectral clustering with Hashimoto operator with known K, $H1$, and automated spectral clustering with Hashimoto operator, $H2$.*

and strike network.

We compare our method against a few other spectral methods on the LFR benchmark in Figure 42. We automatically identify communities using our method whereas we input the number of communities K for spectral clustering using K-means for three operators: transition matrix (blue), Hashimoto operator (yellow) and adjacency matrix (red). The LFR benchmark is parameterized by μ in this case, which correlates with the strength of communities. For small μ, the communities are fairly obvious whereas as μ tends to 1, the community structure disappears. We see that our completely automated approach performed better than all spectral approaches for values of μ that indicate strong clustering structure, even though the number of communities was input to these spectral algorithms. The eigengap heuristic for the adjacency and transition matrix or the real eigenvalues separated from bulk heuristic for the Hashimoto operator performed significantly worse.

Finally, we compare our matrix $LS + SL$ to the task that the Hashimoto operator was designed for: finding large scale structure on extremely sparse graphs with an almost regular degree distribution.[4] Although our method is able to identify local community structure, the extreme paucity of boundary links confounds large-scale community identification using our heat kernel based Laplacian modularity quality function. Nevertheless, making a single change addresses this issue. We add a pseudo node that shares links to all nodes in the network. This node acts like a regularizer and helps establish boundaries. With this simple modification, our heat-kernel based Laplacian modularity achieves equivalent results with the Hashimoto matrix on this task (Figure 43).

[4]The generated synthetic networks are unlikely to resemble real world networks which show a scale-free degree distribution.

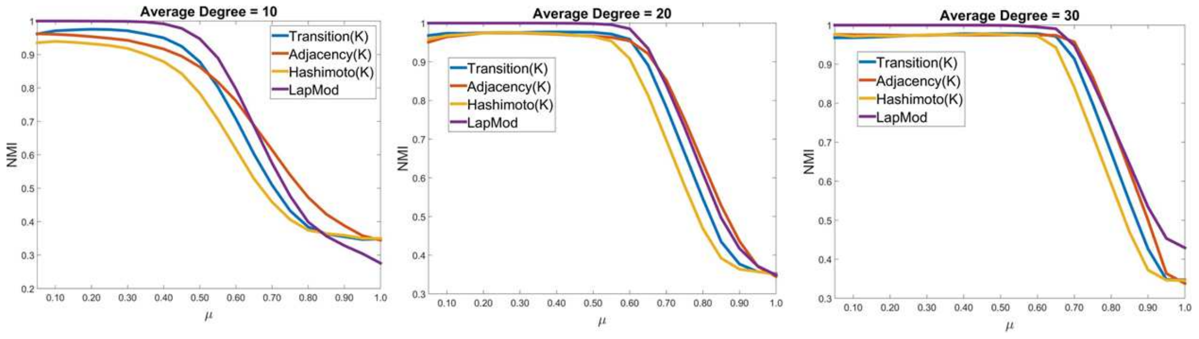

Figure 42: Performance of spectral methods on undirected and unweighted LFR benchmark graphs without overlapping communities.*The parameters of the graphs are: network Size* $|V| = 1000$*, maximum degree* $k_{max} = 50$*, exponents of the power law distributions are* $\tau_1 = 2$ *for degree and* $\tau_2 = 3$ *for community size, the community sizes are in the range* $[10, 50]$*. We consider three average degree* $\langle k \rangle$ *of nodes: 10,20 and 30 from left to right respectively. The performance metric is the normalized mutual information between the identified communities and ground truth.*

7.3 Comparison with community detection methods

We now compare our method against the original InfoMap (*RB08*), Modularity (*New06b*) and OSLOM (*LRRF*) on the LFR benchmarks (*LFR08*). Note that clique percolation (*PDFV05*) and link clustering (*ABL10*) performed worse than all the methods, and hence, not included in our comparison. The parameters used for generating the networks are described in Figure 44 caption. We plot the value of generalized normalized mutual information (NMI) between the identified communities and ground truth for three values of the average degree: 10,20 and 30. Figure 44 shows that our method performs on-par or better than the other methods for small or moderate average degree values, and the performance is significantly better than other methods for large average degree and high values of mixing parameter μ. The performance of InfoMap deteriorates as the average degree increases.

We also compare the Laplacian modularity using the heat kernel against hierarchical community detection methods on hierarchical LFR benchmark graphs composed of fine-level micro communities and coarse-level macro-communities. The coarse and fine level communities are identified as the two maximally persistent communities in the stability curve over a logarithmic time range for the Laplacian modularity using the heat kernel. The top two level partitions were used for OSLOM (*LRRF*) and multilevel InfoMap (*RB11*). In Figure 45 we see that the Laplacian modularity highly competitive for both the fine and coarse-level community structure compared to these two methods. In addition, the stability curve contains rich information about the hierarchical organization of these networks beyond the natural fine and coarse-level partition.

We also compare the Laplacian modularity using the heat kernel against OSLOM (*LRRF*)

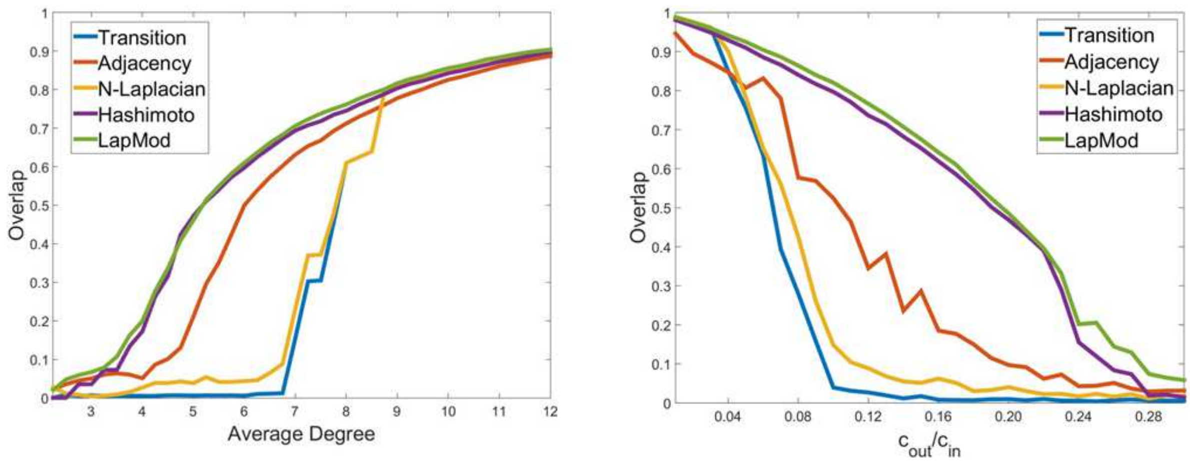

Figure 43: Performance of extremely sparse graphs created by the stochastic block model. *The accuracy of spectral algorithms based on different linear operators, and of Laplacian modularity, for two groups of equal size. On the left, we set c_{out}/c_{in} = 0.3 and vary $\langle k \rangle$; the detectability transition is at $\langle k \rangle$ 3.45 (see (KMM$^+$13)). On the right, we vary c_{out}/c_{in} while fixing the average degree $\langle k \rangle = 3$; the detectability transition occurs at c_{out}/c_{in} = 0.268 (KMM$^+$13). Each point is averaged over 20 instances with $|V| = 10^5$. Our Laplacian modularity quality function achieves slightly better performance than the non-backtracking or Hashimoto matrix , and both remain large all the way down to the transition. Standard spectral algorithms based on the adjacency matrix, normalized Laplacian matrix, and the random walk transition matrix fail well above the transition as shown in (KMM$^+$13).*

for overlapping community detection. We use the $X^T B_t$ metric described in section to detect overlap and the threshold parameter ϵ was set to 0.1 in all our experiments. In Figure 46 we show how the performance of each method decays with the fraction of overlapping nodes, for two different choices of the mixing parameter among communities. The fraction of overlap ranged from 0 where the ground truth is a hard partition (no overlap) to 1 where every node is overlapping with membership to 2 communities. The other parameters for generating the network are mention in the caption of Figure 46 left. We also consider the case where each node may belong to more than two communities. The LFR benchmark has a provision to change the number of memberships of the nodes, although all overlapping nodes are constrained to have the same number of memberships which is unlikely in real-world networks. From Figure 46 left we deduce that our method performs slightly better when there is pervasive overlap, however, the performance of OSLOM is slightly better as membership increases. We contend that setting a different threshold parameter by evaluating the local statistics of each node, instead of a constant threshold as done in our experiments, will further improve our results on benchmarks.

The performance on these benchmarks, which albeit do not possess several properties of real-world networks (*ABL10*), coupled with the superior performance on real-world networks,

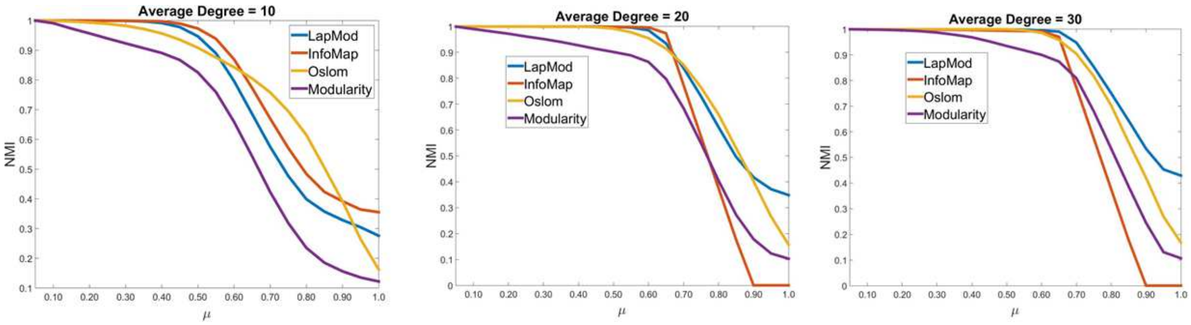

Figure 44: Performance of community detection methods on undirected and unweighted LFR benchmark graphs without overlapping communities. *The parameters of the graphs are: network Size* $|V| = 1000$*, maximum degree* $k_{max} = 50$*, exponents of the power law distributions are* $\tau_1 = 2$ *for degree and* $\tau_2 = 3$ *for community size, the community sizes are in the range* $[10, 50]$*. We consider three average degree* $\langle k \rangle$ *of nodes: 10,20 and 30 from left to right respectively. The performance metric is the normalized mutual information between the identified communities and ground truth.*

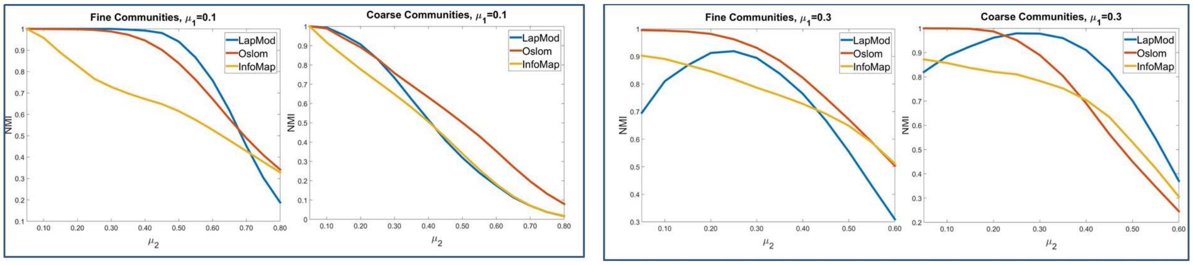

Figure 45: Performance of community detection methods on hierarchical LFR benchmark graphs (undirected, unweighted, without overlapping communities). *The parameters of the graphs are: network size* $|V| = 2000$*, maximum degree* $k_{max} = 50$*, average degree* $\langle k \rangle = 20$*, exponents of the power law distributions are* $\tau_1 = 2$ *for degree and* $\tau_2 = 1$ *for community size, the micro community sizes are in the range* $[20, 50]$ *and macro community sizes are in the range* $[200, 500]$*. We fix* μ_1 *to be 0.1 and 0.3 respectively and vary* μ_2 *under the constraint* $\mu_1 + \mu_2 = 1$*. The performance metric is the normalized mutual information between the identified fine and coarse scale communities and ground truth.*

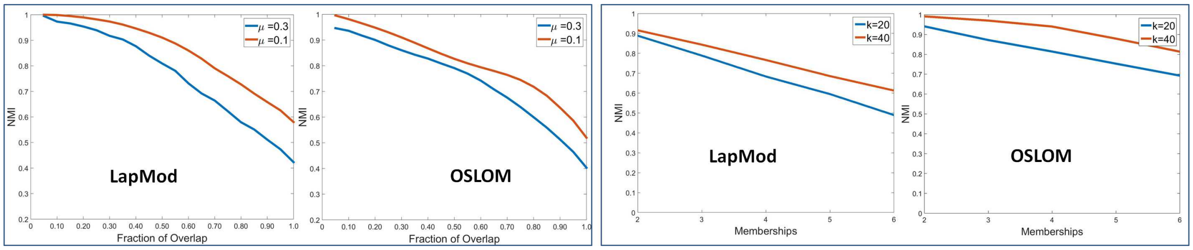

Figure 46: Performance on undirected and unweighted LFR benchmark with overlapping communities. *The parameters of the graphs are: network size* $|V| = 1000$*, maximum degree* $k_{max} = 50$*, average degree* $\langle k \rangle = 20$*, exponents of the power law distributions are* $\tau_1 = 2$ *for degree and* $\tau_2 = 1$ *for community size, the community sizes are in the range* $[20, 50]$*. We fix the mixing parameter* μ *to be 0.1 and 0.3, respectively, the number of memberships to be 2, and vary the fraction of overlapping nodes for the first plot, and fix the average degree* $\langle k \rangle$ *to be 20 and 40, the fraction of overlapping nodes to 0.1, and vary the number of memberships of each node in the second plot.*

gives us confidence that the Laplacian modularity quality function which focuses on identifying boundaries can identify communities in a broad range of networks.

8 Laplacian modularity applied to large networks and for alternate measures of connectivity potentials

As a concluding remark, we demonstrate the Laplacian modularity framework for some large networks and alternate definitions governing the connectivity potential, S.

8.1 Laplacian modularity applied to large networks

We investigate the Laplacian modularity on three large networks taken from www.snap.stanford.edu which have corresponding ground truth community information. We use transition matrix M as a measure of similarity instead of the heat kernel, for computational efficiency. This corresponds to the discrete time random walk for time, $t = 1$. We use the generalized NMI metric to evaluate community quality against the ground truth (*LFK09*). The methods tested are greedy modularity optimization, Infomap, both scalable to large networks, and finally DLM optimization at $t = 1$ for discrete random walks. The computational complexity of probability evaluation is $\mathcal{O}(cE)$, and hence, scalable to large networks like the Youtube network which contains more than a million nodes. We see in Figure 47 that DLM optimization is the best performing method for the Amazon and DBLP networks, while all three methods perform poorly on the Youtube network. We examine the statistics of community size distribution in these three networks in

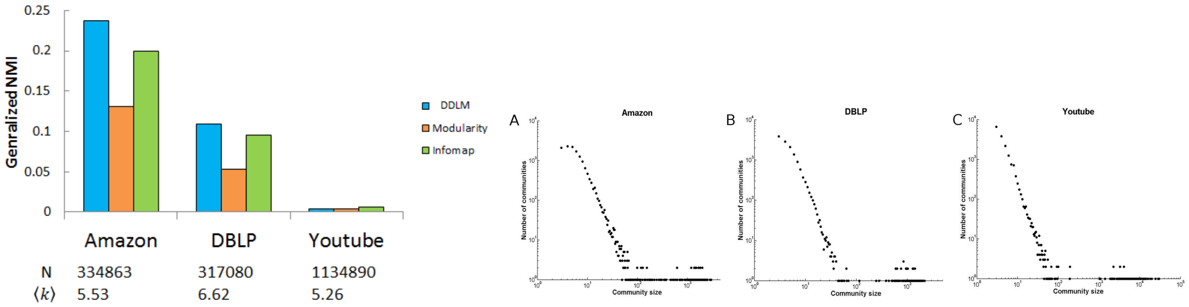

Figure 47: Diffusion based Laplacian modularity applied to large networks. *Left: Performance on networks measured as generalized normalized mutual information of identified communities against ground truth for the 3 large networks. Tested algorithms are discrete diffusion based Laplacian modularity (DDLM) optimization corresponding to* $t = 1$ *for discrete random walks; greedy modularity optimization and Infomap. Table below lists the number of nodes, N, and the average node degree,* $\langle k \rangle$ *for each network. Diffusion based Laplacian modularity optimization finds higher quality communities relative to other methods. Right: Community size distribution follows a scale-free law in the A: Amazon, B: DBLP, and C: Youtube networks*

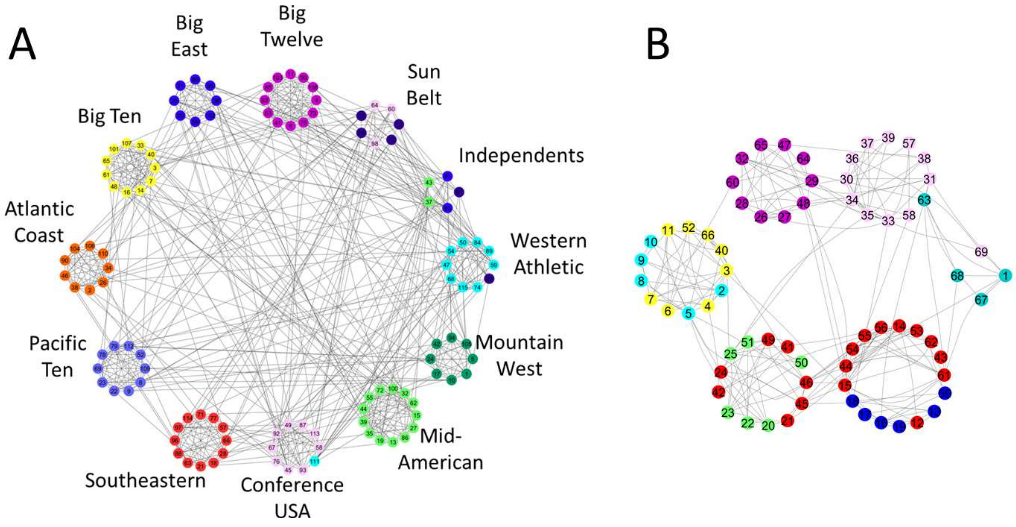

Figure 48: Laplacian modularity for alternate definitions of similarity. *A: Each circular layout of nodes corresponds to one ground truth community in the football network. The communities identified by setting the similarity between two nodes to be equal to the number of common neighbors in the Laplacian modularity quality function are color coded B: Each circular layout of nodes corresponds to one ground truth community in the highschool network. The communities identified by setting the similarity between two nodes to be equal to Newman-Leicht similarity in the Laplacian modularity quality function are color coded*

Figure 47B. The community size distribution is heavy tailed for all networks, confirming the relevance of the communities detected.

8.2 Laplacian modularity for alternate definitions of connectivity potentials

The flexibility in the choice of S in the Laplacian modularity framework enables one to choose a similarity metric that is well-suited for the problem. Although a thorough study of the choice of S is beyond the scope of this work, we show some results of our method for other choices of S commonly used for link prediction methods. We use the networks with known ground truth as described in the main manuscript. We consider the following scenarios for the connectivity potential matrix S:

- Random Walk: Here we choose S to be the random walk transition matrix defined as $D^{-1}A$, where D is a diagonal matrix of degrees and A is the adjacency matrix.
- Preferential Attachment: S is simply $d^T d$, i.e., the degree vector d multiplied by its transpose.
- Common-Neighbor: Here S is A^2 , which represents the number of common neighbors between 2 nodes.
- Cosine Similarity: The cosine similarity matrix is defined as $1 - Cos_{dist}$, i.e, it is one minus the cosine distance between the connectivity vector (row in the adjacency matrix) of nodes (*LNK03*).
- Jaccard Similarity: The Jaccard similarity matrix is defined as $1 - Jac_{dist}$, i.e, it is one minus the jaccard distance between the connectivity vector (row in the adjacency matrix) of nodes in the network (*LNK03*).
- Adamic-Adar: The Adamic-Adar (*AA01*) similarity is defined to be the log of the inverse frequency of shared nodes. This refines the simple counting of common nodes by weighting low degree nodes more heavily.
- Resource-Allocation: The similarity index is defined as the amount of resource a node receives from other nodes through indirect links and each intermediate link contributes an unit of resource (*ZLZ09*).
- $A^2 + \alpha A^3$: This similarity is defined as a weighted combination of immediate neighbors as 2-hop neighbors of a node. We set $\alpha = 0.001$
- SimRank: The similarity is a fixed point of the following recursive definition: two nodes are similar to the extent that they are joined to similar neighbors (*JW02*).

- Newman-Leicht: It is a measure of similarity based on the concept that two vertices are similar if their immediate neighbors in the network are themselves similar (*LHN06b*). This leads to a self-consistent matrix formulation of similarity that can be evaluated iteratively using only a knowledge of the adjacency matrix of the network.

	**Karate**	**Football**	**Polbook**	**Polblog**	**Dolphin**
**Heat-Kernel**	*0.541*	*0.897*	*0.699*	*0.810*	*0.872*
**Random Walk**	**0.264**	0.824	0.145	**0.145**	0.166
**Pref-Attach**	-0.016	0.011	-0.037	0.000	0.017
**Common-Neighbor**	0.000	**0.875**	0.122	0.012	0.029
**Cosine-Sim**	-0.011	**0.875**	0.254	**0.366**	**0.376**
**Jaccard-Sim**	-0.011	0.857	0.179	0.062	0.068
**Adamic-Adar**	0.004	0.853	0.136	0.008	0.096
**Resource-Alloc**	**0.020**	0.853	0.012	0.004	0.015
$A^2 + \alpha A^3$	0.000	**0.875**	0.163	0.008	0.073
**SimRank**	-0.011	0.757	**0.308**	0.056	**0.317**
**Newman-Leicht**	-0.010	**0.897**	**0.333**	0.004	0.225

Table 1: Performance (omega index) on networks with known ground truth for different choices of connectivity potential matrix S. The two best values for each network are highlighted. The first row are the values using the multi-scale heat kernel for reference.

	**Strike**	**Highschool**	**Contact1**	**Contact2**	**Citation**
**Heat-Kernel**	*0.849*	*0.804*	*0.758*	*0.847*	*0.298*
**Random Walk**	**0.364**	0.302	**0.756**	**0.768**	0.036
**Pref-Attach**	-0.070	-0.001	0.008	0.006	-0.001
**Common-Neighbor**	-0.006	0.139	0.157	0.274	0.069
**Cosine-Sim**	0.022	0.060	0.488	0.293	**0.110**
**Jaccard-Sim**	0.022	**0.364**	0.515	0.309	0.088
**Adamic-Adar**	-0.017	0.170	0.157	0.275	0.069
**Resource-Alloc**	0.222	0.239	0.509	0.542	0.069
$A^2 + \alpha A^3$	-0.006	0.098	0.152	0.273	0.097
**SimRank**	0.048	0.140	0.408	0.396	**0.112**
**Newman-Leicht**	**0.798**	**0.598**	**0.680**	**0.759**	0.047

Table 2: Performance (omega index) on networks with known ground truth for different choices of connectivity potential matrix S. The two best values for each network are highlighted. The first row are the values using the multi-scale heat kernel for reference.

As we see in the tables above, different similarity metrics have varying degree of performance in terms of omega index on the different networks. The Random Walk similarity performs consistently well across all datasets. This is natural as the random walk matrix is a

discretized version of the heat kernel for $t = 1$. We see that preferential attachment is a poor choice of similarity across datasets indicating that degree based similarity without considering connectivity information is not revealing of network structure. Similarly, Adamic-Adar performs poorly presumably because it obfuscates connectivity information by assigning higher weight to low degree nodes. Although useful for link prediction, this approach is not beneficial for community detection. We see that cosine similarity to be a simple but effective connectivity potential. Higher order connectivity potentials such as in the Newman-Leicht matrix further boosts performance.

We further evaluate the community structure for the football network by setting the connectivity potential between two nodes to be equal to the number of common neighbors in the Laplacian modularity quality function. The similarity values are efficiently evaluated by the matrix A^2, i.e., the similarity between two nodes is $S(i, j) = A^2(i, j)$ for $i \neq j$ and we set $S(i, i) = 0 \forall i$. Figure 48 A shows the community structure for this similarity metric. The corresponding community quality is slightly inferior than the DLM and Infomap methods, and better than the other four community detection methods. We also evaluate the community structure of the highschool network by setting the connectivity potential between two nodes to be equal to the Newman-Leicht similarity for $\alpha = 0.85$ (*LHN06b*). Figure 48 B shows the community structure for this similarity metric. The quality of the corresponding communities in terms of the omega index is only slightly inferior than the communities identified by DLM, and better than the remaining five community detection methods. Note that all results in the tables, except row 1 are single resolution analysis and cannot capture the hierarchical nature of community structure. Consequently, we proposed the multi-resolution Laplacian, a method to derive stability curves for any choice of connectivity potential discussed earlier.

Multi-resolution Laplacian: In order to validate the generality of the DLM framework to similarity measures beyond the heat kernel, we apply the multi-resolution Laplacian modularity using two alternate measures of similarity. In the contact-1 network, we use the adjacency matrix with self-loops, $S = A + I$ where I is the identity matrix and use the cosine similarity measure between nodes in the contact-2 network. The stability curves corresponding to these similarity measures is shown in Figure 49. The curves are constructed by varying the Laplacian resolution parameter ω in $L = D - \omega A$, i.e., the multi-resolution extension of Laplacian modularity as discussed in section 2.7. ω was continuously varied from 0 till we obtain a partition wherein all nodes are assigned independent communities. The range of ω for finding the most relevant partition was empirically set to be in the range $[[\omega_{int}, \omega_{fin}]$ where ω_{int} is the value of ω for which we obtain a 2-way partition and ω_{fin} is the value of ω for which we obtain a $|V|/3$-way partition. $|V|$ is the number of nodes in the network, and we use $|V|/3$ because a community is composed of atleast 3 nodes. The relevance of a k=way partition was set equal to the length of time window, i.e., equal to the difference of ω_{end} and ω_{start} where ω_{start} and ω_{end} are the start and end values of the parameter ω over which a given partition is optimal. Note that the scale is linear unlike the logarithmic time scale in the heat kernel. This validates the efficacy of Laplacian modularity to detect multi-scale communities using general similarity measures. A more thorough investigation using alternate similarity measures, such as those used for link pre-

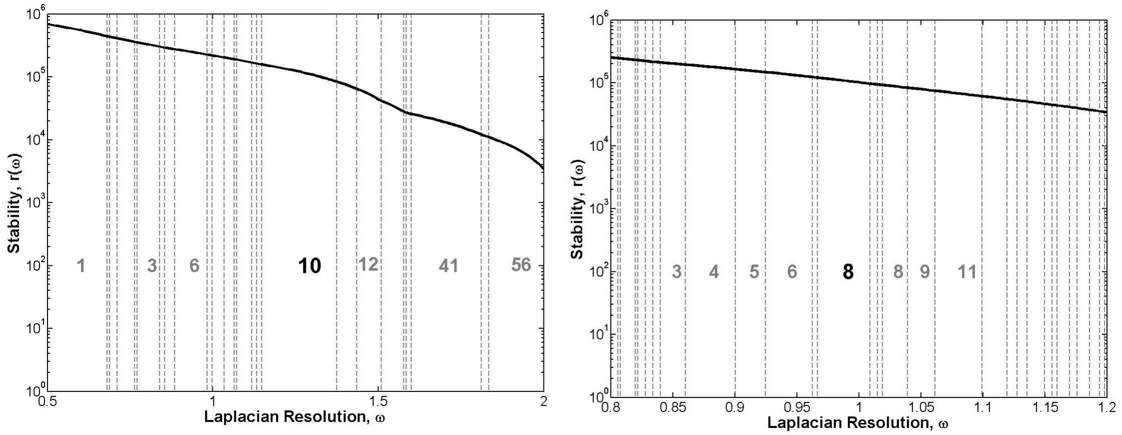

Figure 49: Stability curves for multi-resolution Laplacian modularity. *Left: Stability curve for the contact1 network using $S = I + A$, i.e., considering the adjacency matrix with self-loops as the node similarity matrix. Right: Stability curve for the contact2 network using cosine similarity.*

diction, is suggested as future work. The stability curve corresponding to $S = I + A$, identifies the 10-way partition to be maximally persistent over the range of resolution parameter, ω, and has the highest community quality in terms of the normalized mutual information, better than all other methods (including DLM). The 8-way partition as identified by the cosine similarity metric on the contact-2 network has a higher community than all methods apart from DLM.

Similarity between nodes in a network is often intuitively resolved, whereas the associated community structure is non-trivial and difficult to anatomize. The power of the Laplacian modularity framework lies in efficient resolution of community structure given a metric of similarity, local or global, consistent with our notion of node-to-subgraph cohesion. We illustrate this framework using different metrics of similarity in order to uncover communities. For a general node-to-node similarity matrix, S, we also demonstrate that the multi-resolution Laplacian, $L_\omega = D - \omega A$, in lieu of the standard Laplacian, L, facilitates the identification of relevant communities. Although, we investigated the Laplacian modularity for different metrics of connectivity, its full potential using system-specific similarity metrics remains unexplored.

References and Notes

AA01. Lada A. Adamic and Eytan Adar. Friends and neighbors on the web. *SOCIAL NETWORKS*, 25:211–230, 2001.

ABL10. Y.-Y. Ahn, J. P. Bagrow, and S. Lehmann. Link communities reveal multiscale complexity in networks. *Nature*, 466:761–764, 2010.

ADGPV06. Alex Arenas, Albert Diaz-Guilera, and Conrad J. Pérez-Vicente. Synchronization reveals topological scales in complex networks. *Phys. Rev. Lett.*, 96:114102, Mar 2006.

AFG08. A Arenas, A Fernndez, and S Gmez. Analysis of the structure of complex networks at different resolution levels. *New Journal of Physics*, 10(5):053039, 2008.

AG05. Lada A. Adamic and Natalie Glance. The political blogosphere and the 2004 u.s. election: Divided they blog. In *Proceedings of the 3rd International Workshop on Link Discovery*, KDD '05, pages 36–43, New York, NY, USA, 2005. ACM.

Alv07. Nelson A. Alves. Unveiling community structures in weighted networks. *Physical Review E*, 76:036101, Sep 2007.

AM13. Rodrigo Aldecoa and Ignacio Marín. Surprise maximization reveals the community structure of complex networks. In *Scientific reports*, 2013.

BDG+08. Ulrik Brandes, Daniel Delling, Marco Gaertler, Robert Gorke, Martin Hoefer, Zoran Nikoloski, and Dorothea Wagner. On modularity clustering. *IEEE Transactions on Knowledge and Data Engineering*, 20(2):172–188, 2008.

BGLL08. Vincent D. Blondel, Jean-Loup Guillaume, Renaud Lambiotte, and Etienne Lefebvre. Fast unfolding of communities in large networks. *Journal of Statistical Mechanics: Theory and Experiment*, 2008(10):P10008+, July 2008.

BHLP11. Jonathan W. Berry, Bruce Hendrickson, Randall A. LaViolette, and Cynthia A. Phillips. Tolerating the community detection resolution limit with edge weighting. *Physical Review E*, 83(5):056119+, May 2011.

CD88. L. M. Collins and C. W. Dent. Omega: A general formulation of the rand index of cluster recovery suitable for non-disjoint solutions. *Multivariate Behavioral Research*, 23(2):231–242, 1988.

Chu97. F. R. K. Chung. *Spectral Graph Theory*. American Mathematical Society, 1997.

Chu07. Fan Chung. The heat kernel as the pagerank of a graph. *Proceedings of the National Academy of Sciences*, 104(50):19735–19740, 2007.

cit98. *An information-theoretic definition of similarity*. Morgan Kaufmann, San Francisco, CA, 1998.

CNS13. Mingming Chen, Tommy Nguyen, and Boleslaw Szymanski. A new metric for quality of network community structure. 2:15, 09 2013.

CSB18. Tianlong Chen, Pramesh Singh, and Kevin E Bassler. Network community detection using modularity density measures. *Journal of Statistical Mechanics: Theory and Experiment*, 2018(5):053406, 2018.

DYB10. J. C. Delvenne, S. N. Yaliraki, and M. Barahona. Stability of graph communities across time scales. *Proceedings of the National Academy of Sciences*, 107(29):12755–12760, July 2010.

FB07. Santo Fortunato and Marc Barthélemy. Resolution limit in community detection. *Proceedings of the National Academy of Sciences*, 104(1):36–41, January 2007.

FCF. Adrien Friggeri, Guillaume Chelius, and Eric Fleury. Egomunities, exploring socially cohesive person-based communities. *CoRR*, abs/1102.2623.

Fol06. James Folwer. Connecting the Congress: A Study of Cosponsorship Networks. *Political Analysis*, 14(4):456–487, 2006.

For10. Santo Fortunato. Community detection in graphs. *Physics Reports*, 486(3):75–174, 2010.

GN02. M. Girvan and M. E. J. Newman. Community structure in social and biological networks. *Proceedings of the National Academy of Sciences*, 99(12):7821–7826, June 2002.

Gre07. Steve Gregory. An algorithm to find overlapping community structure in networks. In JoostN. Kok, Jacek Koronacki, Ramon Lopez de Mantaras, Stan Matwin, Dunja Mladenic, and Andrzej Skowron, editors, *Knowledge Discovery in Databases: PKDD 2007*, volume 4702 of *Lecture Notes in Computer Science*, pages 91–102. Springer Berlin Heidelberg, 2007.

hig. *This work uses data from Add Health, a program project designed by Udry J., Bearman S., and Harris, Kathleen Mullan, and funded by a grant P01-HD31921 from the National Institute of Child Health and Human Development, with cooperative funding from 17 other agencies. Special acknowledgment is due Ronald R. Rindfuss and Barbara Entwisle for assistance in the original design. Persons interested in obtaining data files from Add Health should contact Add Health, Carolina Population Center, 123 W. Franklin Street, Chapel Hill, NC 275162524 (addhealth@unc.edu).*

HTF01. Trevor Hastie, Robert Tibshirani, and Jerome Friedman. *The Elements of Statistical Learning*. Springer Series in Statistics. Springer New York Inc., New York, NY, USA, 2001.

JW02. Glen Jeh and Jennifer Widom. Simrank: A measure of structural-context similarity. In *Proceedings of the Eighth ACM SIGKDD International Conference on Knowledge Discovery and Data Mining*, KDD '02, pages 538–543, New York, NY, USA, 2002. ACM.

JW03. Glen Jeh and Jennifer Widom. Scaling personalized web search. In *Proceedings of the 12th International Conference on World Wide Web*, WWW '03, pages 271–279, New York, NY, USA, 2003. ACM.

Kei79. Julian Keilson. *Markov chain models-Rarity and Exponentiality*. Number 28 in Applied mathematical sciences. Springer, New York, USA, 1979.

KG13. Kyle Kloster and David F. Gleich. A nearly-sublinear method for approximating a column of the matrix exponential for matrices from large, sparse networks. In Anthony Bonato, Michael Mitzenmacher, and Pawe Praat, editors, *Algorithms and Models for the Web Graph*, volume 8305 of *Lecture Notes in Computer Science*, pages 68–79. Springer International Publishing, December 2013.

KL13. Jan O. Korbel and Charles Lee. Genome assembly and haplotyping with Hi-C. *Nat Biotech*, 31(12):1099–1101, December 2013.

KMM$^+$13. Florent Krzakala, Cristopher Moore, Elchanan Mossel, Joe Neeman, Allan Sly, Lenka Zdeborov, and Pan Zhang. Spectral redemption in clustering sparse networks. *Proceedings of the National Academy of Sciences*, 110(52):20935–20940, 2013.

KS60. J.G. Kemeny and J.L. Snell. *Finite Markov Chains*. Undergraduate Texts in Mathematics. Springer-Verlag, 1960.

KSJ10. Youngdo Kim, Seung-Woo Son, and Hawoong Jeong. Finding communities in directed networks. *Physical Review E*, 81:016103, Jan 2010.

LCM98. Claudia Leacock, Martin Chodorow, and George A. Miller. Using Corpus Statistics and WordNet Relations for Sense Identification. *Association for Computational Linguistics*, 1998.

LDB14. Renaud Lambiotte, Jean-Charles Delvenne, and Mauricio Barahona. Random walks, markov processes and the multiscale modular organization of complex networks. *Network Science and Engineering, IEEE Transactions on*, 1(2):76–90, 2014.

LFK09. Andrea Lancichinetti, Santo Fortunato, and János Kertész. Detecting the overlapping and hierarchical community structure in complex networks. *New Journal of Physics*, 11(3):033015+, March 2009.

LFR08. Andrea Lancichinetti, Santo Fortunato, and Filippo Radicchi. Benchmark graphs for testing community detection algorithms. *Phys. Rev. E*, 78:046110, Oct 2008.

LHN06a. E. A. Leicht, Petter Holme, and M. E. J. Newman. Vertex similarity in networks. *Phys. Rev. E*, 73:026120, Feb 2006.

LHN06b. E. A. Leicht, Petter Holme, and M. E. J. Newman. Vertex similarity in networks. *Phys. Rev. E*, 73:026120, Feb 2006.

LL10. Jian Liu and Tingzhan Liu. Detecting community structure in complex networks using simulated annealing with k-means algorithms. *Physica A: Statistical Mechanics and its Applications*, 389(11):2300–2309, February 2010.

LM97. Judd H. LMichael and Joseph G. Massey. Modeling the communication network in a sawmill. *Forest Products Journal*, 47(9):p25, 1997.

LN08. E. A. Leicht and M. E. J. Newman. Community structure in directed networks. *Phys. Rev. Lett.*, 100(11):118703, March 2008.

LNK03. David Liben-Nowell and Jon Kleinberg. The link prediction problem for social networks. In *Proceedings of the Twelfth International Conference on Information and Knowledge Management*, CIKM '03, pages 556–559, New York, NY, USA, 2003. ACM.

Lov93. László Lovász. *Random Walks on Graphs: A Survey*, pages 353–397. 1993.

Lov96. L. Lovász. Random walks on graphs: A survey. volume 2, pages 353–398. János Bolyai Mathematical Society, Budapest, 1996.

LRRF. Andrea Lancichinetti, Filippo Radicchi, José J. Ramasco, and Santo Fortunato. Finding Statistically Significant Communities in Networks. *PLoS ONE*, 6(5).

LS69. F. Luccio and M. Sami. On the decomposition of networks in minimally interconnected subnetworks. *Circuit Theory, IEEE Transactions on*, 16(2):184–188, May 1969.

LSB$^+$03. David Lusseau, Karsten Schneider, Oliver J Boisseau, Patti Haase, Elisabeth Slooten, and Steve M Dawson. The bottlenose dolphin community of doubtful sound features a large proportion of long-lasting associations. *Behavioral Ecology and Sociobiology*, 54(4):396–405, 2003.

MRM$^+$10. Peter J. Mucha, Thomas Richardson, Kevin Macon, Mason A. Porter, and Jukka-Pekka Onnela. Community structure in time-dependent, multiscale, and multiplex networks. *Science*, 328(5980):876–878, 2010.

New04a. M. E. J. Newman. Analysis of weighted networks. *Physical Review E*, 70(5):056131, November 2004.

New04b. M. E. J. Newman. Fast algorithm for detecting community structure in networks. *Physical Review E*, 69:066133, Jun 2004.

New05. M. E. J. Newman. A measure of betweenness centrality based on random walks. *Social Networks*, 2005.

New06a. M. E. J. Newman. Finding community structure in networks using the eigenvectors of matrices. *Physical Review E*, 74(3):036104+, July 2006.

New06b. M. E. J. Newman. Modularity and community structure in networks. *Proceedings of the National Academy of Sciences*, 103(23):8577–8582, June 2006.

New10. Mark Newman. *Networks: An Introduction*. Oxford University Press, New York, NY, USA, 2010.

NMCM09. V Nicosia, G Mangioni, V Carchiolo, and M Malgeri. Extending the definition of modularity to directed graphs with overlapping communities. *Journal of Statistical Mechanics: Theory and Experiment*, 2009(03):P03024, 2009.

NMS98. D. L. Nelson, C. L. McEvoy, and T. A Schreiber. *The University of South Florida word association, rhyme, and word fragment norms*, 1998.

OSFM07. R. Olfati-Saber, J.A. Fax, and R.M. Murray. Consensus and cooperation in networked multi-agent systems. *Proceedings of the IEEE*, 95(1):215–233, Jan 2007.

PDFV05. G. Palla, I. Derenyi, I. Farkas, and T. Vicsek. Uncovering the overlapping community structure of complex networks in nature and society. *Nature*, 435:814–818, 2005.

PL04. P. Pons and M. Latapy. Computing communities in large networks using random walks. *J. Graph Algorithms Appl.*, 10(2):284–293, 2004.

QH07. Huaijun Qiu and Edwin R. Hancock. Clustering and embedding using commute times. *IEEE Trans. Pattern Anal. Mach. Intell.*, 29(11):1873–1890, 2007.

RB03. Erzsébet Ravasz and Albert-László L. Barabási. Hierarchical organization in complex networks. *Physical review. E, Statistical, nonlinear, and soft matter physics*, 67(2 Pt 2), February 2003.

RB06. Jörg Reichardt and Stefan Bornholdt. Statistical mechanics of community detection. *Phys. Rev. E*, 74:016110, Jul 2006.

RB08. Martin Rosvall and Carl T. Bergstrom. Maps of random walks on complex networks reveal community structure. *Proceedings of the National Academy of Sciences*, 105(4):1118–1123, January 2008.

RB11. Martin Rosvall and Carl T. Bergstrom. Multilevel Compression of Random Walks on Networks Reveals Hierarchical Organization in Large Integrated Systems. *PLoS ONE*, 6(4):e18209+, April 2011.

RCC$^+$04. Filippo Radicchi, Claudio Castellano, Federico Cecconi, Vittorio Loreto, and Domenico Parisi. Defining and identifying communities in networks. *Proceedings of the National Academy of Sciences*, 101(9):2658–2663, March 2004.

Rus11. Raif M. Rustamov. Multiscale biharmonic kernels. *Comput. Graph. Forum*, 30(5):1521–1531, 2011.

SDYB12. Michael T. Schaub, Jean-Charles Delvenne, Sophia N. Yaliraki, and Mauricio Barahona. Markov dynamics as a zooming lens for multiscale community detection: Non clique-like communities and the field-of-view limit. *PLoS ONE*, 7(2):e32210, 02 2012.

She13. Hua-Wei Shen. Community structure and diffusion dynamics on networks. In *Community Structure of Complex Networks*, Springer Theses, pages 73–92. Springer Berlin Heidelberg, 2013.

SLB12. Michael T. Schaub, Renaud Lambiotte, and Mauricio Barahona. Encoding dynamics for multiscale community detection: Markov time sweeping for the map equation. *Phys. Rev. E*, 86:026112, Aug 2012.

SLM$^+$04. Clement A. Stanyon, Guozhen Liu, Bernardo A. Mangiola, Nishi Patel, Loic Giot, Bing Kuang, Huamei Zhang, Jinhui Zhong, and Russell L. Finley. A Drosophila protein-interaction map centered on cell-cycle regulators. *Genome biology*, 5(12), 2004.

SM00. Jianbo Shi and J. Malik. Normalized cuts and image segmentation. *IEEE Transactions on Pattern Analysis and Machine Intelligence*, 22(8):888–905, Aug 2000.

SR14. A. Sinha and K. Ramani. Multi-scale kernels using random walks. *Computer Graphics Forum*, 33(1):164–177, 2014.

SVB$^+$11. Juliette Stehl, Nicolas Voirin, Alain Barrat, Ciro Cattuto, Lorenzo Isella, Jean-Franois Pinton, Marco Quaggiotto, Wouter Van den Broeck, Corinne Rgis, Bruno Lina, and Philippe Vanhems. High-resolution measurements of face-to-face contact patterns in a primary school. *PLoS ONE*, 6(8):e23176, 08 2011.

TKMP11. Amanda L. Traud, Eric D. Kelsic, Peter J. Mucha, and Mason A. Porter. Comparing community structure to characteristics in online collegiate social networks. *SIAM Rev.*, 53(3):526–543, August 2011.

TVDN11. V. A. Traag, P. Van Dooren, and Y. Nesterov. Narrow scope for resolution-limit-free community detection. *Phys. Rev. E*, 84:016114, Jul 2011.

WDG15. Joyce Jiyoung Whang, Inderjit S. Dhillon, and David F. Gleich. Non-exhaustive, overlapping k-means. In *Proceedings of the 2015 SIAM International Conference on Data Mining*, pages 936–944, 2015.

WS03. Song Wang and Jeffrey Mark Siskind. Image segmentation with ratio cut. *IEEE Transactions on Pattern Analysis and Machine Intelligence*, 25:675–690, 2003.

Wu04. F. Y. Wu. Theory of resistor networks: the two-point resistance. *Journal of Physics A: Mathematical and General*, 37(26):6653–6673, July 2004.

YBY$^+$08. H. Yu, P. Braun, M. A. Yildirim, I. Lemmens, K. Venkatesan, J. Sahalie, Hirozane T. Kishikawa, F. Gebreab, N. Li, N. Simonis, and Others. High-Quality Binary Protein Interaction Map of the Yeast Interactome Network. *Science*, 322(5898):104, 2008.

YNP12. Haixuan Yang, Tamás Nepusz, and Alberto Paccanaro. Improving go semantic similarity measures by exploring the ontology beneath the terms and modelling uncertainty. *Bioinformatics*, 28(10):1383–1389, May 2012.

YTT$^+$11. Haiyuan Yu, Leah Tardivo, Stanley Tam, Evan Weiner, Fana Gebreab, Changyu Fan, Nenad Svrzikapa, Tomoko Hirozane-Kishikawa, Edward Rietman, Xinping Yang, Julie Sahalie, Kourosh Salehi-Ashtiani, Tong Hao, Michael E. Cusick, David E. Hill, Frederick P. Roth, Pascal Braun, and Marc Vidal. Next-generation sequencing to generate interactome datasets. *Nature methods*, 8(6):478–480, June 2011.

Zac77. Wayne W. Zachary. An information flow model for conflict and fission in small groups. *Journal of Anthropological Research*, 33(4):pp. 452–473, 1977.

ZLZ09. Tao Zhou, Linyuan Lü, and Yi-Cheng Zhang. Predicting missing links via local information. *The European Physical Journal B*, 71(4):623–630, Oct 2009.
